# Cell wash-free fluorescent probes based on phenothiazine and phenoxazine with high photostability and large stokes shifts for targeted imaging of subcellular organelles

**DOI:** 10.1016/j.mtbio.2025.102399

**Published:** 2025-10-10

**Authors:** Zhichao Wang, Jinxiao Lyu, Yongjie Sun, Fang Liu, Lanqing Li, Shaoping Li, Xuanjun Zhang

**Affiliations:** a Faculty of Health Sciences, University of Macau, Taipa, Macao Special Administrative Region of China; b School of Pharmacy and Food Engineering, Wuyi University, Jiangmen, 529020, PR China; c State Key Laboratory of Quality Research in Chinese Medicine, Institute of Chinese Medical Sciences, University of Macau, Macao Special Administrative Region of China; d MOE Frontiers Science Center for Precision Oncology, University of Macau, Taipa, Macao Special Administrative Region of China

**Keywords:** Fluorescent probe, Wash free fluorescent imaging, Phenothiazine and phenoxazine, Organelle-targeting, Polarity sensitive

## Abstract

Most organelle-targeting probes require the removal of excess dye to enhance the signal-to-noise ratio before microscopic imaging experiments. However, this washing step may cause cellular damage and interfere with the continuous observation of cellular activities. Here, we report a series of wash-free probes based on small molecule phenothiazine/phenoxazine. Simple modification of phenothiazine/phenoxazine by nitro group significantly enhances the polarity-sensitive characteristic, which are well-suited for wash-free cellular imaging. These probes feature low molecular weight, excellent photostability, and large Stokes shifts (up to 191 nm). By conjugation with targeting groups, a series of probes are developed for specific imaging in different organelles such as lysosomes, mitochondria, the endoplasmic reticulum, lipid droplets, and the plasma membrane, without any washing step. Moreover, by simply replacing S atom in the central core with O atom, the emission of probes shifts from red to green-yellow. Using these two probes, high-contrast, dual-color wash-free imaging can be achieved. Among them, the PXZ-Lipid probe was successfully applied for real-time monitoring of dynamic changes in lipid droplets within living cells. This work establishes a general strategy for designing small-molecule, wash-free fluorescent probes based on phenothiazine/phenoxazine scaffolds, enabling real-time, multi-organelle imaging with minimal cellular disturbance.

## Highlights

• The probes achieve a pronounced environment-sensitive response through simple modification with the nitro group
• Large Stokes shifts (up to 191 nm) and excellent photostability.
• Specific targeting of lysosomes, mitochondria, ER, lipid droplets, and membrane.
• Real-time imaging of lipid droplet dynamics in living cells without washing.

## Graphical abstract

Nitro-modified phenothiazine/phenoxazine small-molecule fluorescent probes exhibit strong polarity sensitivity, high photostability, large Stokes shifts (up to 191 nm), and low molecular weight. Conjugation with targeting groups enables wash-free, real-time imaging of multiple organelles (lysosomes, mitochondria, ER, lipid droplets, plasma membrane) in living cells. The PXZ-Lipid probe allows dynamic monitoring of lipid droplet changes, providing a general strategy for minimally invasive multi-organelle visualization.

**Figure:**
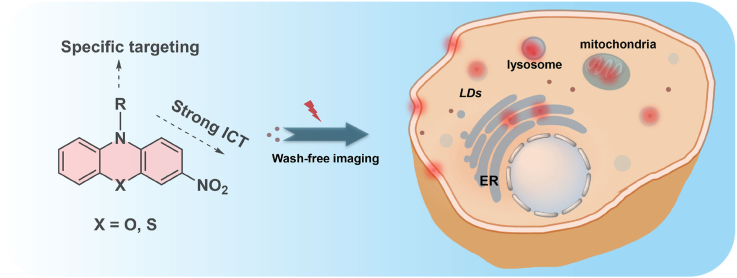

## 1 Introduction

Fluorescence imaging, as a rapidly advancing technology, has garnered significant attention in visualizing cellular structures and dynamic biological processes due to its non-invasiveness, high spatiotemporal resolution, and exceptional sensitivity [[1], [2], [3], [4], [5]]. Fluorescent probes are considered key components of fluorescence imaging systems, and an ideal probe should meet the following criteria: (1) excellent biocompatibility and minimal cytotoxicity; (2) compatibility with wash-free imaging workflows; (3) specific targeting of subcellular organelles or structures; and (4) high photostability, excellent solubility, and a large Stokes shift [[6], [7], [8], [9], [10]]. Although numerous probes with favorable photophysical properties and biocompatibility have been developed, many suffer from nonspecific interactions with intracellular components, resulting in high background fluorescence that compromises imaging quality, necessitating laborious washing steps during experiments [[11], [12], [13], [14]].

Wash-free probes for live-cell imaging not only eliminate time-consuming washing procedures but also enhance spatiotemporal resolution while minimizing harm to cell. Such probes generally function via two mechanisms: (1) reaction-activated and (2) environment-sensitive. Reaction-activated probes undergo structural and photophysical changes upon interaction with target biomolecules, leading to fluorescence signal amplification [[15], [16], [17], [18], [19], [20]]. In contrast, non-activated probes retain their original state, enabling wash-free imaging. Environment-sensitive fluorophore exhibits different photophysical properties in different environments [21,22]. Representative categories include temperature-sensitive fluorophores [[23], [24], [25]], viscosity-sensitive fluorophores [[26], [27], [28], [29], [30]], aggregation-induced emission (AIE) materials [31,32], and polarity-sensitive fluorophores [[33], [34], [35], [36], [37], [38]]. Given the heterogeneous polarity distribution within cells—where hydrophilic cytoplasm (high polarity) and hydrophobic organelles (low polarity)—polarity-sensitive probes are particularly well-suited for wash-free live-cell imaging [39].

Most polarity-sensitive probes adopt a donor–acceptor (D–A) structure, exhibiting solvatochromic effects via intramolecular charge transfer (ICT), where fluorescence is quenched in highly polar solvents [[40], [41], [42], [43], [44], [45]]. However, most molecules with a D-A structure have inconvenient synthetic routes or use charged groups as the acceptor or donor groups to enhance the ICT effect and extend emission spectra [[46], [47], [48], [49]]. Anionic fluorophores often exhibit low cell permeability, while cationic fluorophores tend to interact with the cell membrane, resulting in reduced cell survival rates or accumulation in mitochondria [[50], [51], [52]]. This makes it challenging for them to localize to other organelles or cellular structures.

Phenothiazine, a strong electron-donating group, has been widely utilized in the design and synthesis of fluorescent probes for cellular imaging. Hu's group reported a D–A–D structured molecule based on phenothiazine that enabled specific labeling of lipid droplets under wash-free conditions [53,54]. Moreover, Manab's group developed a D–π–D structured molecule by combining phenothiazine with an n-hexylated-indole-linked anthracene, which was successfully applied in wash-free imaging of cancer cells. [55]. To develop low molecular weight probes capable of targeting multiple organelles, we designed a series of D–A fluorophores with pronounced polarity sensitivity based on the phenothiazine scaffold, achieved through the simple introduction of nitro groups. These fluorophores exhibit low molecular weight, broad Stokes shifts, excellent photostability, and tunable emission spectra. By conjugating organelle-targeting moieties (e.g., lysosomal, mitochondrial, endoplasmic reticulum, plasma membrane, or lipid droplet), the resulting probes enabled wash-free, organelle-specific imaging in live cells. A similar modification strategy applied to phenoxazine derivatives also yielded solvatochromic behavior, enabling wash-free imaging across multiple organelles. Building on their organelle specificity and tunable emission profiles, both phenothiazine and phenoxazine derivatives were further employed in dual-channel imaging to simultaneously visualize distinct subcellular structures.

## 2 Materials and methods

### 2.1 Materials and measurements

All chemicals and reagents from Sigma–Aldrich were used as received from the supplier unless otherwise stated. ^1^H and ^13^C NMR spectra were measured on a Bruker AV-400 MHz NMR spectrometer with chemical shift reported in parts per million (ppm, *δ*). Mass spectra were recorded on Bruker Microflex MALDI-TOF system. Uv–Vis absorption spectra were measured on a Shimadzu UV-1800 spectrometer. Photoluminescence spectra, quantum yields and fluorescence lifetime were conducted on the Horiba FluoroMax-4 spectrofluorometer. The cell culture serum and medium were purchased from Gibco. HeLa and SKOV3 cells were obtained from the Faculty of Health Science, University of Macau. All the cells were incubated in Thermo Fisher Forma Series 3 Water Jacketed CO_2_ incubator. Confocal imaging experiments were evaluated with fluorescence microscope (Carl Zeiss, Germany).

### 2.2 Synthesis of 3-nitro-10H-phenothiazine (**1a**)

Phenothiazine (2.00 g, 10.04 mmol) was dissolved in THF (20 mL) and acetic acid (4 mL), a solution of sodium nitrite (2.08 g, 30.11 mmol) in 4 mL water was added dropwise to reaction mixture at room temperature for 4h. Then the mixture was poured into cool water, and the resulting precipitate was collected by filtration to afford **1a** (1.90 g, 77 % yield).

### 2.3 Synthesis of 3,7-dinitro-10H-phenothiazine (**1b**)

Phenothiazine (2.00 g, 10.04 mmol) was dissolved in THF (20 mL) and acetic acid (4 mL), a solution of sodium nitrite (4.16 g, 60.22 mmol) in 4 mL water was added dropwise to reaction mixture at 50 °C for 12h. Then the mixture was poured into cool water, and the resulting precipitate was collected by filtration to afford **1b** (2.3 g, 79 % yield).

### 2.4 Synthesis of 10-(6-bromohexyl)-3-nitro-10H-phenothiazine (**2a**)

To a solution of **1a** (200.00 mg, 0.82 mmol) and 1,6-Dibromohexane (399.51 mg, 1.64 mmol) in DMF (2 mL) were added NaH (98 mg, 4.09 mmol) in an ice water bath under N_2_ atmosphere. After 0.5 h, the reaction mixture was hated to 100 °C for another 12h. The reaction was quenched by adding 50 mL of cold water, the mixture was extracted with ethyl acetate for three times. Removing the solvent and purifying by chromatography on silica gel to obtain compound **2a** (250 mg, 45 %). ^1^H NMR (400 MHz, DMSO-*d*_6_) *δ* 8.05 (dd, *J* = 9.1, 2.7 Hz, 1H), 7.95 (d, *J* = 2.7 Hz, 1H), 7.26 (ddd, *J* = 8.2, 7.2, 1.6 Hz, 1H), 7.22–7.09 (m, 3H), 7.04 (td, *J* = 7.4, 1.1 Hz, 1H), 3.97 (t, *J* = 7.0 Hz, 2H), 3.49 (t, *J* = 6.7 Hz, 2H), 1.72 (ddt, *J* = 32.0, 13.5, 6.6 Hz, 4H), 1.40 (q, *J* = 3.2 Hz, 4H). ^13^C NMR (101 MHz, DMSO) *δ* 151.20, 142.86, 142.17, 128.71, 127.84, 124.60, 124.52, 124.20, 122.52, 122.39, 117.30, 115.62, 47.57, 35.46, 32.56, 27.48, 26.27, 25.51. MALDI-TOF Mass (*m*/*z*): Calcd for: C18H19BrN2O2S ([M+H]^+^): calcd for 407.04, found 407.73.

### 2.5 Synthesis of 10-(6-bromohexyl)-3,7-dinitro-10H-phenothiazine (**2b**)

The synthetic procedure is similar to **2a** (64 % yield). ^1^H NMR (400 MHz, Chloroform-d) *δ* 8.09 (dd, J = 9.0, 2.6 Hz, 2H), 7.99 (d, J = 2.6 Hz, 2H), 6.94 (d, J = 9.0 Hz, 2H), 3.99 (dd, J = 7.9, 6.4 Hz, 2H), 3.41 (t, J = 6.6 Hz, 2H), 1.87 (ddt, J = 8.7, 5.3, 2.8 Hz, 4H), 1.51 (p, J = 3.5 Hz, 4H). ^13^C NMR (101 MHz, DMSO-*d*_6_) *δ* 151.20, 142.86, 142.17, 128.71, 127.84, 124.60, 124.52, 124.20, 122.52, 122.39, 117.30, 115.62, 47.57, 35.46, 32.56, 27.48, 26.27, 25.51 MALDI-TOF Mass (*m*/*z*): Calcd for: C18H19BrN3O4S (M+2H]^+^): calcd for 453.02, found 453.88.

### 2.6 Synthesis of tert-butyl 2-(3-nitro-10H-phenothiazin-10-yl)acetate (**3a**)

Compound **1a** (1.00 g, 4.09 mmol), t-Butyl bromoacetate (958.24 mg, 4.91 mmol) and tetra-n-butylammonium bromide were dissolved in DCM/acetone (10 mL/10 mL), followed by addition of aqueous sodium hydroxide (10 g/10 mL H_2_O). Then the mixture was stirred for 24 h at room temperature. The solution was extracted with DCM and organic layer was dried by anhydrous sodium sulphate. The crude product was purified by column chromatography on silica to give compound **3a** (1.08 g, 74 %). ^1^H NMR (400 MHz, DMSO-*d*_6_) *δ* 8.04 (dd, J = 9.1, 2.7 Hz, 1H), 7.95 (d, J = 2.7 Hz, 1H), 7.25–7.14 (m, 2H), 7.05 (td, J = 7.5, 1.1 Hz, 1H), 6.81 (d, J = 9.1 Hz, 1H), 6.75 (dd, J = 8.3, 1.2 Hz, 1H), 4.70 (s, 2H), 1.47 (s, 9H). ^13^C NMR (101 MHz, DMSO) *δ* 168.22, 150.25, 142.60, 142.38, 128.61, 127.39, 124.84, 124.34, 122.83, 122.12, 120.81, 116.27, 115.05, 82.55, 55.38, 51.19, 28.11. MALDI-TOF Mass (*m*/*z*): Calcd for: C18H18N2O4S ([M+H]^+^): calcd for 407.04, found 407.73.

### 2.7 Synthesis of tert-butyl 2-(3,7-dinitro-10H-phenothiazin-10-yl)acetate (**3b**)

The synthetic procedure is similar to **3a** (70 % yield). 1H NMR (400 MHz, DMSO-*d*_6_) *δ* 8.09 (dd, J = 9.1, 2.7 Hz, 2H), 8.03 (d, J = 2.7 Hz, 2H), 6.91 (d, J = 9.1 Hz, 2H), 4.82 (s, 2H), 1.47 (s, 9H). 13C NMR (101 MHz, DMSO) *δ* 167.67, 148.45, 143.77, 124.66, 122.53, 122.30, 116.21, 83.02, 51.48, 37.98, 30.05, 29.27, 28.19, 28.10. MALDI-TOF Mass (*m*/*z*): Calcd for: C18H18N3O6S ([M − H]^+^): calcd for 402.08, found 402.79.

### 2.8 Synthesis of 2-(3-nitro-10H-phenothiazin-10-yl)acetic acid (**4a**)

Compound **3a** (1.00 g, 2.79 mmol) was dissolved in DCM (20 mL) followed by addition of trifluoroacetic acid (5 mL). The mixture was heated at 60 °C for 5 h. The solvent was washed by water for three times. Dried and removed the organic solvent, the compound **4a** were obtained without further purification (0.61 mg, 71 %). ^1^H NMR (400 MHz, DMSO-*d*_6_) *δ* 8.03 (dd, J = 9.1, 2.7 Hz, 1H), 7.94 (d, J = 2.7 Hz, 1H), 7.27–7.20 (m, 1H), 7.20–7.13 (m, 1H), 7.04 (t, J = 7.4 Hz, 1H), 6.84 (d, J = 9.2 Hz, 1H), 6.77 (d, J = 8.1 Hz, 1H), 4.70 (s, 2H). ^13^C NMR (101 MHz, DMSO-*d*_6_) *δ* 170.58, 150.24, 142.53, 142.38, 128.64, 127.34, 124.78, 124.38, 122.58, 122.06, 120.56, 116.34, 115.13, 50.55.

### 2.9 Synthesis of 2-(3,7-dinitro-10H-phenothiazin-10-yl)acetic acid (**4b**)

The synthetic procedure is similar to **4a.** (66 % yield). ^1^H NMR (400 MHz, DMSO-*d*_6_) *δ* 8.08 (d, *J* = 2.7 Hz, 1H), 8.06 (d, *J* = 2.7 Hz, 1H), 8.01 (d, *J* = 2.7 Hz, 2H), 6.93 (d, *J* = 9.2 Hz, 2H), 4.81 (s, 2H). ^13^C NMR (101 MHz, DMSO-*d*_6_) *δ* 170.03, 148.44, 143.70, 124.68, 122.44, 122.07, 116.30, 50.96.

### 2.10 Synthesis of **PTZ-mito**

Compound **2a** (100 mg, 0.25 mmol) and triphenylphosphine (77.27 mg,0.29 mmol) were dissolved in DMF (3 mL) and stirred at 100 °C for 10 h. The mixture was extracted three times with ethyl acetate, dried organic layer with anhydrous sodium sulphate, and solvent was removed by evaporation. The crude product was purified by column chromatography on silica to obtain **PTZ-Mito** (144 mg, 82 %). ^1^H NMR (400 MHz, Methanol-*d*_4_) *δ* 8.04 (dd, *J* = 9.0, 2.6 Hz, 1H), 7.88 (dtd, *J* = 8.8, 4.1, 2.0 Hz, 4H), 7.80–7.68 (m, 12H), 7.22 (ddd, *J* = 8.6, 7.3, 1.6 Hz, 1H), 7.12–6.98 (m, 4H), 4.01 (t, *J* = 6.5 Hz, 2H), 1.74 (p, *J* = 6.7 Hz, 2H), 1.65–1.49 (m, 6H), 1.33 (d, *J* = 6.5 Hz, 2H). ^13^C NMR (101 MHz, MeOD) *δ* 151.34, 143.15, 142.39, 134.91, 133.39, 133.29, 130.19, 130.07, 129.45, 127.84, 127.18, 125.39, 123.88, 123.43, 123.33, 121.90, 118.89, 118.03, 116.61, 114.77, 48.23, 48.02, 47.81, 47.60, 47.38, 47.17, 46.96, 29.37, 29.21, 28.93, 26.70, 25.60, 24.95, 21.80, 21.36, 20.85. MALDI-TOF Mass (*m*/*z*): Calcd for: C36H34N2O2PS ([M]^+^): calcd for 589.21, found 589.90.

### 2.11 Synthesis of **PTZ2N-Mito**

The synthetic procedure is similar to **PTZ-Mito**. (83 % yield). ^1^H NMR (400 MHz, Methanol-d4) *δ* 8.10 (dd, J = 9.0, 2.6 Hz, 2H), 7.99 (d, J = 2.6 Hz, 2H), 7.90 (td, J = 5.8, 2.9 Hz, 3H), 7.79–7.72 (m, 12H), 7.18 (d, J = 9.1 Hz, 2H), 4.09 (t, J = 6.7 Hz, 2H), 1.77 (q, J = 7.0 Hz, 2H), 1.68–1.53 (m, 6H), 1.34–1.33 (m, 2H). ^13^C NMR (101 MHz, MeOD) *δ* 149.21, 143.62, 134.95, 133.39, 130.20, 130.07, 124.37, 123.77, 122.21, 118.88, 118.03, 116.04, 29.33, 25.57, 25.06, 21.86, 21.41, 20.90, 13.04. MALDI-TOF Mass (*m*/*z*): Calcd for: C36H34N3O4PS ([M+H]^+^): calcd for 635.19, found 635.81.

### 2.12 Synthesis of **PTZ-Lyso**

A mixture of compound **2a** (100 mg, 0.25 mmol) and diethylamine (90 mg, 1.23 mmol) in DCM (3 mL) was stirred at room temperature for overnight. The solution was washed three times with water, and organic solvent was removed by evaporation. Compound **PTZ-Lyso** was obtained by column chromatography on silica (0.74 mg, 73 %).^1^H NMR (400 MHz, Chloroform-*d*) *δ* 8.06 (dd, *J* = 9.0, 2.6 Hz, 1H), 7.99 (d, *J* = 2.6 Hz, 1H), 7.24 (t, *J* = 7.9 Hz, 1H), 7.15 (dd, *J* = 7.7, 1.6 Hz, 1H), 7.04 (t, *J* = 7.0 Hz, 1H), 6.93 (d, *J* = 8.1 Hz, 1H), 6.87 (d, *J* = 9.0 Hz, 1H), 3.96 (d, *J* = 8.3 Hz, 2H), 3.18–3.02 (m, 4H), 2.94 (dt, *J* = 11.9, 5.1 Hz, 2H), 1.90–1.74 (m, 4H), 1.54–1.47 (m, 2H), 1.38 (q, *J* = 7.3, 6.7 Hz, 8H). ^13^C NMR (101 MHz, CDCl_3_) *δ* 151.12, 142.94, 142.42, 128.00, 127.74, 125.41, 124.18, 123.80, 123.37, 122.74, 116.35, 114.30, 51.73, 47.75, 46.50, 26.57, 26.41, 26.22, 24.34, 9.30. MALDI-TOF Mass (*m*/*z*): Calcd for: C22H29N3O2S ([M]): calcd for 399.20, found 399.82.

### 2.13 Synthesis of **PTZ2N-Lyso**

The synthetic procedure is similar to **PTZ2N-Lyso**. (64 % yield). ^1^H NMR (400 MHz, DMSO-*d*_6_) *δ* 8.10 (dd, *J* = 9.0, 2.7 Hz, 2H), 8.04 (d, *J* = 2.6 Hz, 2H), 7.29 (d, *J* = 9.1 Hz, 2H), 4.06 (t, *J* = 7.1 Hz, 2H), 3.05 (s, 4H), 2.94 (s, 2H), 1.71 (t, *J* = 7.5 Hz, 2H), 1.56 (s, 2H), 1.42 (d, *J* = 6.8 Hz, 2H), 1.32 (d, *J* = 7.5 Hz, 2H), 1.15 (s, 6H). ^13^C NMR (101 MHz, DMSO) *δ* 149.14, 143.46, 124.79, 123.59, 122.93, 117.04, 48.26, 46.58, 26.14, 25.87. MALDI-TOF Mass (*m*/*z*): Calcd for: C22H29N4O4S ([M]): calcd for 444.18, found 444.40.

### 2.14 Synthesis of **PTZ-ER**

Compound **4a** (150 mg, 0.50 mmol) and HATU (283 mg, 0.74 mmol) were dissolved in DMF (5 mL) under N_2_ atmosphere. After 15 min stirring under room temperature, a mixture of N-(2-aminoethyl)-4-methylbenzenesulfonamide (127 mg, 0.60 mmol) and DIEA (128 mg, 0.99 mmol) in DMF (1 mL) were added to the mixture for another 6 h. The mixture was extracted three times with ethyl acetate, dried and removed the organic solvent. The crude product was purified by column chromatography on silica to obtain **PTZ-ER** (190 mg, 76 %). ^1^H NMR (400 MHz, Chloroform-d) *δ* 8.03–7.97 (m, 2H), 7.74–7.69 (m, 2H), 7.30 (d, J = 8.0 Hz, 2H), 7.22–7.14 (m, 2H), 7.02 (td, J = 7.5, 1.1 Hz, 1H), 6.88–6.78 (m, 2H), 6.34 (t, J = 6.0 Hz, 1H), 4.60 (t, J = 6.3 Hz, 1H), 4.52 (s, 2H), 3.20 (q, J = 6.6 Hz, 2H), 2.60 (q, J = 6.7 Hz, 2H), 1.35 (s, 3H). ^13^C NMR (101 MHz, DMSO-*d*_6_) *δ* 167.93, 150.68, 143.20, 142.71, 142.35, 137.93, 130.18, 128.60, 127.00, 124.66, 124.34, 122.48, 121.81, 120.51, 116.65, 115.27, 51.99, 42.41, 39.17, 38.54, 21.43, 19.12. MALDI-TOF Mass (*m*/*z*): Calcd for: C23H22N4O5S2 ([M]): calcd for 498.10, found 498.41.

### 2.15 Synthesis of **PTZ2N-ER**

The synthetic procedure is similar to **PTZ-ER**. (64 % yield). ^1^H NMR (400 MHz, DMSO-*d*_6_) *δ* 8.46 (t, J = 5.8 Hz, 1H), 7.80 (dd, J = 9.0, 2.6 Hz, 2H), 7.73–7.62 (m, 3H), 7.51 (d, J = 2.6 Hz, 2H), 7.40 (d, J = 8.0 Hz, 2H), 6.79 (d, J = 9.0 Hz, 2H), 4.43 (s, 2H), 3.16 (q, J = 6.4 Hz, 2H), 2.78 (q, J = 6.5 Hz, 2H), 2.38 (s, 3H). ^13^C NMR (101 MHz, DMSO) *δ* 167.26, 148.86, 143.57, 143.23, 137.91, 130.19, 127.00, 124.65, 122.21, 121.98, 116.46, 52.32, 42.40, 39.24, 21.43. MALDI-TOF Mass (*m*/*z*): Calcd for: C23H22N4O5S2 ([M+H]^+^): calcd for 543.09, found 544.06.

### 2.16 Synthesis of **PTZ-memb**

Compound **4a** (100 mg, 0.33 mmol) and HATU (151 mg, 0.39 mmol) were dissolved in DMF (2 mL) under N_2_ atmosphere. After 15 min stirring under room temperature, a mixture of 3-(dodecylamino)propane-1-sulfonate (111 mg, 0.36 mmol) and DIEA (85 mg, 0.66 mmol) in DMF (1 mL) were added to the mixture for another 6 h. The mixture was extracted three times with ethyl acetate, dried and removed the organic solvent. The crude product was purified by column chromatography on silica to obtain **PTZ-Memb** (105 mg, 54 %). ^1^H NMR (400 MHz, Methanol-d4) *δ* 8.00 (ddd, J = 13.2, 9.1, 2.7 Hz, 1H), 7.91 (t, J = 2.3 Hz, 1H), 7.22–7.12 (m, 1H), 7.08 (ddd, J = 7.6, 2.8, 1.6 Hz, 1H), 7.00 (tdd, J = 7.5, 3.4, 1.1 Hz, 1H), 6.72–6.62 (m, 2H), 4.92 (s, 2H), 3.83–3.66 (m, 3H), 3.61 (t, J = 7.3 Hz, 1H), 3.52 (dt, J = 17.4, 7.7 Hz, 2H), 3.24 (q, J = 7.4 Hz, 2H), 2.98–2.84 (m, 2H), 2.30–2.06 (m, 2H), 1.80 (s, 1H), 1.68 (d, J = 8.8 Hz, 1H), 1.43 (d, J = 4.6 Hz, 2H), 1.38 (dd, J = 6.7, 3.4 Hz, 12H), 0.98–0.85 (m, 3H). ^13^C NMR (101 MHz, DMSO) *δ* 166.37, 150.84, 142.75, 142.18, 128.36, 127.03, 124.50, 124.28, 124.10, 122.27, 121.73, 120.45, 116.66, 115.43, 54.07, 48.44, 45.68, 45.24, 42.32, 31.75, 29.47, 29.18, 27.61, 26.87, 25.31, 22.56, 18.54, 17.19, 14.42, 12.95. MALDI-TOF Mass (*m*/*z*): Calcd for: C29H40N3O6S2 ([M − H]^+^): calcd for 589.24, found 589.30.

### 2.17 Synthesis of **PTZ2N-Memb**

The synthetic procedure is similar to **PTZ-Memb**. (41 % yield). ^1^H NMR (400 MHz, Methanol-*d*_4_) *δ* 8.04 (ddd, *J* = 11.8, 9.1, 2.6 Hz, 2H), 7.96 (dd, *J* = 2.7, 1.4 Hz, 2H), 6.75 (dd, *J* = 9.1, 2.5 Hz, 2H), 5.02 (s, 2H), 3.77–3.68 (m, 1H), 3.62 (t, *J* = 7.2 Hz, 1H), 3.52 (dt, *J* = 13.3, 7.8 Hz, 2H), 3.20 (t, *J* = 7.2 Hz, 1H), 3.05–2.86 (m, 4H), 2.31–2.08 (m, 3H), 1.81 (s, 1H), 1.68 (d, *J* = 8.2 Hz, 2H), 1.50–1.32 (m, 14H), 0.93–0.89 (m, 3H). ^13^C NMR (101 MHz, MeOD) *δ* 166.79, 166.77, 148.71, 148.67, 143.72, 143.66, 123.61, 123.50, 122.52, 122.43, 121.43, 121.40, 115.42, 51.27, 51.11, 48.23, 48.02, 47.81, 47.60, 47.38, 47.17, 46.96, 46.78, 45.70, 45.52, 44.89, 31.66, 29.47, 29.42, 29.38, 29.35, 29.33, 29.29, 29.23, 29.12, 29.08, 29.05, 28.80, 28.41, 27.23, 26.69, 26.66, 26.10, 25.93, 24.07, 22.90, 22.33, 21.58, 13.03. MALDI-TOF Mass (*m*/*z*): Calcd for: C29H40N4O8S2 ([M]^-^): calcd for 635.22, found 635.43.

### 2.18 UV/vis absorption and fluorescence spectra

The phenoxazine/phenothiazine-based probes were dissolved in dimethyl sulfoxide (DMSO) to form a 1 mM solution, which were diluted to a concentration of 20 μM in different solvent (MeOH, THF, Tol and Acetone). The UV/vis and fluorescence spectra were obtained in these solutions.

### 2.19 Cell culture conditions

HeLa cells were incubated under 5.0 % CO_2_ and 21.0 % O_2_ at 37 °C in a humidified atmosphere in Dulbecco's Modified Eagle Medium (DMEM, Gibco BRL) with 10 % (v/v) fetal bovine serum (FBS, Gibco BRL) and 100 μg/mL streptomycin and penicillin (Gibco BRL).

### 2.20 Cell viability assay

After 90 % confluence, HeLa cells were cultured into 96-well plates (100 μL, 5000 cells/well), respectively. After incubating for 12 h, cells were treated with different concentrations of phenoxazine/phenothiazine-based probes (1, 2, 4, 8 μM) for 24 h. Then, counting kit-8 stock solution (10 μL) was added to each well for incubating 4 h to form a water-soluble formazan dye. At the end of incubation, the absorbance was measured at 450 nm on a microplate reader after shaking for 5 min (PerkinElmer Victor X5, PE).

### 2.21 Confocal microscopy imaging in live cell

For fluorescence imaging in live cells, HeLa cells were cultured with commercial organelle tracker (1 μM) for 40 min. After removing the medium and cells were washed three times with PBS buffer, adding the PTZ series molecules or PXZ series molecules (1 μM, except PTZ-Memb and PTZ2N-Memb (5 μM)) into the cell culture disk for another 40 min. Without removing the excess dyes by PBS buffer, cell imaging was measured with confocal microscopy.

## 3 Results and discussion

### 3.1 Design and synthesis of probes

**Fig. 1:**
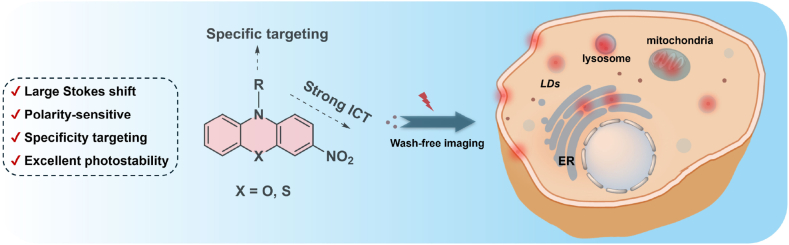
Phenoxazine and phenothiazine-based probes for organelle-targeting wash-free imaging.

As shown in Fig. 1, phenothiazine was employed as electron-donating groups, while strong electron-withdrawing nitro groups were introduced into the aromatic rings to construct donor–acceptor (D–A) structured fluorophores. Additionally, a nitro group was introduced at both sides of the phenothiazine core to generate acceptor–donor–acceptor (A–D–A) type fluorophores. To achieve organelle-targeting functionalization, bromine atoms linked to long alkyl chains were introduced at the nitrogen atom, successfully yielding intermediates 2a and 2b. Furthermore, the introduction of a tert-butoxycarbonyl (Boc) group at the nitrogen atom led to the synthesis of intermediates 3a and 3b, which significantly enhanced the probes' affinity for lipid droplets. As shown in Fig. S2, intermediates 2a/b and 3a/b exhibited similar absorption intensity in THF and methanol solvent, respectively. While the fluorescence intensity was quite different, all of them showed strong emission in non-polar solvent THF, and almost no emission in polar solvent methanol. That proved phenothiazine-based fluorophore have obvious solvatochromism behavior.

**Fig. 2:**
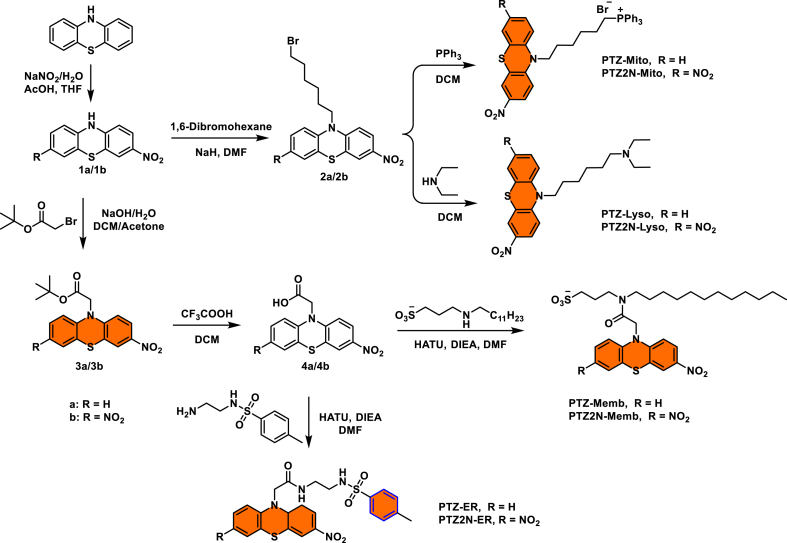
Synthetic route for PTZ/PTZ2N-Based probes.

Additionally, numerous studies have demonstrated that the hydrophilic–hydrophobic balance of a probe plays a critical role in labeling different subcellular organelles. In general, probes with high lipophilicity can readily cross the plasma membrane and subsequently accumulate in specific organelles, such as lysosomes, mitochondria, the endoplasmic reticulum, and lipid droplets [56,57]. In contrast, plasma membrane-targeting probes typically adopt an amphiphilic design, in which the hydrophobic moieties (e.g., long alkyl chains) enable insertion and retention within the lipid bilayer, while the hydrophilic groups (e.g., cationic or anionic substituents) remain exposed at the aqueous interface to ensure membrane localization [58,59]. Here, a series of novel polarity-sensitive and organelle-specific fluorescent probes were developed by conjugating organelle-targeting moieties to a phenothiazine fluorophore scaffold. As shown in Fig. 2, an alkyl sulfonate bearing a dodecyl chain was linked to the fluorophore, yielding the plasma membrane-targeting probes PTZ-Memb and PTZ2N-Memb. Given the reversible protonation of diethylamine at physiological pH, it was used for the construction of lysosome-targeting probes (PTZ-Lyso and PTZ2N-Lyso). Mitochondria-targeting probes (PTZ-Mito and PTZ2N-Mito) incorporated a triphenylphosphonium (TPP) group, enabling accumulation in the mitochondrial matrix via electrostatic interactions with the negatively charged mitochondrial membrane. Endoplasmic reticulum (ER)-targeting probes (PTZ-ER and PTZ2N-ER) functionalized with a p-toluenesulfonamide moiety. The introduction of the Boc group for forming PTZ-Lipid and PTZ2N-Lipid, which not only improved fluorophore solubility but also enhanced affinity for lipid droplets [[60], [61], [62]].

### 3.2 Photophysical properties of phenothiazine-based probes

**Fig. 3:**
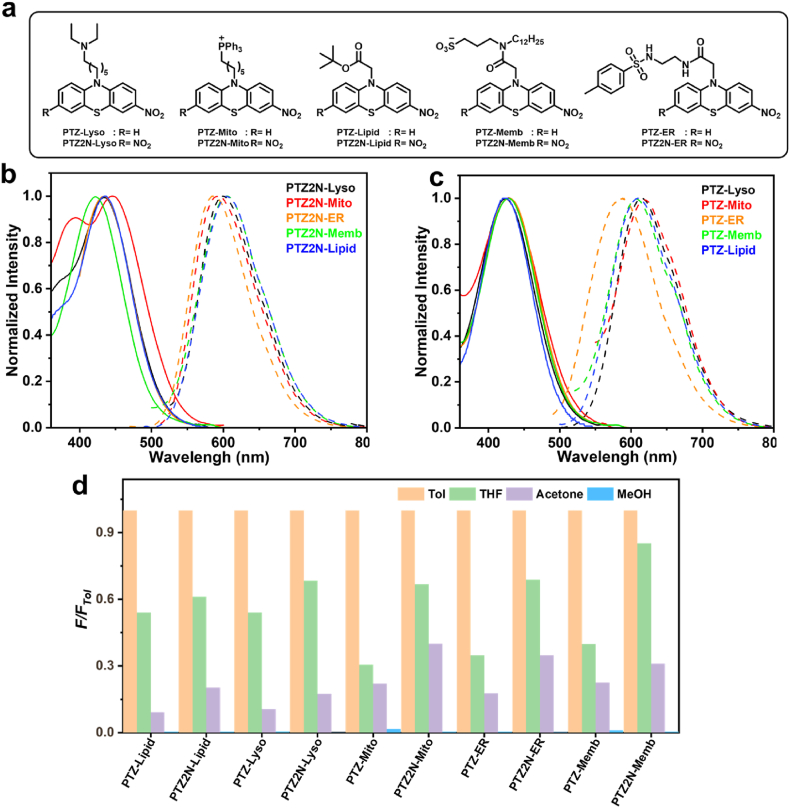
a) Chemical structures of phenothiazine-based organelle targeting probes. b) Normalized absorbance and photoluminescence spectra of PTZ2N-based probes/PTZ-based probes(c). d) Relative fluorescence intensity ratios of phenothiazine-based probes in THF, acetone, and MeOH, compared to their intensities in Tol at the maximum emission peak.

To investigate the photophysical properties of the phenothiazine-based molecules, their UV–Vis absorption and photoluminescence spectra were recorded in tetrahydrofuran (THF). As shown in Fig. 3, the absorption spectra of PTZ-Lyso, PTZ-Mito, PTZ-Memb, PTZ-ER, and PTZ-Lipid exhibit similarities, with maximum absorption and emission peaks at 424/613 nm, 428/619 nm, 427/607 nm, 429/588 nm, and 423/610 nm, respectively. For the PTZ2N series, except for PTZ2N-Memb, which exhibited a slight blue shift, their maximum absorption peaks were red-shifted compared to their PTZ counterparts, with absorption maxima at 435 nm, 446 nm, 422 nm, 433 nm, and 435 nm for PTZ2N-Lyso, PTZ2N-Mito, PTZ2N-Memb, PTZ2N-ER, and PTZ2N-Lipid, respectively. However, the PTZ2N series generally exhibited blue-shifted emission peaks compared to the PTZ series, with emission maxima at 600 nm, 592 nm, 605 nm, 586 nm, and 606 nm, respectively. The blue-shift phenomenon of PTZ2N series may be attributed to the competing effects of the nitro groups located on opposite sides of the phenothiazine in the excited state, leading to a dispersion of the intramolecular charge transfer (ICT) effect between the donor (phenothiazine) and acceptor (nitro) groups [63,64]. Additionally, all molecules exhibited large Stokes shifts ranging from 146 nm to 191 nm in THF (see Table 1 for detailed data).

**Table 1:** Photophysical properties for phenoxazine/phenothiazine-based probes.

Compound	λ_abs__(nm)_^a^	λ_emi (nm)_^a^	Δλ _(nm)_^b^	φ^c^	τ_s_ (_ns_)^d^	*k*_r_ (_s_^−1^)	*k*_nr_ (_s_^−1^)
**PTZ-Lipid**	**423**	**610**	**187**	**0.13**	**2.14**	**5.99×10^7^**	**4.07×10^8^**
**PTZ2N-Lipid**	**435**	**606**	**171**	**0.24**	**7.81**	**3.01×10^7^**	**9.79×10^7^**
**PXZ-Lipid**	**436**	**575**	**139**	**0.17**	**2.71**	**6.27×10^7^**	**3.06×10^8^**
**PXZ2N-Lipid**	**446**	**531**	**85**	**0.68**	**6.34**	**1.08×10^8^**	**5.01×10^7^**
**PTZ-Lyso**	**424**	**613**	**189**	**0.13**	**1.93**	**6.66×10^7^**	**4.52×10^8^**
**PTZ2N-Lyso**	**435**	**600**	**165**	**0.24**	**7.11**	**3.34×10^7^**	**1.07×10^8^**
**PXZ-Lyso**	**454**	**585**	**131**	**0.19**	**2.34**	**7.95×10^7^**	**3.48×10^8^**
**PTZ-Mito**	**428**	**619**	**191**	**0.14**	**2.11**	**6.62×10^7^**	**4.08×10^8^**
**PTZ2N-Mito**	**446**	**592**	**146**	**0.24**	**1.96**	**1.21×10^8^**	**3.89×10^8^**
**PXZ-Mito**	**458**	**589**	**131**	**0.19**	**0.79**	**2.36×10^8^**	**1.03×10^8^**
**PTZ-ER**	**429**	**588**	**159**	**0.16**	**4.51**	**3.61×10^7^**	**1.86×10^8^**
**PTZ2N-ER**	**433**	**586**	**153**	**0.18**	**5.86**	**3.21×10^7^**	**1.39×10^8^**
**PXZ-ER**	**443**	**576**	**133**	**0.18**	**2.42**	**7.37×10^7^**	**3.40×10^8^**
**PXZ2N-ER**	**453**	**535**	**82**	**0.58**	**5.81**	**1.01×10^8^**	**7.16×10^7^**
**PTZ-Memb**	**427**	**607**	**180**	**0.14**	**2.08**	**6.51×10^7^**	**4.16×10^8^**
**PTZ2N-Memb**	**422**	**605**	**183**	**0.19**	**2.76**	**6.84×10^7^**	**2.94×10^8^**

a the maximum absorption peak and the maximum emission peak of probes, measure in Tetrahydrofuran.

b Stokes shift = λ_emi_ – λ_abs_.

c Fluorescence quantum yields, measured by integrated sphere in Toluene.

d Fluorescence lifetime.

To evaluate the environmental sensitivity of the phenothiazine-based molecules, their fluorescence spectra were recorded in solvents of varying polarity, including toluene, THF, acetone, and methanol. As shown in Fig. 3d, all molecules exhibited pronounced solvatochromic effects under identical excitation conditions: fluorescence intensity was highest in low-polarity solvents (toluene) and almost completely quenched in high-polarity solvents (methanol). Additionally, the maximum emission peaks exhibited a red shift as solvent polarity increased (Fig. S3). These observations confirm the ICT nature of these molecules and demonstrate their polarity-sensitive behavior, making them suitable for wash-free live-cell imaging.

The photostability of the dyes was the key factor in monitoring the long-term cell activities. However, some commercial dyes, such as Fluorescein, were shown the poor photostability and limited the application in the specific cell imaging (such as monitoring the formation of lipid droplets). Herein, the photostability of phenothiazine-based probes were tested in DMSO. As shown in Fig. S5, the commercial Fluorescein exhibited obvious decomposition, with its absorption intensity significantly reduced within 30 min under Xe lamp irradiation. In contrast, phenothiazine series molecules showed almost no decompositions under the same conditions, demonstrating phenothiazine series molecules have excellent photostability. Although the phenothiazine was easy to be oxidated within the atmosphere, the nitro group on the aromatic ring decreased the reactive activation, which endowed the molecules wonderful photostability. This indicates that all the probes can be used for monitoring long-term cellular processes.

To assess the cytotoxicity and biocompatibility of phenothiazine-based molecules, a Cell Counting Kit-8 (CCK-8) assay was conducted under dark conditions. HeLa cells were incubated with varying concentrations (1, 2, 4, and 8 μM) of the phenothiazine-based molecules for 24 h, and cell viability was analyzed (Fig. S7). The results demonstrated that high concentrations of mitochondria-targeting probes (PTZ-Mito and PTZ2N-Mito) significantly reduced cell viability. This effect may be attributed to the interaction between the cationic mitochondria-targeting moieties and anionic components of the cell membrane, leading to membrane damage and decreased cell viability. Fortunately, at the working concentration of 1 μM, the viability of cells treated with these probes remained above 80 %, indicating their suitability for live-cell imaging. Apart from the mitochondria-targeting probes, all other molecules exhibited cell viability above 85 % across the tested concentration range (1–8 μM). Furthermore, the phototoxicity of PTZ/PTZ2N series probes were assessed under illumination with a 450 nm LED lamp (Fig. S9). The probes were incubated with HeLa cells in dark for 1 h, followed by 30 min irradiation, the probes were removed and replaced with fresh medium, and the cells were cultured for an additional 8 h in the dark. The results demonstrated that all probes maintained cell viability above 85 % from 1 μM to 8 μM, confirming their excellent biocompatibility and low phototoxicity.

### 3.3 Confocal imaging of organelles in live cells

**Fig. 4:**
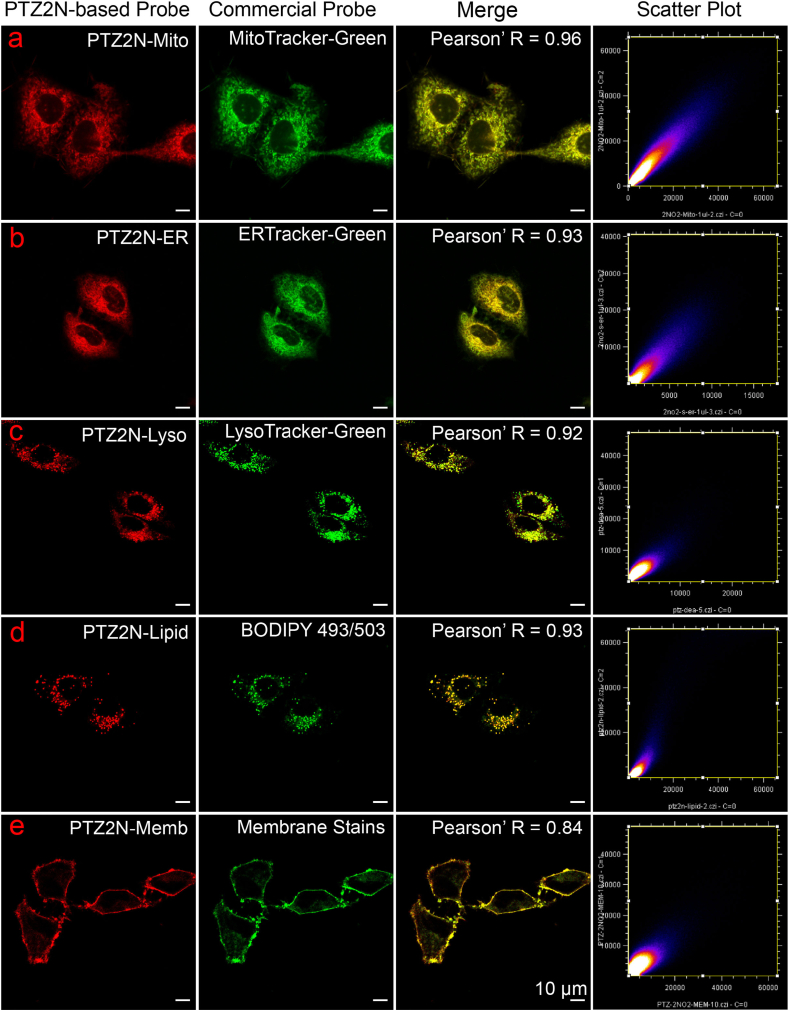
Colocalization of PTZ2N-based probes (in red channel) with commercial organelle probes (in green channel) in live HeLa cells.

phenothiazine-based probes were evaluated for their ability to localize and stain organelles in live cells using the confocal fluorescence microscope. The experimental procedure was as follows: HeLa cells were first incubated with commercial organelle-specific probes for 40 min, followed by three washes with buffer to remove excess dye. Before imaging, PTZ2N probes were directly added to the culture dishes and incubated for another 40 min (5 min for membrane probes) without additional washing. As shown in Fig. 4, PTZ2N probes (red channel) exhibited significant colocalization with commercial organelle-targeting probes in HeLa cells. Pearson's correlation coefficients (Pr) for fluorescence intensity correlation analysis were calculated as follows: PTZ2N-Mito with Mitotracker-Green (0.96), PTZ2N-ER with ERTracker-Green (0.93), PTZ2N-Lyso with Lysotracker-Green (0.92), PTZ2N-Lipid with BODIPY 493/503 (0.90), and PTZ2N-Memb with membrane dye (0.84). As shown in Fig. S11, PTZ2N-based probes exhibited low Pr when compared with non-corresponding commercial organelle probes, further confirming their excellent specificity and targetability toward intended organelles. Additionally, as shown in Fig. S12, studies demonstrated that the red fluorescence channel of phenothiazine-based probes (PTZ-Mito, PTZ-ER, PTZ-Lyso, PTZ-Lipid, and PTZ-Memb) exhibited a high degree of overlap with the green channel of commercial organelle probes, with Pr values ranging from 0.84 to 0.96, confirming their excellent organelle-targeting performance.

To assess the photostability of PTZ2N/PTZ-based probes under continuous laser excitation in live cells, PTZ2N-Mito and PTZ-Mito were selected as representative examples and compared with the commercial probe Mitotracker-Green. As shown in Fig. S13 and S14**,** both PTZ2N-Mito and PTZ-Mito exhibited greater resistance to photobleaching compared to Mitotracker-Green under 488 nm laser irradiation for 30 min in HeLa cells. This enhanced photostability supports the suitability of PTZ2N/PTZ-based probes for long-term cellular imaging applications.

**Fig. 5:**
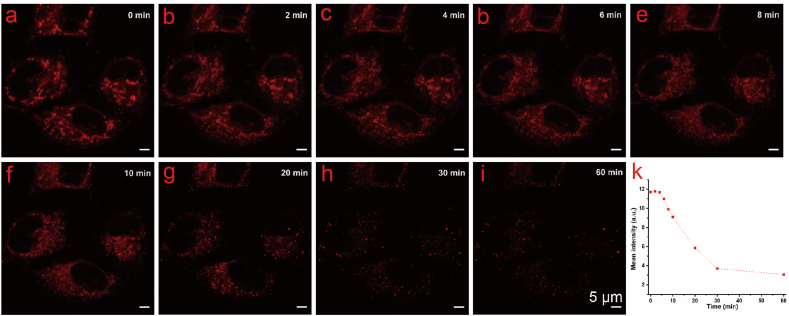
(a–i) Confocal fluorescence imaging of HeLa cells pre-stained with 1 μM PTZ2N-Mito followed by further incubation with 10 μM FCCP for various times. (k) Variation of mean fluorescence intensities in HeLa cells with time during the perfusion of FCCP and PTZ2N-Mito.

To further investigate the mitochondrial targeting mechanism, we employed carbonyl cyanide-p-trifluoromethoxyphenylhydrazone (FCCP), a widely used mitochondrial membrane potential disruptor. HeLa cells were incubated with PTZ2N-Mito for 30 min, followed by treatment with FCCP (10 μM). As shown in Fig. 5, the mean fluorescence intensity of the probe decreased in response to the weakening of mitochondrial membrane potential within 60 min. These results indicate that PTZ2N-Mito localizes to the mitochondrial membrane in a membrane potential-dependent manner.

### 3.4 Photophysical properties of phenoxazine-based probes

**Fig. 6:**
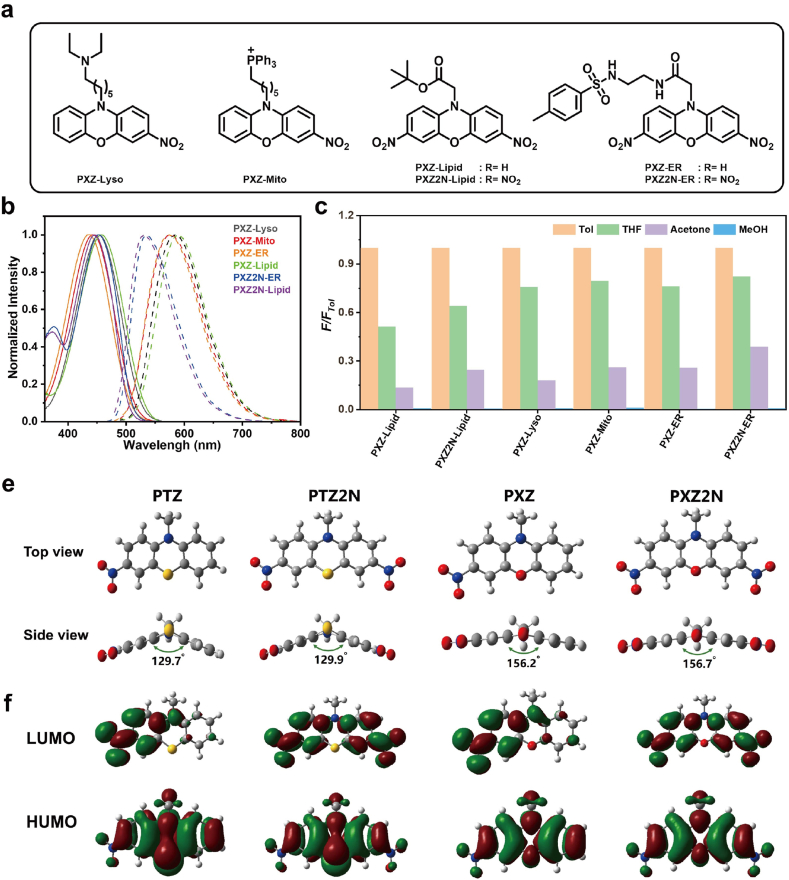
a) Chemical structures of phenoxazine-based organelle targeting probes. b) Normalized absorbance and photoluminescence spectra of phenoxazine-based probes. c) Ratios of relative fluorescence intensities at the maximum emission peak of phenoxazine-based probes in THF, acetone, and MeOH, compared to those in Tol. d) Optimized structure geometry for PTZ/PTZ2N and PXZ/PXZ2N. (targeting groups were replaced with methyl for simplification). f) Frontier molecular orbital distributions (HOMO and LUMO) of PTZ/PTZ2N and PXZ/PXZ2N calculated at the B3LYP/6-31+G(d,p) level of theory.

Encouraged by the promising photophysical and cell imaging performance of phenothiazine-based probes, we next applied this strategy to phenoxazine to synthesize probe PXZ-Lyso, PXZ-Mito, PXZ-ER, PXZ-Lipid, PXZ2N-ER and PXZ2N-Lipid (Fig. 6a). As shown in Fig. 6c and Fig. S4, Phenoxazine-based probes also exhibited apparent polarity-sensitive behavior, fluorescence intensity increased with the decreasing solvent polarity. Furthermore, the UV–Vis absorption and photoluminescence spectra of phenoxazine-based molecules were also measured in THF (Fig. 6b). The maximum absorption/emission peaks for PXZ-Lyso, PXZ-Mito, PXZ-ER, and PXZ-Lipid were observed at 454/585 nm, 458/589 nm, 443/576 nm, and 436/575 nm, respectively. The photophysical properties of dinitro-substituted PXZ2N series were similar to those of PTZ2N, with red-shifted absorption and blue-shifted emission compared to PXZ molecules. The maximum absorption/emission peaks of PXZ2N-ER and PXZ2N-Lipid were recorded at 446/531 nm and 453/535 nm, respectively. Compared to the PTZ derivatives, PXZ derivatives exhibited significantly red-shifted absorption peaks and blue-shifted emission peaks, resulting in a reduced Stokes shift range of 82–139 nm. (see Table 1 for detailed data).

### 3.5 Calculation

To gain deeper insight into the molecular conformations and electronic properties of the PTZ/PTZ2N and PXZ/PXZ2N derivatives, we performed time-dependent density functional theory (TD-DFT) calculations at the b3lyp/6–31+g(d,p) level using Gaussian 09. To simplify the models and reduce computational cost, the organelle-targeting moieties at the nitrogen positions were replaced with methyl groups. As illustrated in Fig. 6e, both PTZ and PTZ2N adopt a characteristic butterfly-like, bent molecular conformation due to the presence of the central sulfur atom. The dihedral angles between the two six-membered aromatic rings of the phenothiazine core were calculated to be 129.7° and 129.9°, respectively. In contrast, PXZ and PXZ2N exhibited more planar structures, with dihedral angles of 156.2° and 156.7°, respectively. This enhanced planarity can be attributed to the oxygen atom at the core, which imposes less steric hindrance and allows for more effective π-conjugation. The simulated frontier molecular orbitals (HOMO and LUMO) of these molecules are also shown in Fig. 6f. For the HOMO orbitals, PXZ and PXZ2N show extensive delocalization across the entire phenoxazine scaffold, indicating efficient π-conjugation. Meanwhile, the HOMOs of PTZ and PTZ2N are mainly localized on the central tricyclic ring, with significant electron density near the sulfur atom—reflecting the strong electron-donating nature and lower electronegativity of sulfur, which tends to confine electron density more locally. As for the LUMO orbitals, all four molecules exhibit similar electronic characteristics: the LUMOs are predominantly localized on the nitro-substituted aromatic ring at the periphery, with negligible contributions from the central heteroatoms (S or O). This spatial separation between the HOMO and LUMO supports the presence of an ICT transition upon excitation, where electron density shifts from the donor core (PTZ or PXZ) to the nitro-accepting moiety. Finally, the calculated HOMO and LUMO energy levels are as follows: 5.70/–2.61 eV for PTZ, −6.11/–3.31 eV for PTZ2N, −5.86/–2.68 eV for PXZ, and −6.38/–3.15 eV for PXZ2N (Fig. S10) These results highlight that the introduction of nitro groups significantly lowers both frontier orbital energy levels, especially the LUMO, thereby enhancing the electron-accepting ability of the molecules and facilitating efficient ICT transitions in the excited state.

### 3.6 Phenoxazine-based probes labeled cell imaging

**Fig. 7:**
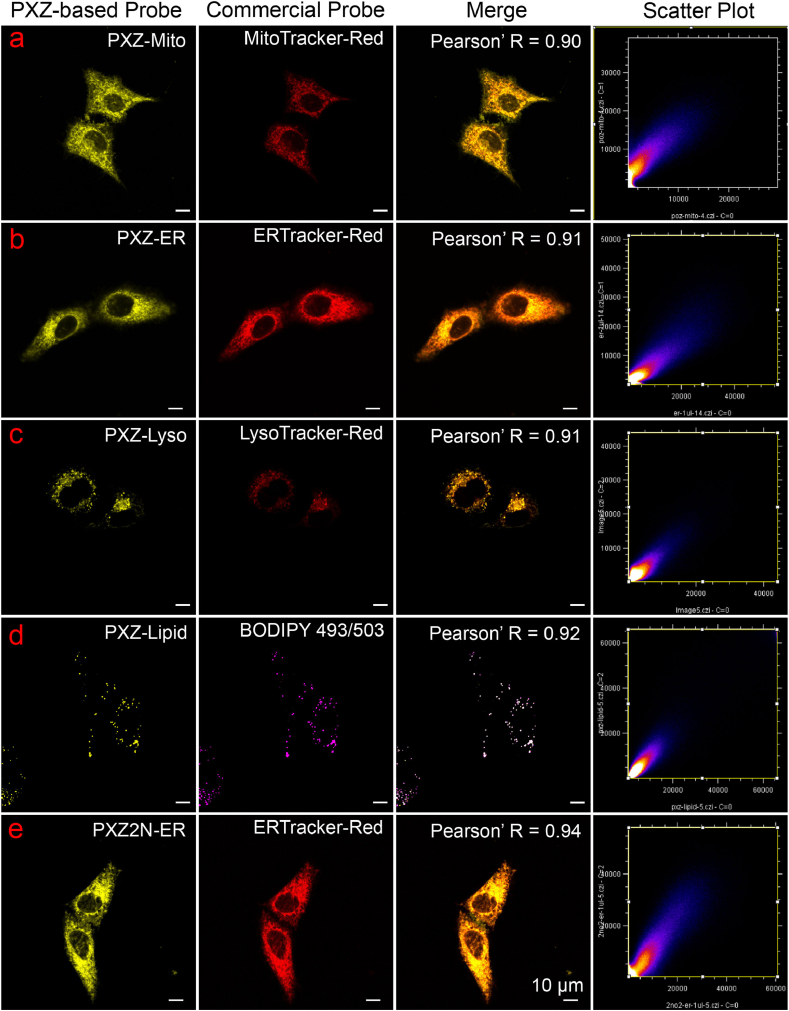
Colocalization of phenoxazine-based probes (in yellow channel) with commercial organelle probes (in red, purple channel for BODIPY 493/503) in live HeLa cells.

**Fig. 8:**
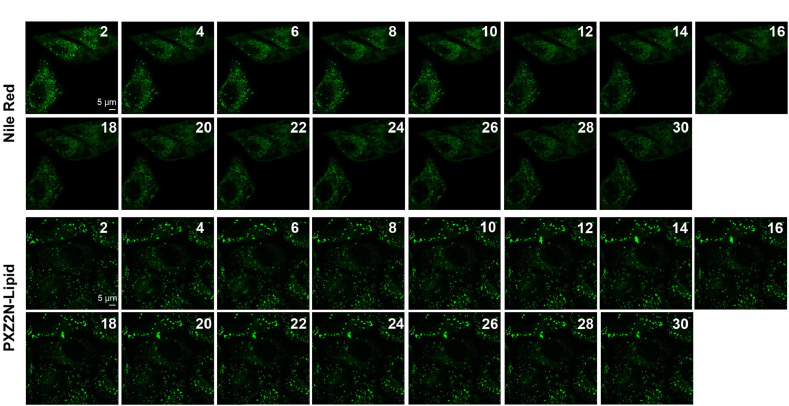
Fluorescence intensity changes of commercial dyes and PXZ2N-Lipid during 30 min of irradiation (λ_ex_ = 488 nm; 60 % laser intensity; Pixel dwell: 1.40 μsec; Scan time:1 min 58 s; speed: 6; Averaging number: 16).

Phenoxazine-based molecules exhibited excellent biocompatibility and low phototoxicity, as demonstrated in Figure S8 and Figure S9c. Therefore, the imaging performance of phenoxazine-based probes were measured through colocalization experiments. After incubation with commercial probes and washing, HeLa cells were imaged with phenoxazine probes under wash-free conditions. As shown in Fig. 7, the results showed strong colocalization between PXZ-Mito and MitoTracker-Red (Pr = 0.90), PXZ-ER and ERTracker-Red (Pr = 0.91), PXZ-Lyso and LysoTracker-Red (Pr = 0.91), PXZ-Lipid and BODIPY 493/503 (Pr = 0.92), and PXZ2N-ER and ERTracker-Red (Pr = 0.94), PXZ2N-Lipid and BODIPY 493/503 (Pr = 0.92, Fig. S15), indicating precise organelle-targeting capabilities. Additionally, PXZ/PXZ2N-based probes exhibited excellent photostability under xenon lamp irradiation for 30 min (Fig. S6). To further assess their performance under continuous laser excitation in live cells, PXZ-Mito and PXZ2N-Lipid were selected and compared with the commercial probes Mitotracker-Green and Nile Red. As demonstrated in Figs. S13, S14, S16, and Figure 8, both PXZ-Mito and PXZ2N-Lipid showed significantly enhanced photostability relative to Mitotracker-Green and Nile Red (see Fig. 8).

**Fig. 9:**
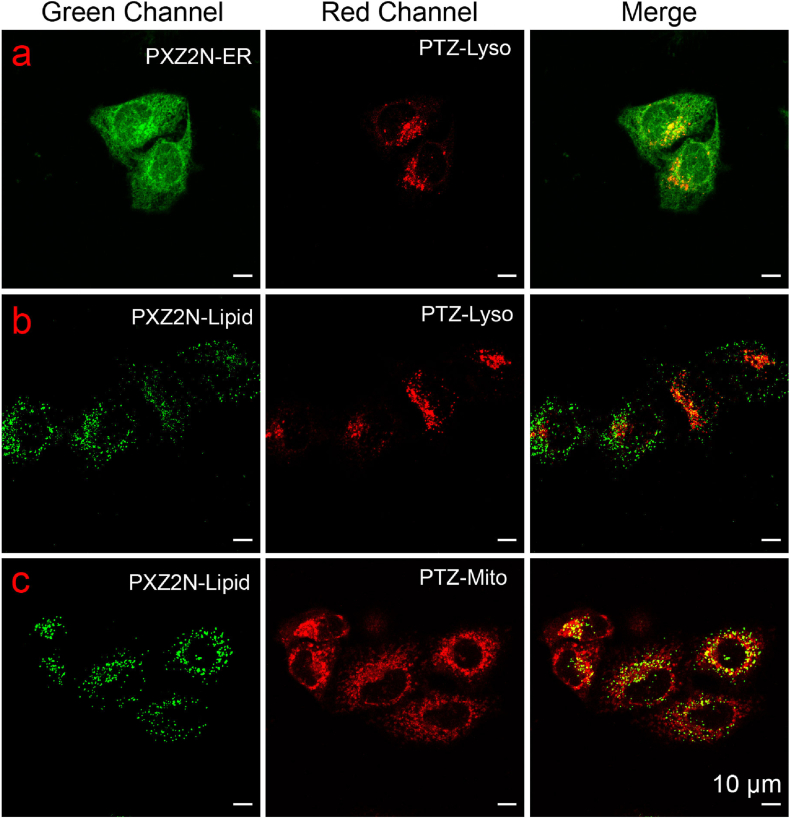
Fluorescent images of live HeLa cells stained with PXZ2N-based probes and PTZ-based probes (λ_ex_ = 488 nm; λ_em_ = 493–567 nm for PXZ2N, λ_em_ = 670–759 nm for PTZ).

Based on the outstanding performance of the phenoxazine-based probes in live-cell imaging, they were further applied to multicolor imaging under wash-free conditions. As shown in Fig. 9, the green channel represents PXZ2N probes, while the red channel represents PTZ probes. The merged images clearly reveal the spatial distribution differences between the endoplasmic reticulum and lysosomes (Fig. 9a). In Fig. 9a/c, the green channel shows lipid droplets labeled by PXZ2N-Lipid, while the red channel shows lysosomes or mitochondria labeled by PTZ-Lyso or PTZ-Mito, respectively. The merged images provide a comprehensive visualization of the morphology and localization of lipid droplets, lysosomes, and mitochondria. The advantage of these probes lies in their ability to emit fluorescence at different wavelengths under the same laser excitation, enabling multicolor imaging without the need for frequent wavelength switching. This significantly enhances imaging efficiency and ensures a high degree of differentiation for organelle signals.

### 3.7 In situ monitoring dynamic changes of LDs in living cells

**Fig. 10:**
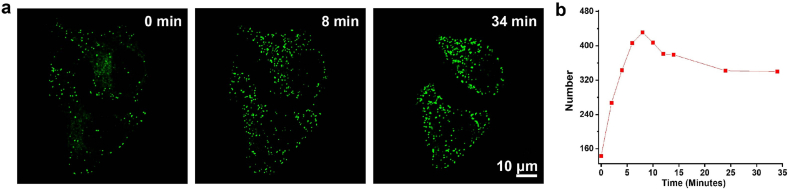
a) In situ monitoring lipid droplets dynamic in oleic acid-treated SKOV3 cells stained with PXZ-Lipid. Scale bar: 10 μM. b) The number of lipid droplets on oleic acid-treated SKOV3 cells at different times (0, 2, 4, 6, 8, 10, 12, 14, 24 and 34 min). c)Quantitative analysis of lipid droplet size and number over time, analyzed using ImageJ.

Lipid droplets (LDs) are dynamic and multifunctional organelles primarily responsible for lipid storage, energy metabolism, and membrane biosynthesis [[65], [66], [67], [68]]. Owing to its excellent photostability, PXZ-Lipid was selected as a fluorescent probe to monitor the dynamic behavior of LDs in SKOV3 cells. To deplete endogenous LDs, SKOV3 cells were cultured in nutrient-deprived medium for three days. Subsequently, the cells were incubated with PXZ-Lipid for 1 h, followed by treatment with oleic acid (OA) to induce LD formation [69]. This protocol enabled continuous tracking of LD dynamics in the same region of the culture dish. Fig. S18 illustrates notable changes in the number and morphology of lipid droplets (LDs) over a 34-min period. Building upon these observations, ImageJ was used for automated quantification of LD number and size, and the results are summarized in Fig. S18c. At the beginning of the observation (0 min), approximately 143 small LDs were detected. Upon OA stimulation, both the number and size of LDs markedly increased, peaking at around 431 LDs at 8 min. Thereafter, the LD count gradually declined and stabilized at approximately 340 by 24 min (Fig. 10b). Interestingly, although the total number of LDs decreased with prolonged OA treatment, the size of LDs increased significantly (Fig. 10a). As shown in Fig. S18c, initial LD sizes were all below 0.32 μm^2^. After 34 min of OA incubation, 61 LDs with areas exceeding 0.32 μm^2^ were observed, with the largest reaching 2.04 μm^2^. This enlargement is likely due to the fusion or aggregation of smaller LDs into larger ones. These results demonstrate that PXZ-Lipid enables high-resolution, real-time tracking of LD formation and morphological evolution, offering a powerful tool for studying dynamic changes of lipid in living cells.

## 4 Conclusion

In conclusion, by introducing organelle-targeting moieties into phenothiazine frameworks and incorporating conjugated nitro groups, we have successfully developed a series of organelle-specific fluorescent probes with large Stokes shifts. These probes exhibit remarkable environmental sensitivity and outstanding performance in wash-free cellular imaging, eliminating the need for tedious washing steps and minimizing sample disturbance. Furthermore, the same molecular design strategy was extended to phenoxazine-based systems, which also demonstrated pronounced environmental responsiveness and excellent imaging capabilities under wash-free conditions. Notably, the PTZ-series and PXZ2N-series probes enable dual-color wash-free imaging within living cells, offering complementary spectral windows and reducing the overlap between imaging channels. This dual-color approach not only enhances imaging contrast but also shortens the exposure time required for data acquisition, thereby improving experimental efficiency and reducing phototoxicity. Additionally, benefiting from the excellent photostability and organelle selectivity of PXZ-Lipid, dynamic monitoring of intracellular lipid droplet fluctuations was successfully achieved. Overall, this study presents a versatile design strategy for constructing environmentally responsive, organelle-specific fluorescent probes, which hold great potential for advanced bioimaging applications and real-time monitoring of subcellular dynamics in living systems.

## CRediT authorship contribution statement

**Zhichao Wang:** Writing – review & editing, Writing – original draft, Methodology. **Jinxiao Lyu:** Methodology. **Yongjie Sun:** Methodology. **Fang Liu:** Methodology. **Lanqing Li:** Supervision. **Shaoping Li:** Supervision. **Xuanjun Zhang:** Writing – review & editing, Supervision, Project administration, Methodology, Funding acquisition.

## Declaration of competing interest

The authors declare the following financial interests/personal relationships which may be considered as potential competing interests: Xuanjun Zhang, Zhichao Wang has patent licensed to CN 118638152 B. If there are other authors, they declare that they have no known competing financial interests or personal relationships that could have appeared to influence the work reported in this paper.

## Data Availability

No data was used for the research described in the article.

## Appendix A Supplementary data

Synthetic procedures of phenoxazine-based probes, NMR spectra (^1^H and ^13^C), MALDI-TOF Mass data, fluorescence imaging protocols, Figures (S1-S18) and Table S1.
