# Supplementary material for: Cell wash-free fluorescent probes based on phenothiazine and phenoxazine with high photostability and large stokes shifts for targeted imaging of subcellular organelles
Source: Mater Today Bio. 2025 Oct 10;35:102399. doi: 10.1016/j.mtbio.2025.102399 (PMC12550198; doi:10.1016/j.mtbio.2025.102399)

**Cell Wash-Free Fluorescent Probes Based on Phenothiazine and Phenoxazine with High Photostability and Large Stokes Shifts for Targeted Imaging of Subcellular Organelles**

Zhichao Wang^1^, Jinxiao Lyu^1^, Yongjie Sun^1^, Fang Liu^1^, Lanqing Li,*^2^ Shaoping Li,*^3^ Xuanjun Zhang*^1,4^

*^1^Faculty of Health Sciences, University of Macau, Taipa, Macau SAR, China*

*^2^School of Pharmacy and Food Engineering, Wuyi University, Jiangmen 529020, PR China*

*^3^State Key Laboratory of Quality Research in Chinese Medicine, Institute of Chinese Medical Sciences, University of Macau, Macao SAR, China*

*^4^MOE Frontiers Science Center for Precision Oncology, University of Macau, Taipa, Macau SAR, China*

**Synthesis**

**Synthesis of 10-(6-bromohexyl)-3-nitro-10H-phenoxazine (2c)**

The synthetic procedure is similar to **2a** (74% yield). ^1^H NMR (400 MHz, Chloroform-d) δ 7.75 (dd, J = 9.0, 2.6 Hz, 1H), 7.44 (d, J = 2.6 Hz, 1H), 6.87 (td, J = 7.7, 1.6 Hz, 1H), 6.79 (td, J = 7.7, 1.5 Hz, 1H), 6.71 – 6.66 (m, 1H), 6.57 (dd, J = 8.0, 1.5 Hz, 1H), 6.43 (d, J = 8.9 Hz, 1H), 3.62 – 3.52 (m, 2H), 3.46 (t, J = 6.6 Hz, 2H), 1.97 – 1.88 (m, 2H), 1.72 (p, J = 7.7 Hz, 2H), 1.60 – 1.54 (m, 2H), 1.48 (dd, J = 10.6, 5.3 Hz, 2H). ^13^C NMR (101 MHz, CDCl_3_) δ 144.36, 144.27, 140.82, 139.68, 130.88, 124.31, 123.14, 121.23, 115.95, 112.26, 110.70, 109.65, 44.25, 33.59, 32.54, 27.83, 26.02, 24.96. MALDI-TOF Mass (m/z): Calcd for: C18H19BrN2O3 ([M+H]^+^): calcd for 491.06, found 391.72.

**Synthesis of tert-butyl 2-(3-nitro-10H-phenoxazin-10-yl)acetate (3c)**

The synthetic procedure is similar to **3a** (72% yield). 1H NMR (400 MHz, Chloroform-d) δ 13.33 (s, 1H), δ 7.79 (dd, J = 8.9, 2.6 Hz, 1H), 7.55 (d, J = 2.5 Hz, 1H), 6.87 (dtd, J = 19.4, 7.5, 1.6 Hz, 2H), 6.76 (dd, J = 7.7, 1.7 Hz, 1H), 6.47 (dd, J = 7.8, 1.6 Hz, 1H), 6.39 (d, J = 8.9 Hz, 1H), 4.22 (s, 2H), 1.51 (s, 9H). ^13^C NMR (101 MHz, Chloroform-d) δ 167.14, 144.85, 144.50, 141.71, 139.95, 131.42, 124.37, 123.58, 120.86, 116.04, 112.39, 110.96, 110.38, 83.43, 47.58, 28.04. MALDI-TOF Mass (m/z): Calcd for: C18H18N2O5 ([M-H]^+^): calcd for 341.12, found 341.53.

**Synthesis of tert-butyl 2-(3,7-dinitro-10H-phenoxazin-10-yl)acetate (3d)**

The synthetic procedure is similar to **3a** (77% yield). 1H NMR (400 MHz, Chloroform-d) δ 7.85 (dd, J = 8.9, 2.5 Hz, 2H), 7.62 (d, J = 2.5 Hz, 2H), 6.51 (d, J = 8.9 Hz, 2H), 4.29 (s, 2H), 1.53 (s, 9H). 13C NMR (101 MHz, CDCl3) δ 166.11, 144.27, 143.13, 137.76, 121.11, 111.58, 111.56, 84.30, 77.35, 77.03, 76.72, 47.74, 28.04. MALDI-TOF Mass (m/z): Calcd for: C18H18N3O7 ([M-C(CH3)3]^+^): calcd for 330.1, found 330.44.

**Synthesis of 2-(3-nitro-10H-phenoxazin-10-yl)acetic acid (4c)**

The synthetic procedure is similar to **4a.** (79% yield). ^1^H NMR (400 MHz, DMSO-*d*_6_) δ 7.77 (dd, *J* = 9.0, 2.6 Hz, 1H), 7.43 (d, *J* = 2.6 Hz, 1H), 6.90 (td, *J* = 7.6, 1.7 Hz, 1H), 6.83 (td, *J* = 7.6, 1.4 Hz, 1H), 6.78 (dd, *J* = 7.8, 1.6 Hz, 1H), 6.74 – 6.66 (m, 2H), 4.44 (s, 2H). ^13^C NMR (101 MHz, DMSO-d6) δ 170.09, 144.03, 143.89, 140.77, 140.74, 131.71, 125.06, 123.66, 121.74, 115.84, 113.90, 112.20, 110.19, 46.56.

**Synthesis of 2-(3,7-dinitro-10H-phenoxazin-10-yl)acetic acid(4d)**

The synthetic procedure is similar to **4a.** (75% yield). ^1^H NMR (400 MHz, DMSO-*d*_6_) δ 7.84 (dd, *J* = 9.0, 2.6 Hz, 2H), 7.53 (d, *J* = 2.6 Hz, 2H), 6.92 (d, *J* = 9.0 Hz, 2H), 4.66 (s, 2H). ^13^C NMR (101 MHz, DMSO) δ 174.24, 169.44, 143.62, 142.48, 138.38, 121.85, 113.65, 110.87, 46.58.

**Synthesis of PXZ-Mito**

The synthetic procedure is similar to **PTZ-Mito**. (80% yield). ^1^H NMR (400 MHz, Chloroform-d) δ 7.90 (dd, J = 12.6, 7.6 Hz, 6H), 7.84 – 7.78 (m, 3H), 7.72 (tt, J = 7.6, 3.3 Hz, 7H), 7.41 (d, J = 2.3 Hz, 1H), 6.86 (t, J = 7.5 Hz, 1H), 6.75 (t, J = 7.6 Hz, 1H), 6.64 (d, J = 7.9 Hz, 2H), 6.61 – 6.55 (m, 1H), 3.99 (s, 2H), 3.63 (s, 2H), 1.81 (s, 2H), 1.60 (s, 4H), 1.27 (s, 2H). ^13^C NMR (101 MHz, Acetone) δ144.03, 139.84, 134.89, 134.86, 134.04, 133.94, 130.30, 130.25, 130.17, 124.63, 122.90, 121.33, 119.46, 118.61, 115.45, 113.32, 111.46, 109.64, 43.64, 24.87, 24.25, 21.89, 21.51, 21.08. MALDI-TOF Mass (m/z): Calcd for: C36H34N2O3P ([M+H]^+^): calcd for 574.23, found 574.10.

**Synthesis of PXZ-Lyso**

The synthetic procedure is similar to **PXZ-Lyso**. (62% yield). ^1^H NMR (400 MHz, Chloroform-*d*) δ 7.77 (dd, *J* = 9.0, 2.6 Hz, 1H), 7.45 (d, *J* = 2.6 Hz, 1H), 6.89 (td, *J* = 7.7, 1.6 Hz, 1H), 6.79 (t, *J* = 7.7 Hz, 1H), 6.69 (dd, *J* = 7.9, 1.6 Hz, 1H), 6.58 (d, *J* = 8.0 Hz, 1H), 6.46 (d, *J* = 9.0 Hz, 1H), 3.58 (t, *J* = 7.9 Hz, 2H), 3.26 – 3.09 (m, 4H), 3.01 (dt, *J* = 10.4, 5.2 Hz, 2H), 1.93 (p, *J* = 7.8, 7.3 Hz, 2H), 1.72 (d, *J* = 7.7 Hz, 2H), 1.53 (dd, *J* = 7.5, 4.0 Hz, 4H), 1.45 (t, *J* = 7.3 Hz, 6H). ^13^C NMR (101 MHz, CDCl_3_) δ 144.36, 144.28, 140.78, 139.72, 130.91, 124.29, 123.10, 121.21, 115.92, 112.28, 110.66, 109.66, 7 66.81, 58.91, 53.72, 44.38, 27.23, 26.78, 26.37, 25.09. MALDI-TOF Mass (m/z): Calcd for: C22H29N3O3 ([M]): calcd for 383.22, found 383.85

**Synthesis of PXZ-ER**

The synthetic procedure is similar to **PTZ-ER**. (71% yield).1H NMR (400 MHz, Chloroform-d) δ 8.00 (dd, J = 8.9, 2.6 Hz, 1H), 7.96 (d, J = 2.6 Hz, 1H), 7.19 – 7.14 (m, 1H), 7.11 (dd, J = 7.7, 1.7 Hz, 1H), 7.03 (td, J = 7.5, 1.1 Hz, 1H), 6.70 – 6.62 (m, 2H), 4.62 (s, 2H), 4.53 – 4.39 (m, 2H), 3.84 – 3.72 (m, 2H), 3.73 – 3.65 (m, 6H), 3.61 – 3.55 (m, 2H), 3.39 (s, 3H). ^13^C NMR (101 MHz, DMSO) δ 167.27, 144.14, 143.95, 143.20, 140.94, 140.79, 137.90, 131.86, 130.17, 126.98, 125.02, 123.69, 121.69, 115.74, 113.88, 112.18, 110.02, 47.71, 42.28, 39.11, 21.43. MALDI-TOF Mass (m/z): Calcd for: C23H22N4O7 ([M-H]) ^+^: calcd for 481.13, found 481.69.

**Synthesis of PXZ2N-ER**

The synthetic procedure is similar to **PTZ-ER**. (61% yield). 1H NMR (400 MHz, DMSO-d6) δ 8.46 (t, J = 5.8 Hz, 1H), 7.80 (dd, J = 9.0, 2.6 Hz, 2H), 7.73 – 7.62 (m, 3H), 7.51 (d, J = 2.6 Hz, 2H), 7.40 (d, J = 8.0 Hz, 2H), 6.79 (d, J = 9.0 Hz, 2H), 4.43 (s, 2H), 3.16 (q, J = 6.4 Hz, 2H), 2.78 (q, J = 6.5 Hz, 2H), 2.38 (s, 3H). ^13^C NMR (101 MHz, DMSO) δ 167.27, 144.14, 143.95, 143.20, 140.94, 140.79, 137.90, 131.86, 130.17, 126.98, 125.02, 123.69, 121.69, 115.74, 113.88, 112.18, 110.02, 47.71, 42.28, 39.11, 21.43. MALDI-TOF Mass (m/z): Calcd for: C23H22N5O8S1 ([M]): calcd for 528.11, found 528.07.

For fluorescence imaging in live cells, HeLa cells were cultured with commercial organelle tracker (1 μM) for 40 minutes. After removing the medium and cells were washed three times with PBS buffer, adding the PTZ series molecules or PXZ series molecules (1 μM, except PTZ Memb and PZT2N-Memb (5μM)) into the cell culture disk for another 40 minutes. Without removing the excess dyes by PBS buffer, cell imaging was measured with confocal microscopy. (PTZ-Lyso and Lysotracker Green: λ_ex_ = 488 nm; λ_em_ = 493-586 nm for Lysotracker Green, λ_em_ = 638-759 nm for PTZ-Lyso. PTZ-Mito and Mitotracker Green: λ_ex_ = 488 nm; λ_em_ = 494-604 nm for Mitotracker Green, λ_em_ = 633-759 nm for PTZ-Mito. PTZ-ER and ERTracker Green: λ_ex_ = 488 nm; λ_em_ = 493-614 nm for ERTracker Green, λ_em_ = 636-759 nm for PTZ-ER. PTZ-Lipid and BODIPY 493/503: λ_ex_ = 488 nm; λ_em_ = 493-614 nm for BODIPY 493/503, λ_em_ = 636-759 nm for PTZ-Lipid. PTZ-Memb and Membrane stains, λ_ex_ = 488 nm; λ_em_ = 493-604 nm for Membrane stains, λ_em_ = 633-759 nm for PTZ-Memb. PTZ2N-Lyso and Lysotracker Green: λ_ex_ = 488 nm; λ_em_ = 493-586 nm for Lysotracker Green, λ_em_ = 638-759 nm for PTZ-Lyso. PTZ2N-Mito and Mitotracker Green: λ_ex_ = 488 nm; λ_em_ = 493-604 nm for Mitotracker Green, λ_em_ = 638-759 nm for PTZ2N-Mito. PTZ2N-ER and ERTracker Green: λ_ex_ = 488 nm; λ_em_ = 493-614 nm for ERTracker Green, λ_em_ = 636-759 nm for PTZ2N-ER. PTZ-Lipid and BODIPY 493/503: λ_ex_ = 488 nm; λ_em_ = 493-550 nm for BODIPY 493/503, λ_em_ = 609-725 nm for PTZ2N-Lipid. PTZ2N-Memb and Membrane stains, λ_ex_ = 488 nm; λ_em_ = 493-634 nm for Membrane stains, λ_em_ = 638-759 nm for PTZ2N-Memb. PXZ-Lyso and Lysotracker Red: λ_ex_ = 561 nm for Lysotracker Red, λ_ex_ = 488 nm for PXZ-Lyso; λ_em_ = 635-759 nm for Lysotracker Red, λ_em_ = 537-603 nm for PXZ-Lyso. PXZ-Mito and Mitotracker Red: λ_ex_ = 543 nm for Mitotracker Red, λ_ex_ = 488 nm for PXZ-Mito; λ_em_ = 619-759 nm for Mitotracker Red, λ_em_ = 493-578 nm for PXZ-Mito. PXZ-ER and ERtracker Red: λ_ex_ = 543 nm for ERtracker Red, λ_ex_ = 488 nm for PXZ-ER; λ_em_ = 617-759 nm for ERtracker Red, λ_em_ = 493-570 nm for PXZ-ER. PXZ-Lipid and BODIPY 493/503: λ_ex_ = 488 nm; λ_em_ = 493-537 nm for BODIPY 493/503, λ_em_ = 609-704 nm for PXZ-Lipid. PXZ2N-ER and ERtracker Red: λ_ex_ = 543 nm for ERtracker Red, λ_ex_ = 488 nm for PXZ2N-ER; λ_em_ = 617-759 nm for ERtracker Red, λ_em_ = 493-570 nm for PXZ2N-ER. PXZ2N-Lipid and BODIPY 493/503: λ_ex_ = 488 nm; λ_em_ = 493-558 nm for BODIPY 493/503, λ_em_ = 595-660 nm for PXZ2N-Lipid.)


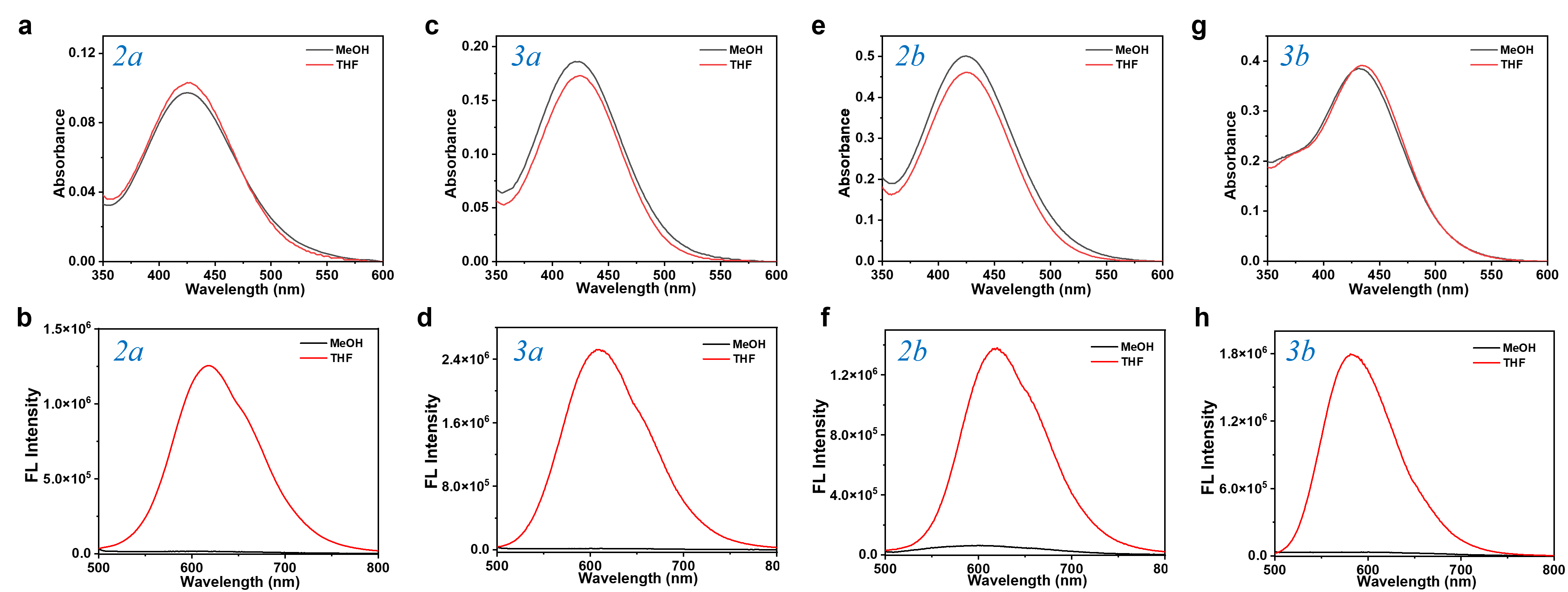


**Figure S1** The absorption spectra and fluorescence emission spectra of 2a/2b and 3a/3b in MeOH and THF


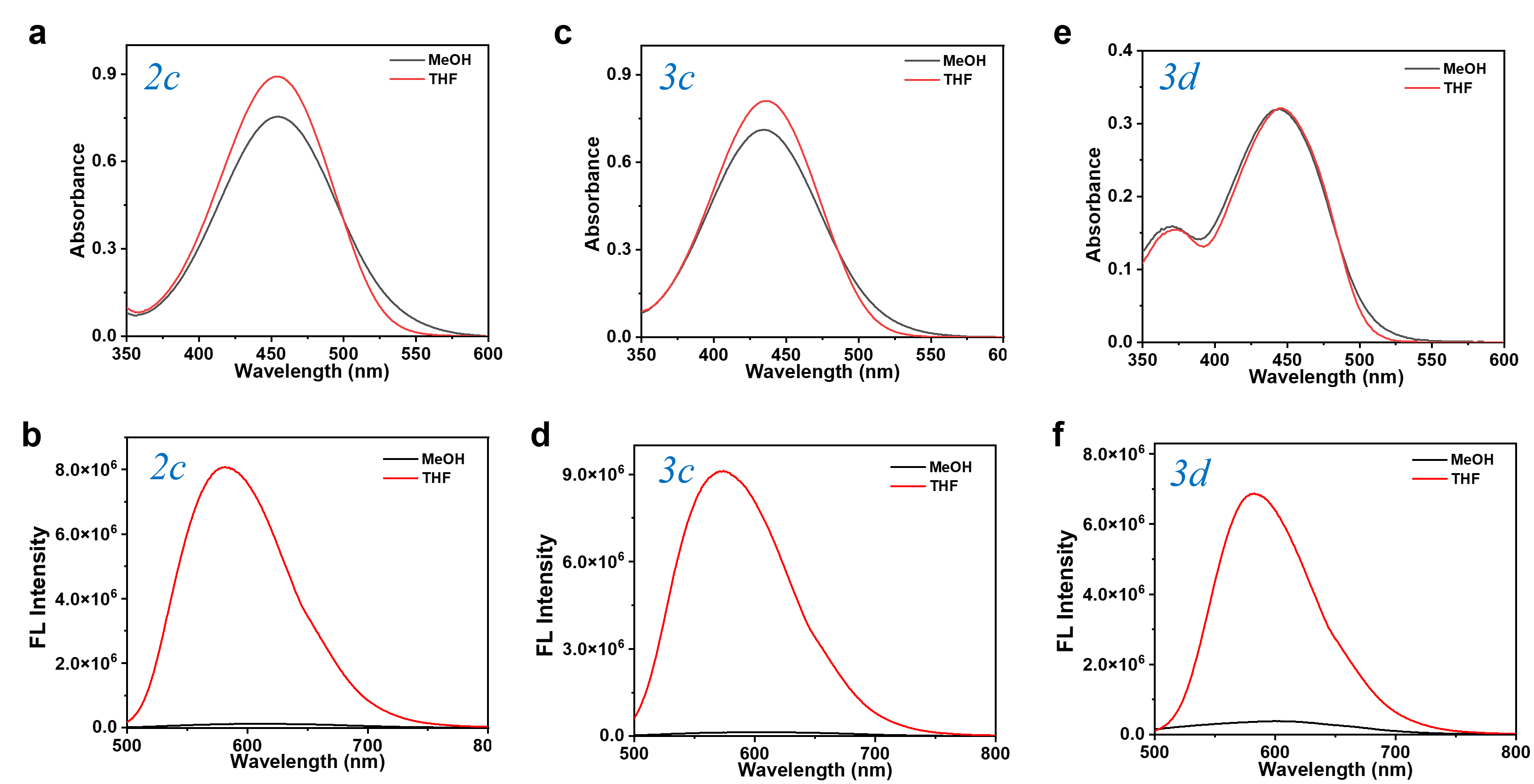


**Figure S2** The absorption spectra and fluorescence emission spectra of 2c/2d and 3c in MeOH and THF


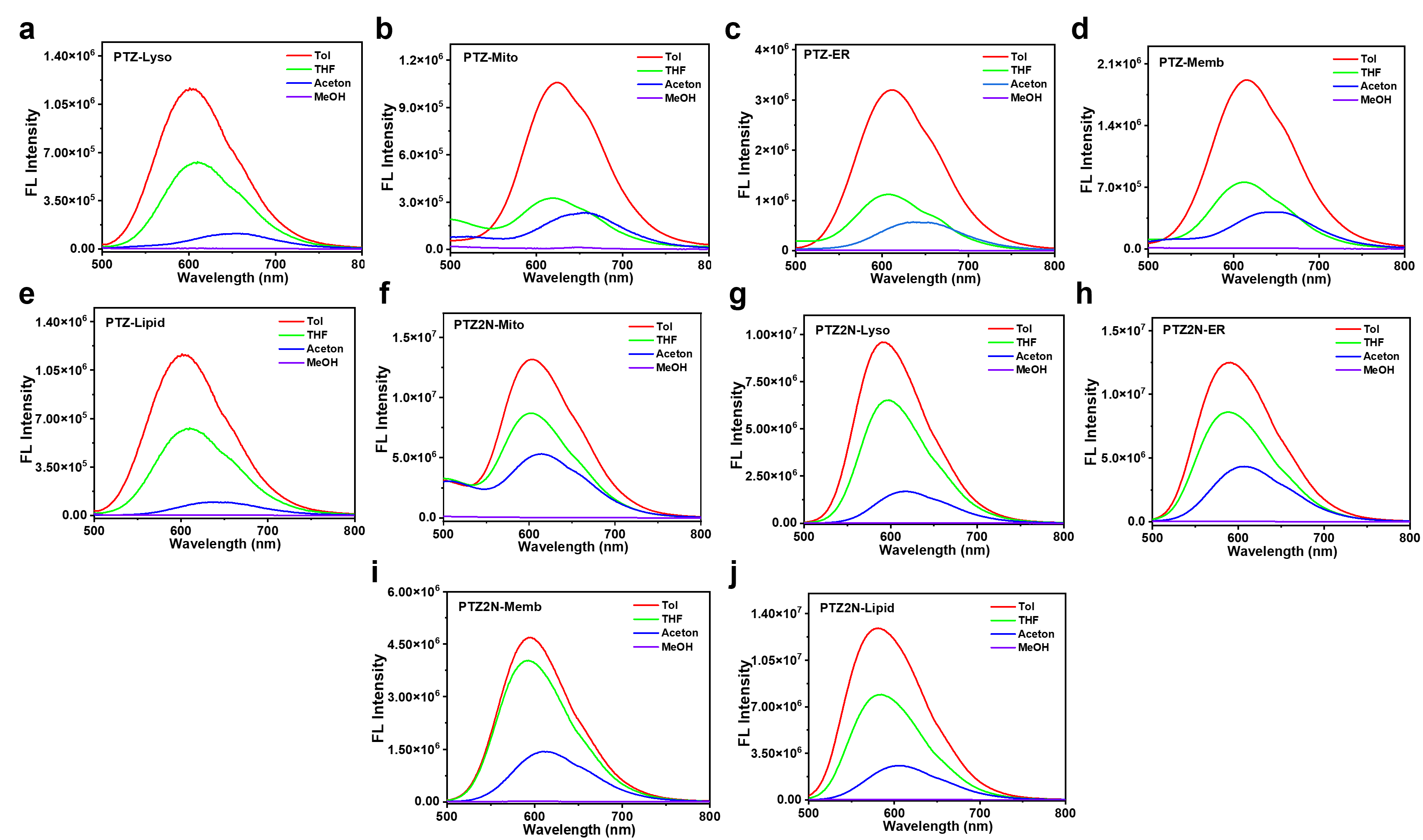


**Figure S3** Fluorescence spectra of phenothiazine-based probes (Concentration: 15 Μm) in different polarity solvents (Toluene (Tol, Dielectric constant: 2.3), THF (Dielectric constant: 7.5), Acetone (Dielectric constant: 21), and Methanol (MeOH, Dielectric constant: 33))

**
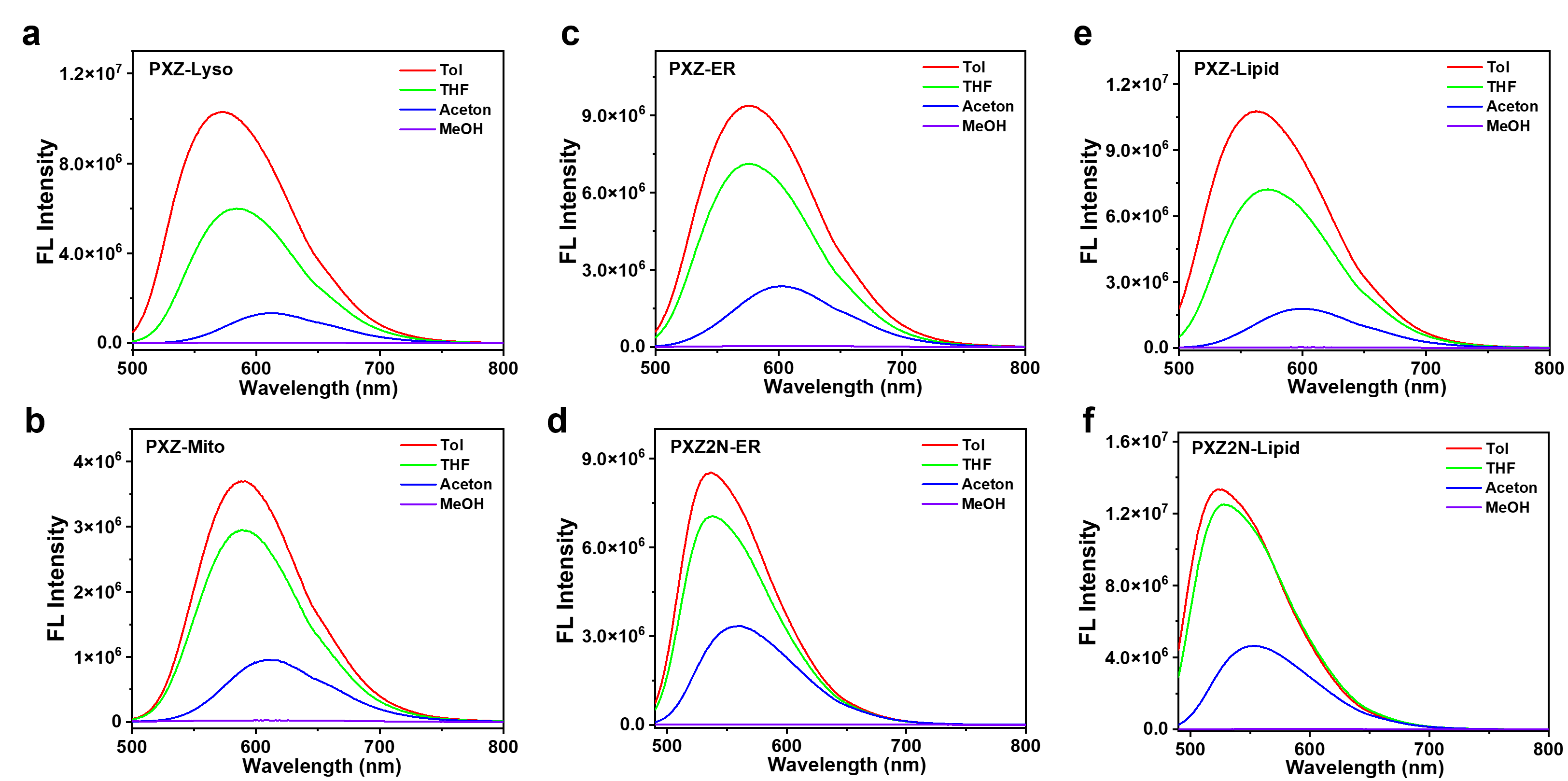
Figure S4** Fluorescence spectra of phenoxazine-based probes (Concentration: 15 Μm) in different polarity solvents


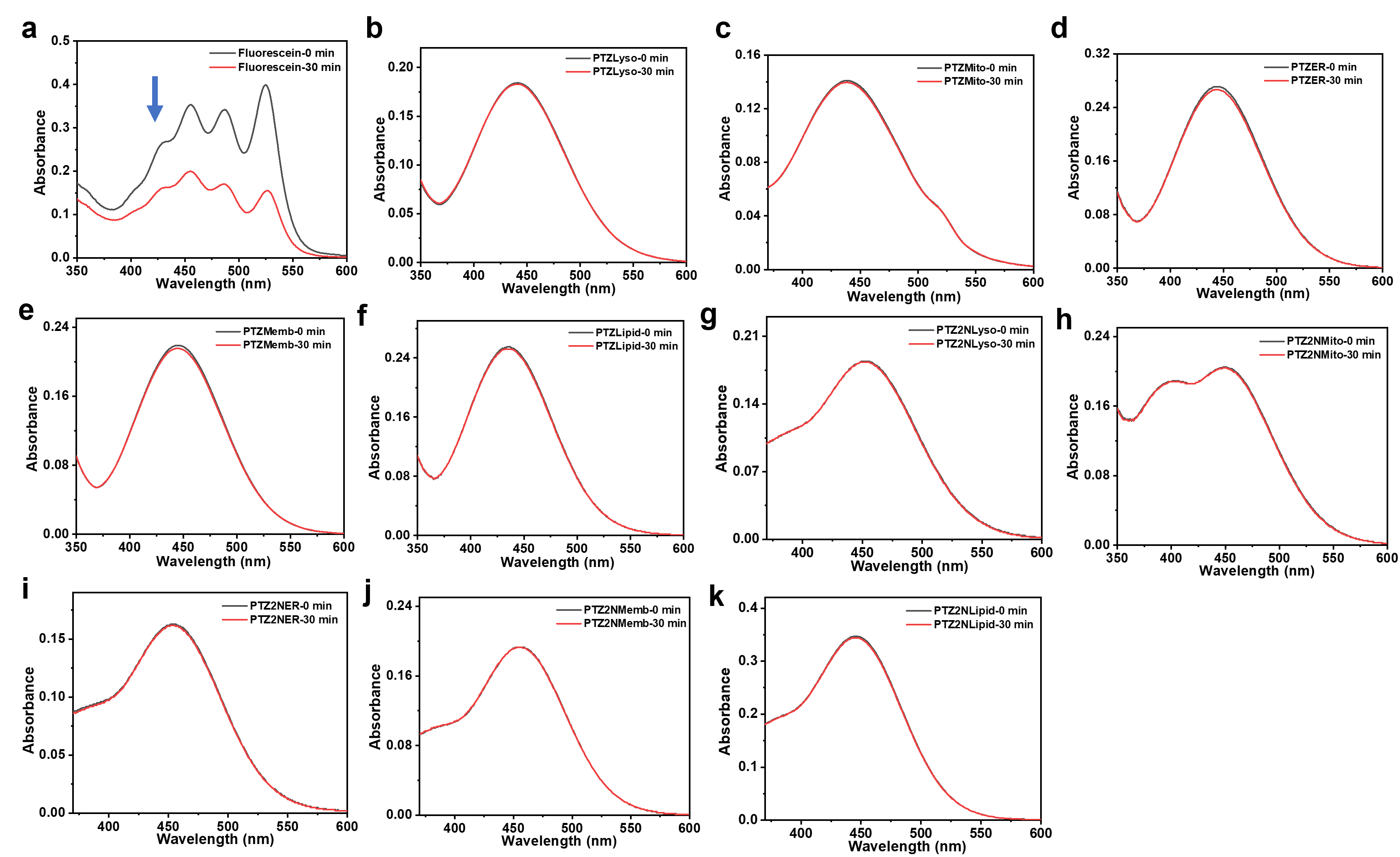


**Figure S5** UV-vis absorption decay of Fluorescein, phenothiazine-based probes under lamp irradiation for 30 minutes


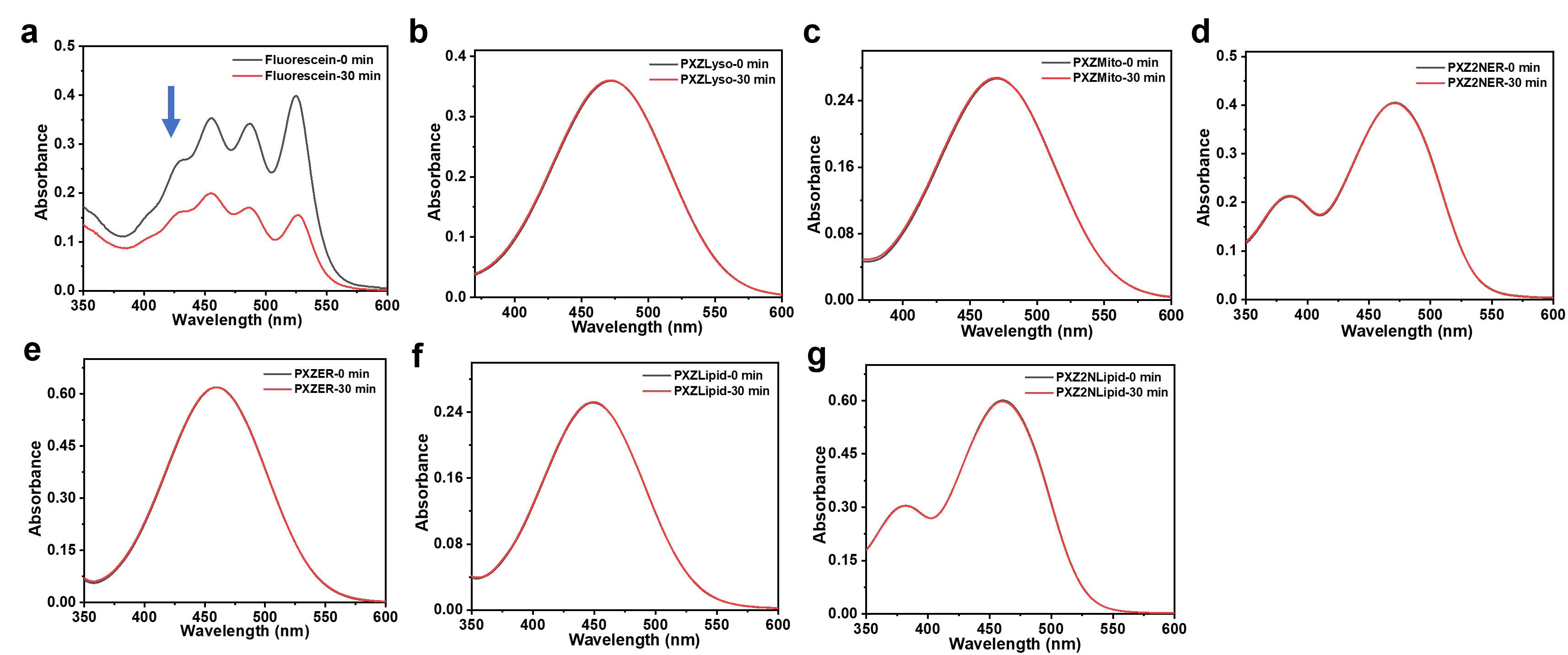


**Figure S6** UV-vis absorption decay of Fluorescein, phenoxazine-based probes under lamp irradiation for 30 minutes


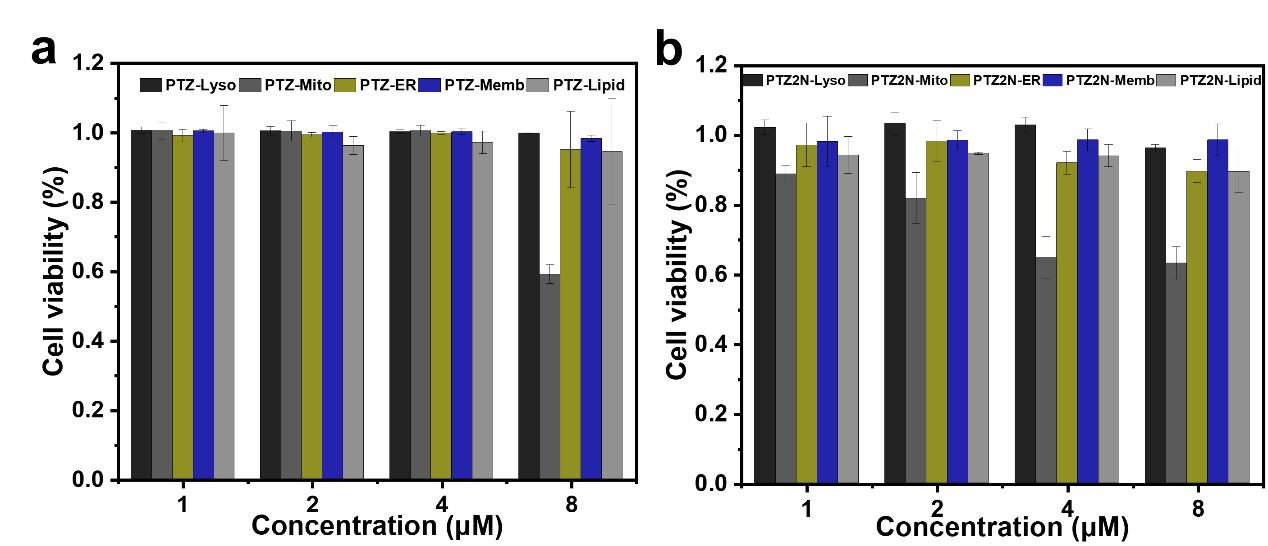


**Figure S7** Viabilities of HeLa cells (n = 3) after incubation with different concentrations of PTZ series molecules for 24h


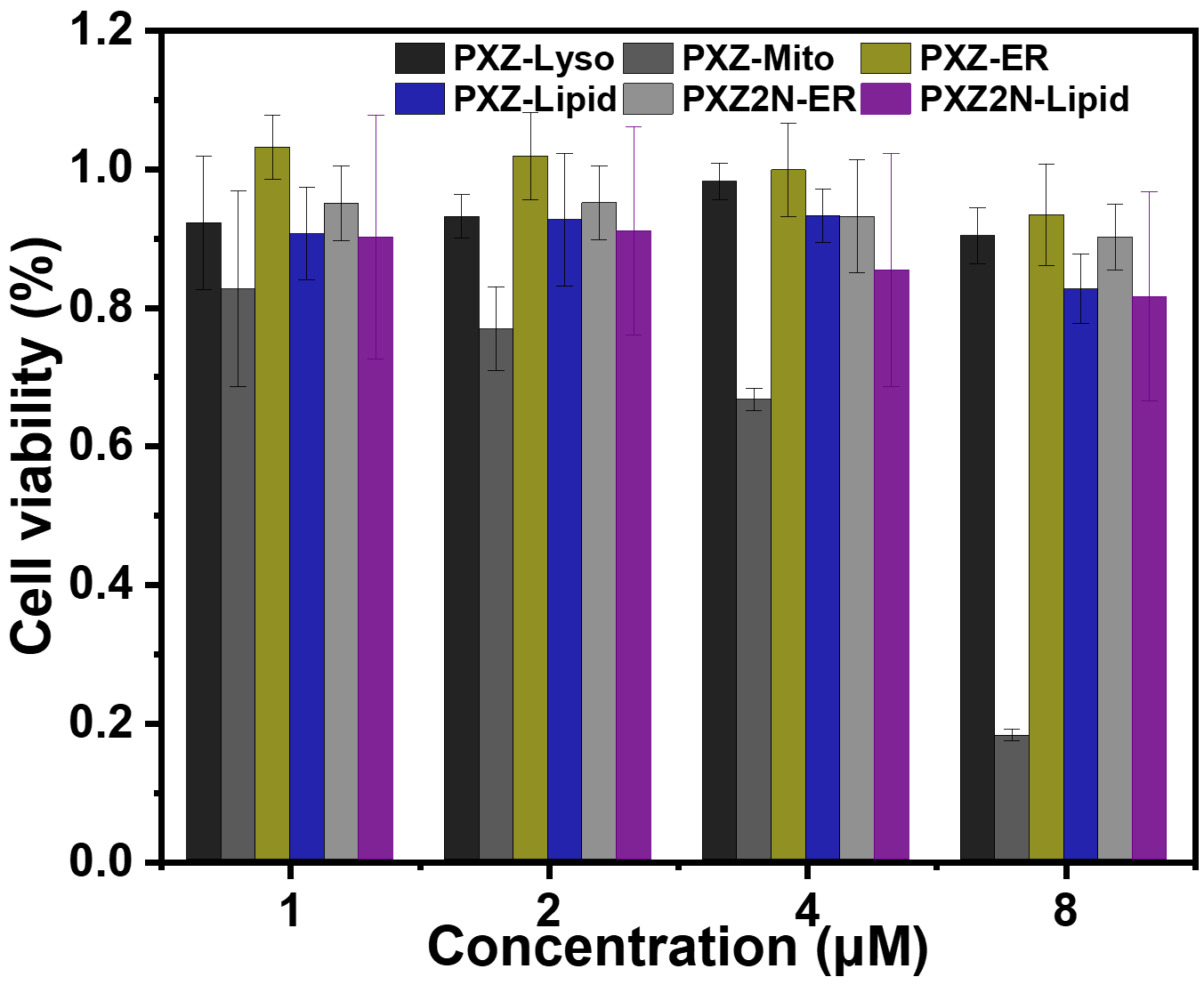


**Figure S8** Viabilities of HeLa cells (n = 3) after incubation with different concentrations of PXZ series molecules for 24h

**
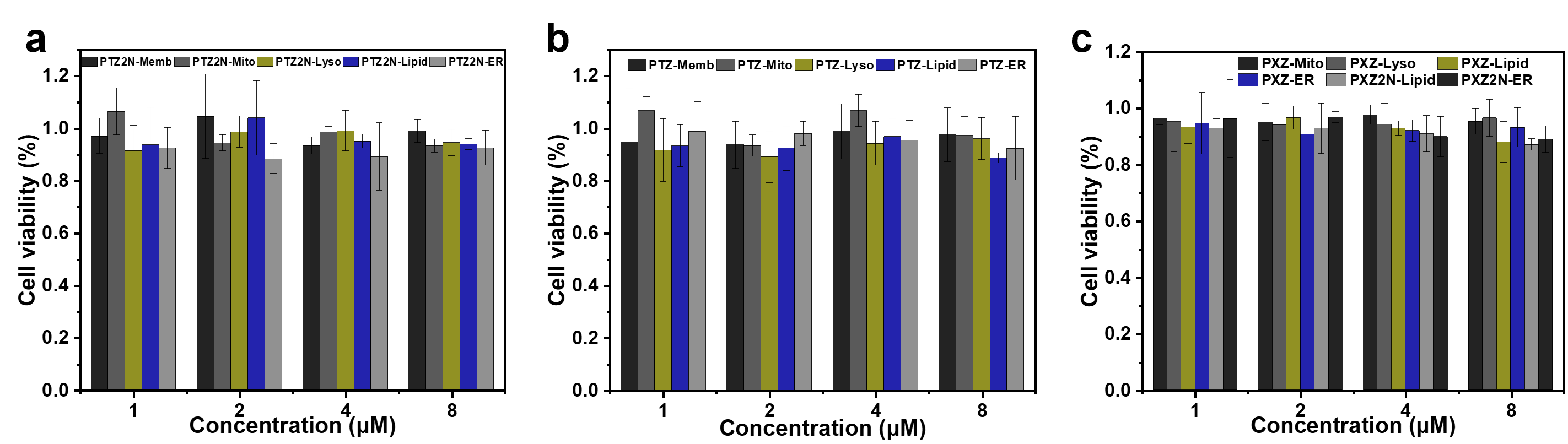
**

**Figure S9** Viability of HeLa cells (n = 3) after incubation with various concentrations of PTZ/PXZ series molecules for 1 hour, followed by irradiation with a 450 nm LED lamp (15 mW·cm⁻²) for 30 minutes, and subsequent incubation with fresh medium for an additional 8 hours**
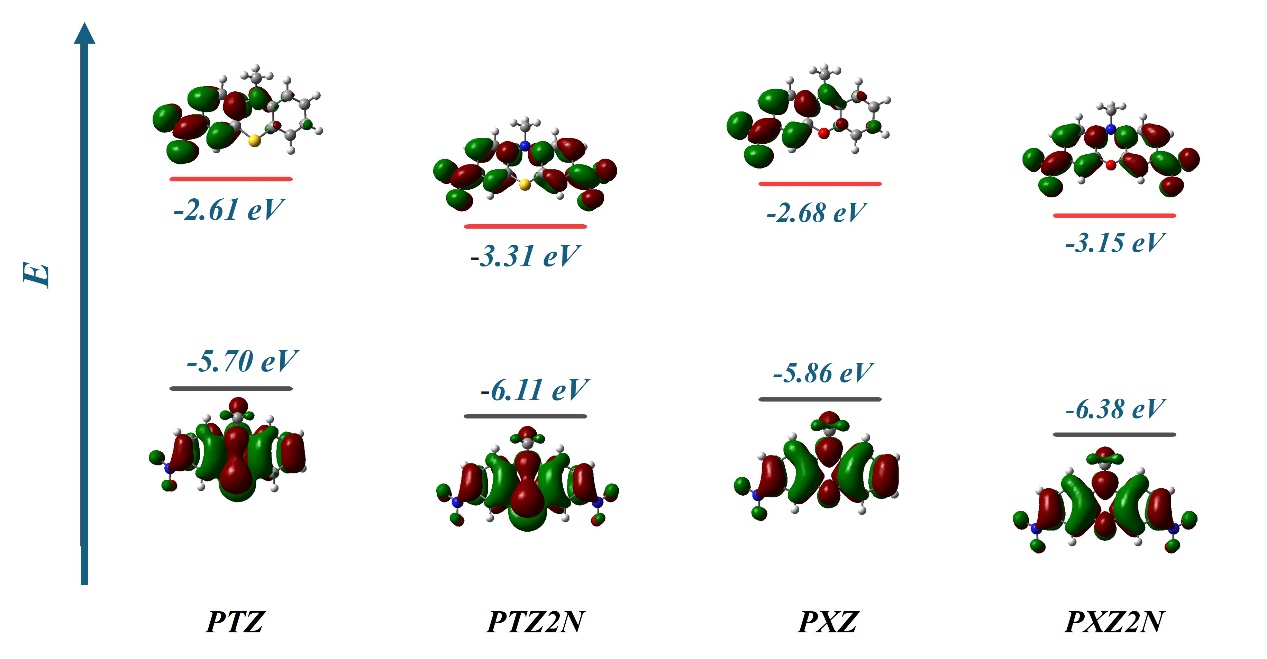
**

**Figure S10** HOMO/LUMO energy levels of PTZ/PTZ2N and PXZ/PXZ2N calculated by the b3lyp/6-31+g(d,p) basis set

**Table 1** Photophysical properties for phenoxazine/phenothiazine-based probes

| **Compound** | **λ_abs (nm)_^a^** | **λ_emi (nm)_^a^** | **Δλ _(nm)_^b^** | **φ^c^** | **τ_s (ns)_^d^** | ***k*_r (s_^-1^_)_** | ***k*_nr (s_^-1^_)_** |
| --- | --- | --- | --- | --- | --- | --- | --- |
| **PTZ-Lipid** | **423** | **610** | **187** | **0.13** | **2.14** | **5.99 × 10^7^** | **4.07 × 10^8^** |
| **PTZ2N-Lipid** | **435** | **606** | **171** | **0.24** | **7.81** | **3.01 × 10^7^** | **9.79× 10^7^** |
| **PXZ-Lipid** | **436** | **575** | **139** | **0.17** | **2.71** | **6.27 × 10^7^** | **3.06 × 10^8^** |
| **PXZ2N-Lipid** | **446** | **531** | **85** | **0.68** | **6.34** | **1.08 × 10^8^** | **5.01× 10^7^** |
| **PTZ-Lyso** | **424** | **613** | **189** | **0.13** | **1.93** | **6.66 × 10^7^** | **4.52 × 10^8^** |
| **PTZ2N-Lyso** | **435** | **600** | **165** | **0.24** | **7.11** | **3.34 × 10^7^** | **1.07 × 10^8^** |
| **PXZ-Lyso** | **454** | **585** | **131** | **0.19** | **2.34** | **7.95 × 10^7^** | **3.48 × 10^8^** |
| **PTZ-Mito** | **428** | **619** | **191** | **0.14** | **2.11** | **6.62 × 10^7^** | **4.08 × 10^8^** |
| **PTZ2N-Mito** | **446** | **592** | **146** | **0.24** | **1.96** | **1.21 × 10^8^** | **3.89 × 10^8^** |
| **PXZ-Mito** | **458** | **589** | **131** | **0.19** | **0.79** | **2.36 × 10^8^** | **1.03 × 10^8^** |
| **PTZ-ER** | **429** | **588** | **159** | **0.16** | **4.51** | **3.61 × 10^7^** | **1.86 × 10^8^** |
| **PTZ2N-ER** | **433** | **586** | **153** | **0.18** | **5.86** | **3.21 × 10^7^** | **1.39 × 10^8^** |
| **PXZ-ER** | **443** | **576** | **133** | **0.18** | **2.42** | **7.37 × 10^7^** | **3.40 × 10^8^** |
| **PXZ2N-ER** | **453** | **535** | **82** | **0.58** | **5.81** | **1.01 × 10^8^** | **7.16× 10^7^** |
| **PTZ-Memb** | **427** | **607** | **180** | **0.14** | **2.08** | **6.51 × 10^7^** | **4.16 × 10^8^** |
| **PTZ2N-Memb** | **422** | **605** | **183** | **0.19** | **2.76** | **6.84 × 10^7^** | **2.94 × 10^8^** |

**a: the maximum absorption peak and the maximum emission peak of probes, measure in THF**

**b: Stokes shift = λ_emi_ – λ_abs_**

**c: Fluorescence quantum yields, measured by integrated sphere in Tol**

**d: Fluorescence lifetime**

**
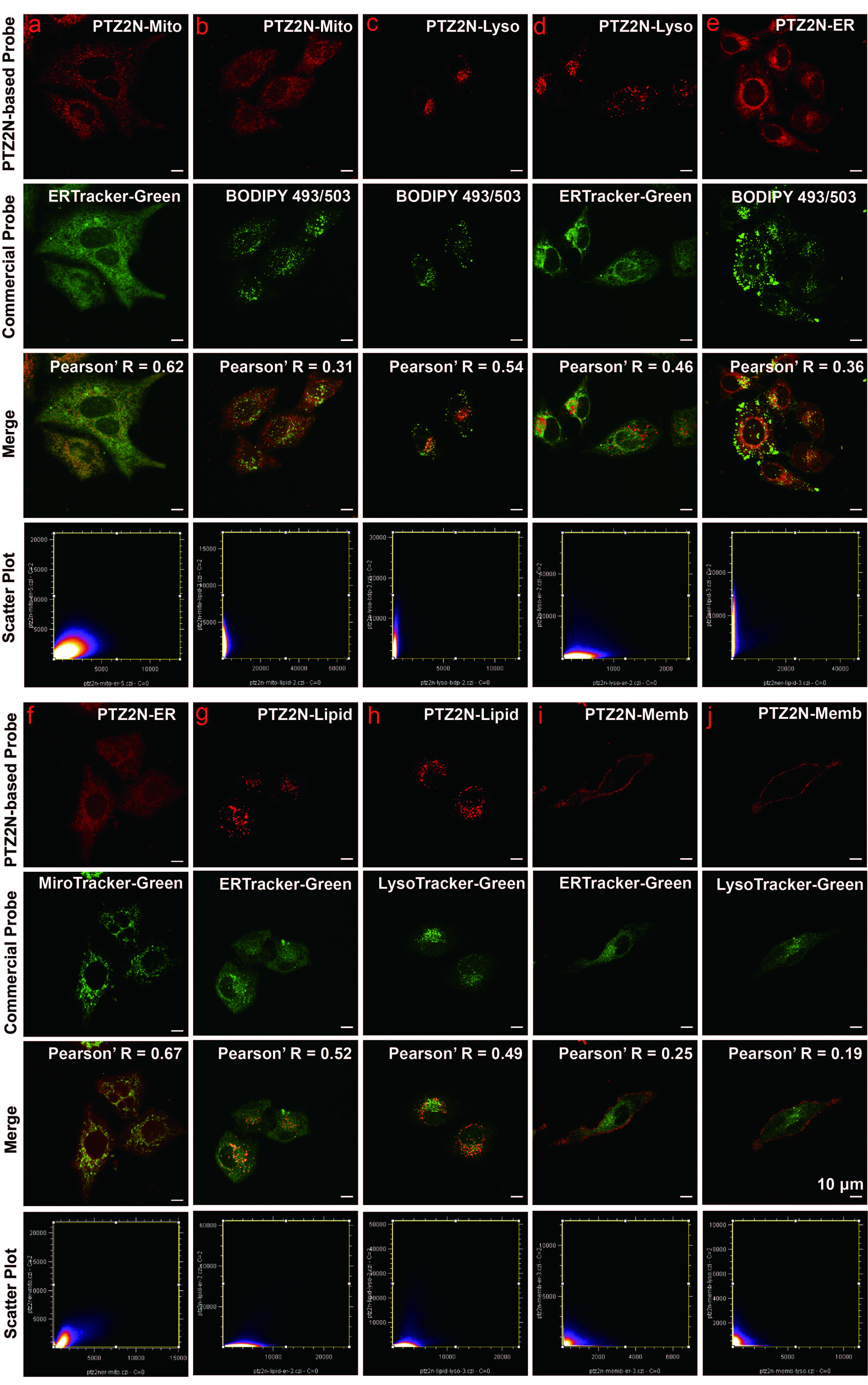
**

**Figure S11** Colocalization of PTZ2N-based probes (in red channel) with commercial organelle probes (in green channel) in live HeLa cells.


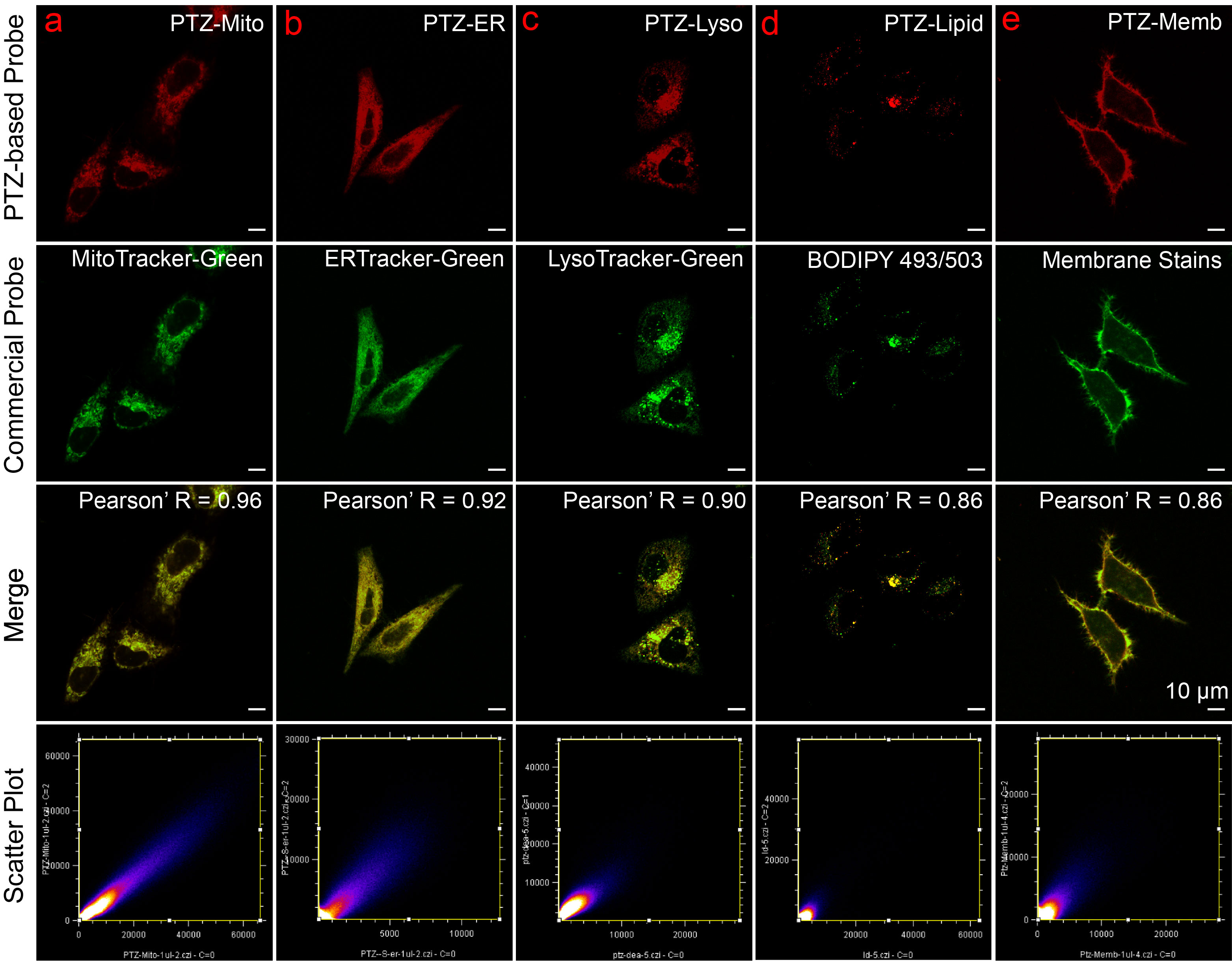


**Figure S12** Colocalization of PTZ-based probes (in red channel) with commercial organelle probes (in green channel) in live HeLa cells.

**
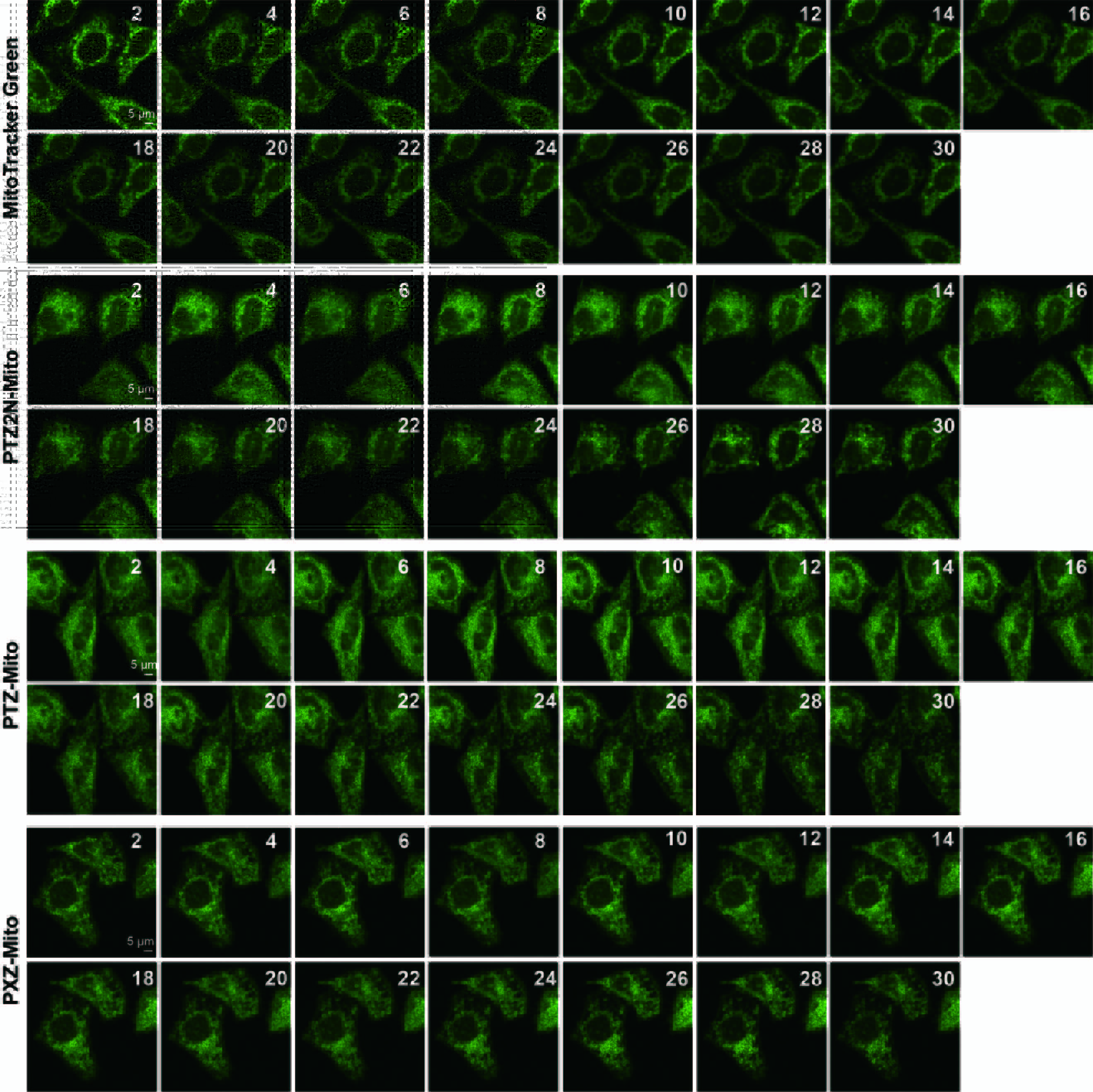
**

**Figure S13** Fluorescence intensity changes of commercial dyes, PTZ2N-Mito, PTZ-Mito, and PXZ-Mito during 30 minutes of irradiation (λ_ex_ = 488 nm; 60% laser intensity; Pixel dwell: 1.40 μsec; Scan time:1 min 58 sec; speed: 6; Averaging number: 16)


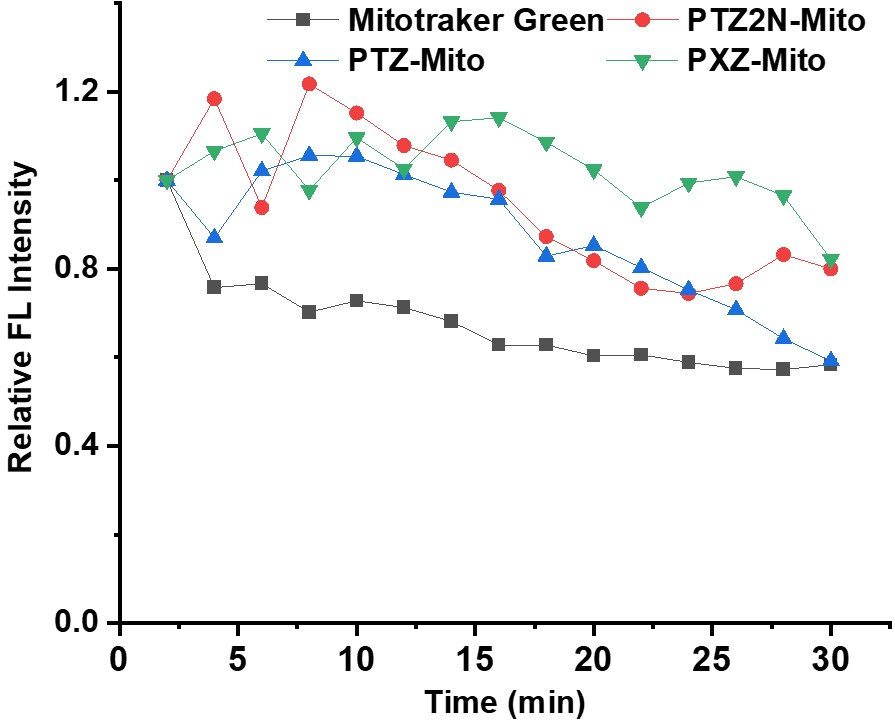


**Figure S14** Variation in mean fluorescence intensity of HeLa cells during 30 minutes of continuous laser irradiation


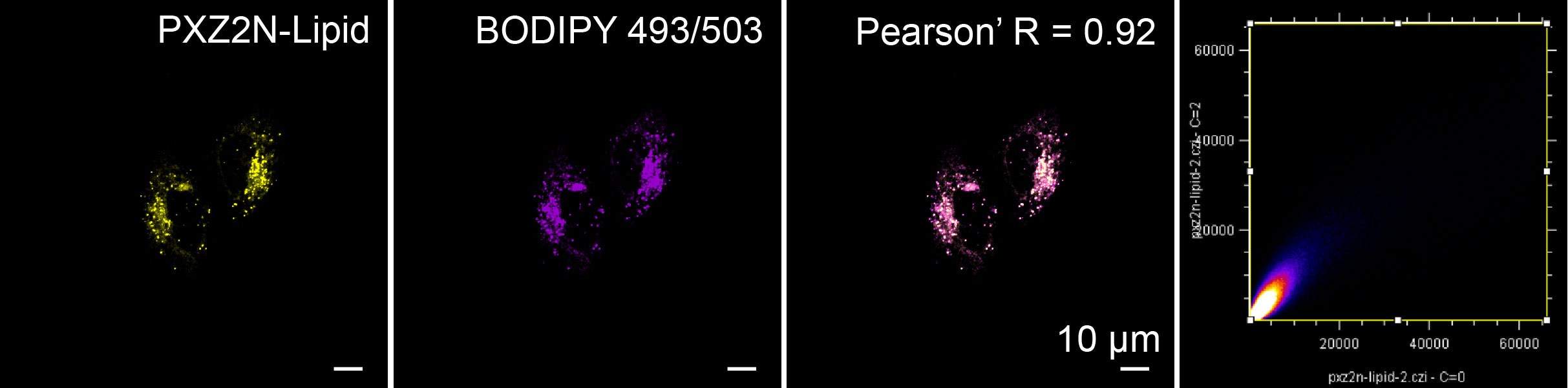


**Figure S15** Colocalization of PXZ2N-Lipid probes (in the yellow channel) with commercial organelle probes (in the purple channel) in live HeLa cells

**
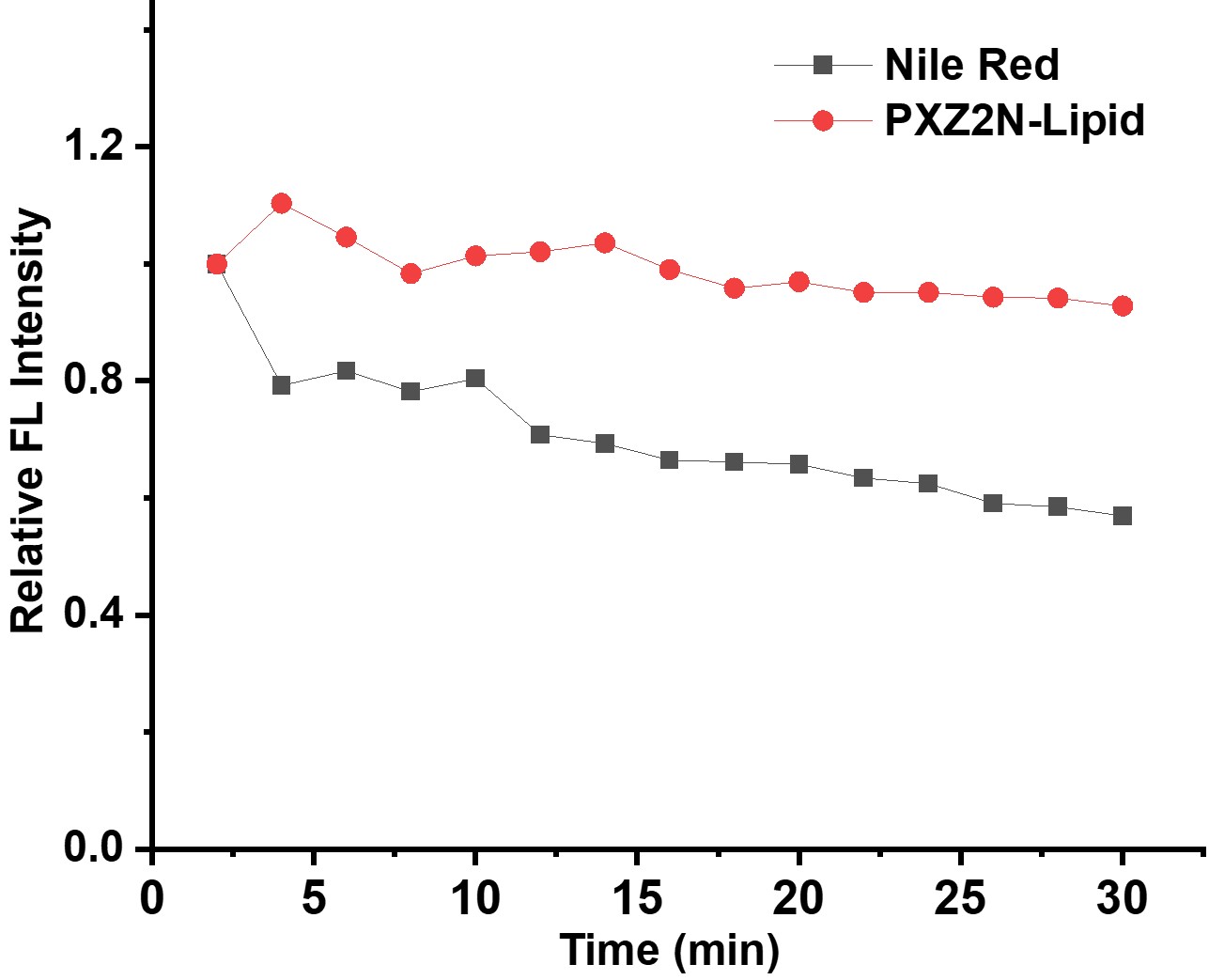
**

**Figure S16** Variation in mean fluorescence intensity of HeLa cells during 30 minutes of continuous laser irradiation


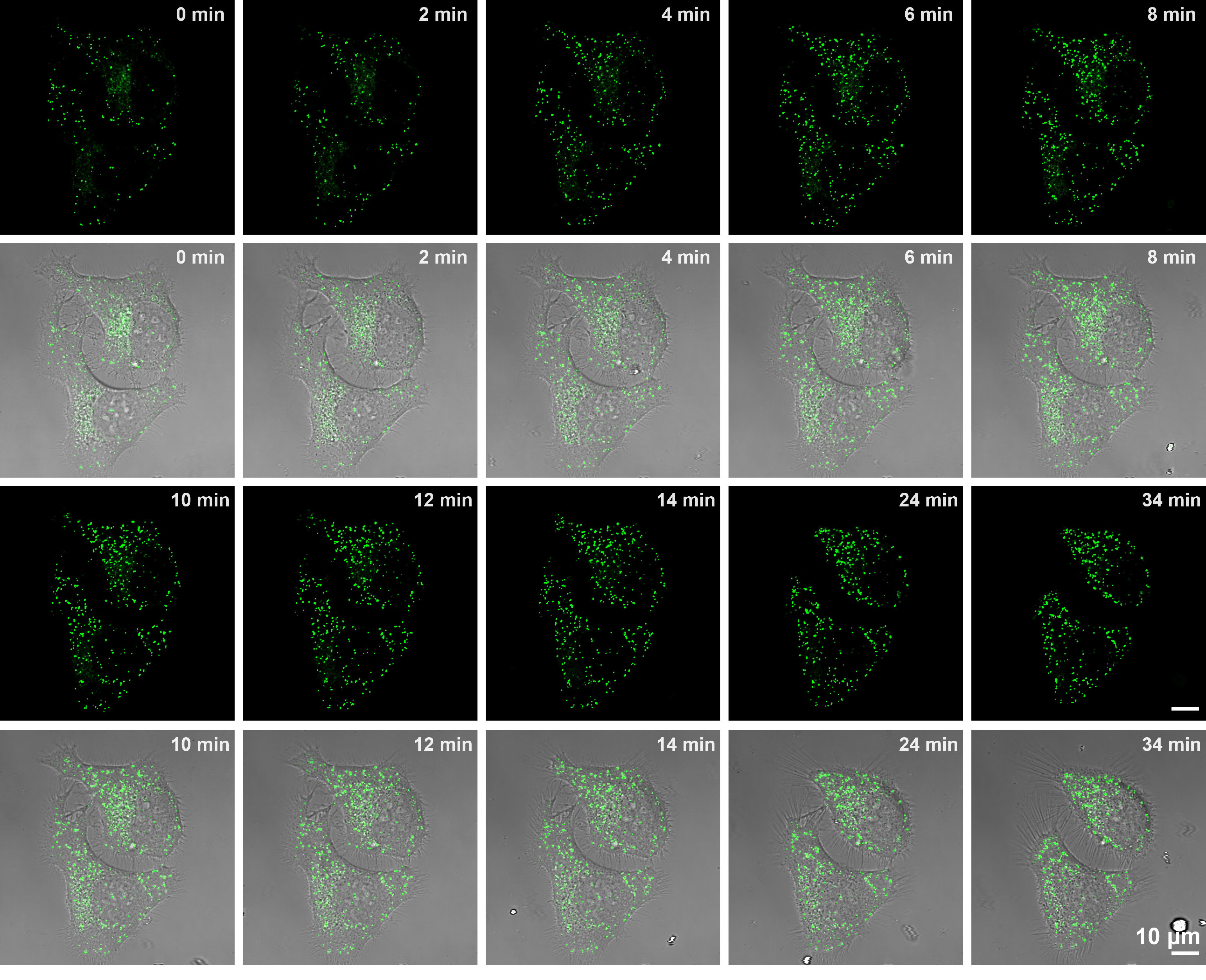


**Figure S17** In situ monitoring lipid droplets dynamic in oleic acid-treated SKOV3 cells stained with PXZ-Lipid. Scale bar: 10 μM

**
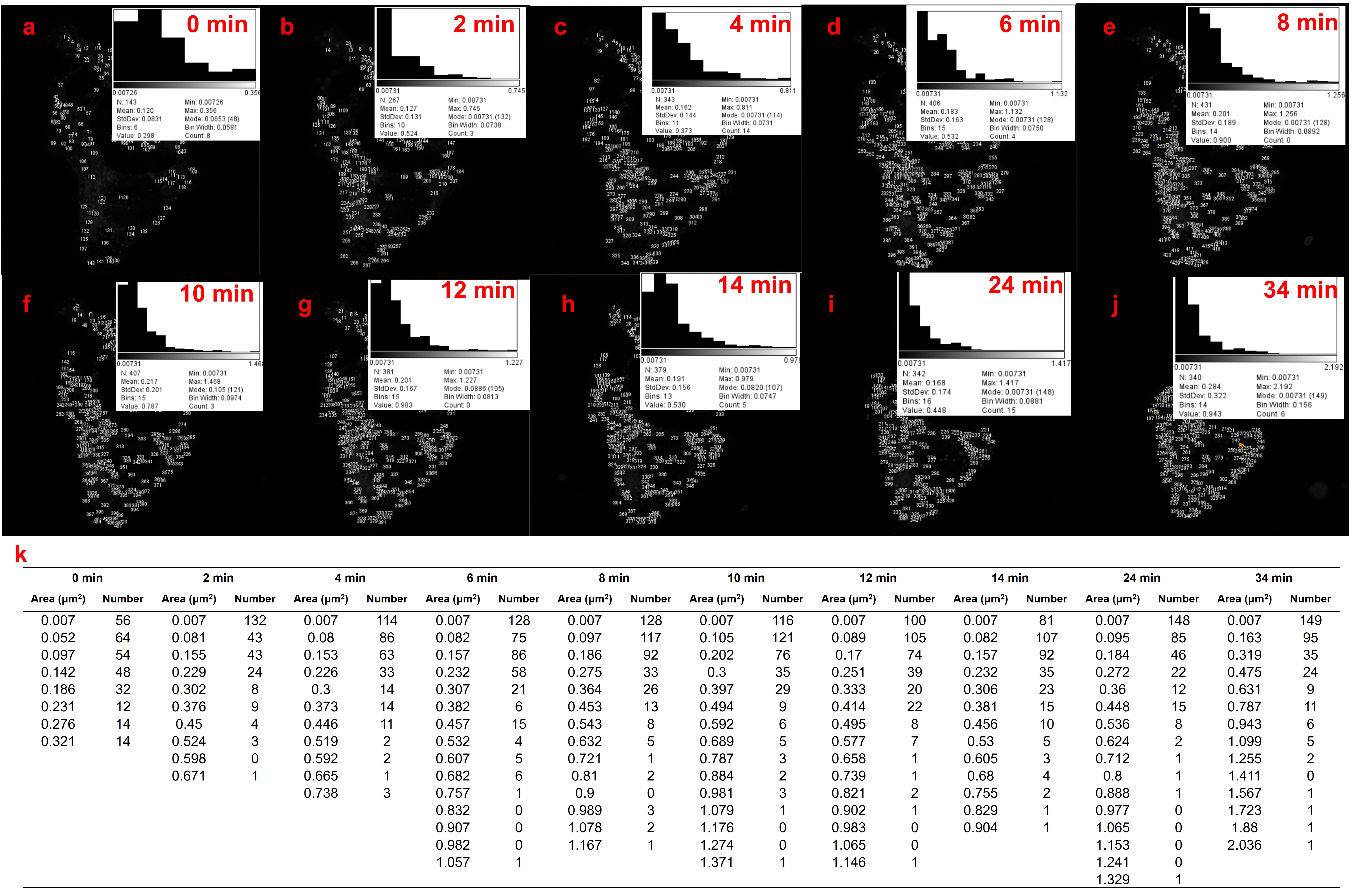
**

**Figure S18** Automatic quantification of lipid droplet dynamics in oleic acid-treated SKOV3 cells stained with PXZ-Lipid, analyzed using ImageJ. (a–j) Lipid droplet counts in SKOV3 cells at different time points (inset: area distribution of lipid droplets). (k) Quantitative analysis of lipid droplet size and number over time


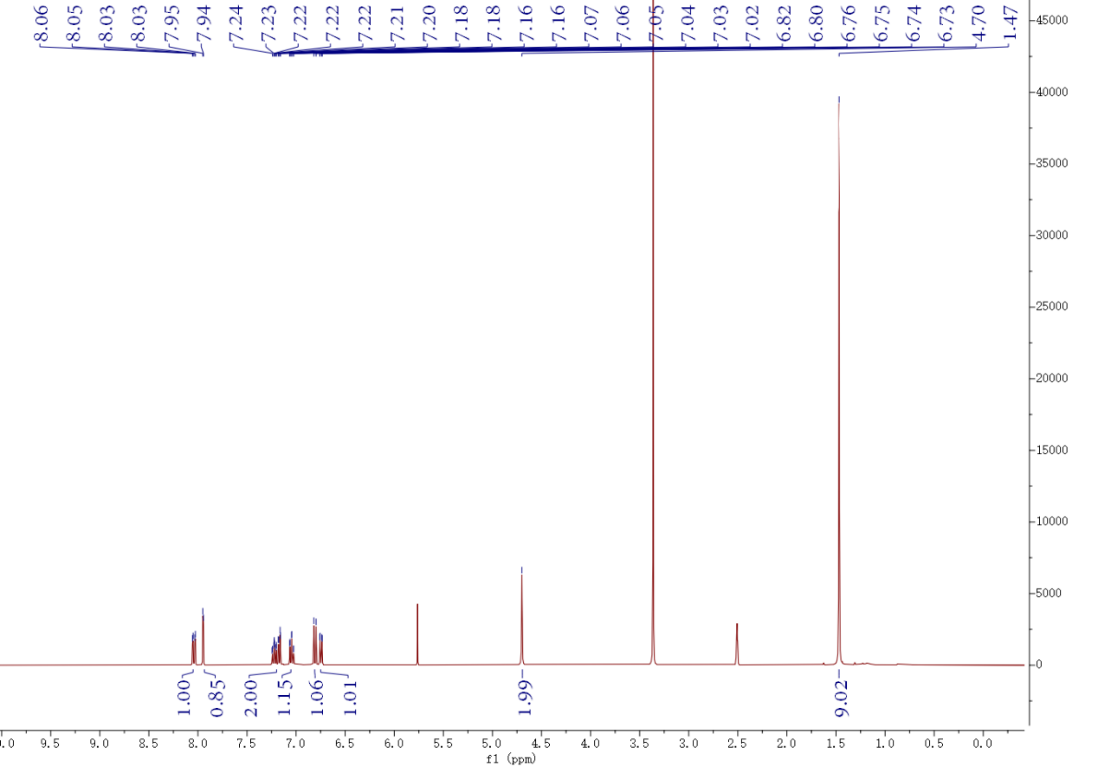


^1^H NMR spectrum of PTZ-Lipid (3a) in CDCl_3_


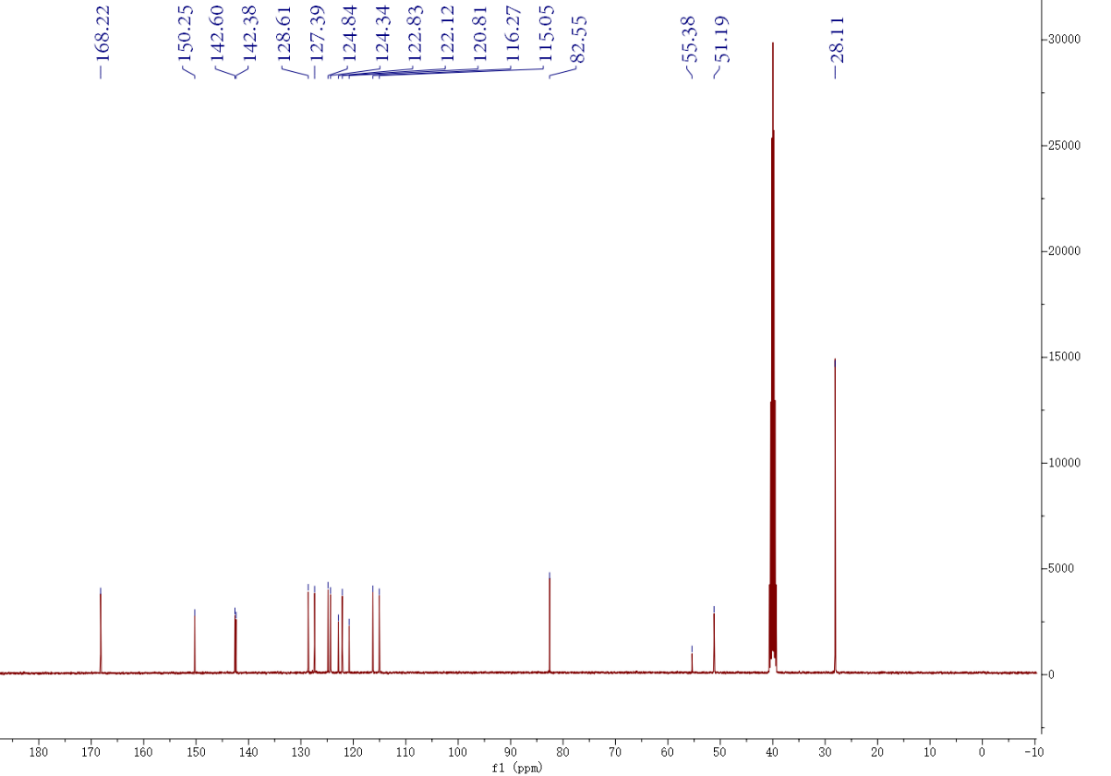


^13^C NMR spectrum of PTZ-Lipid (3a) in CDCl_3_


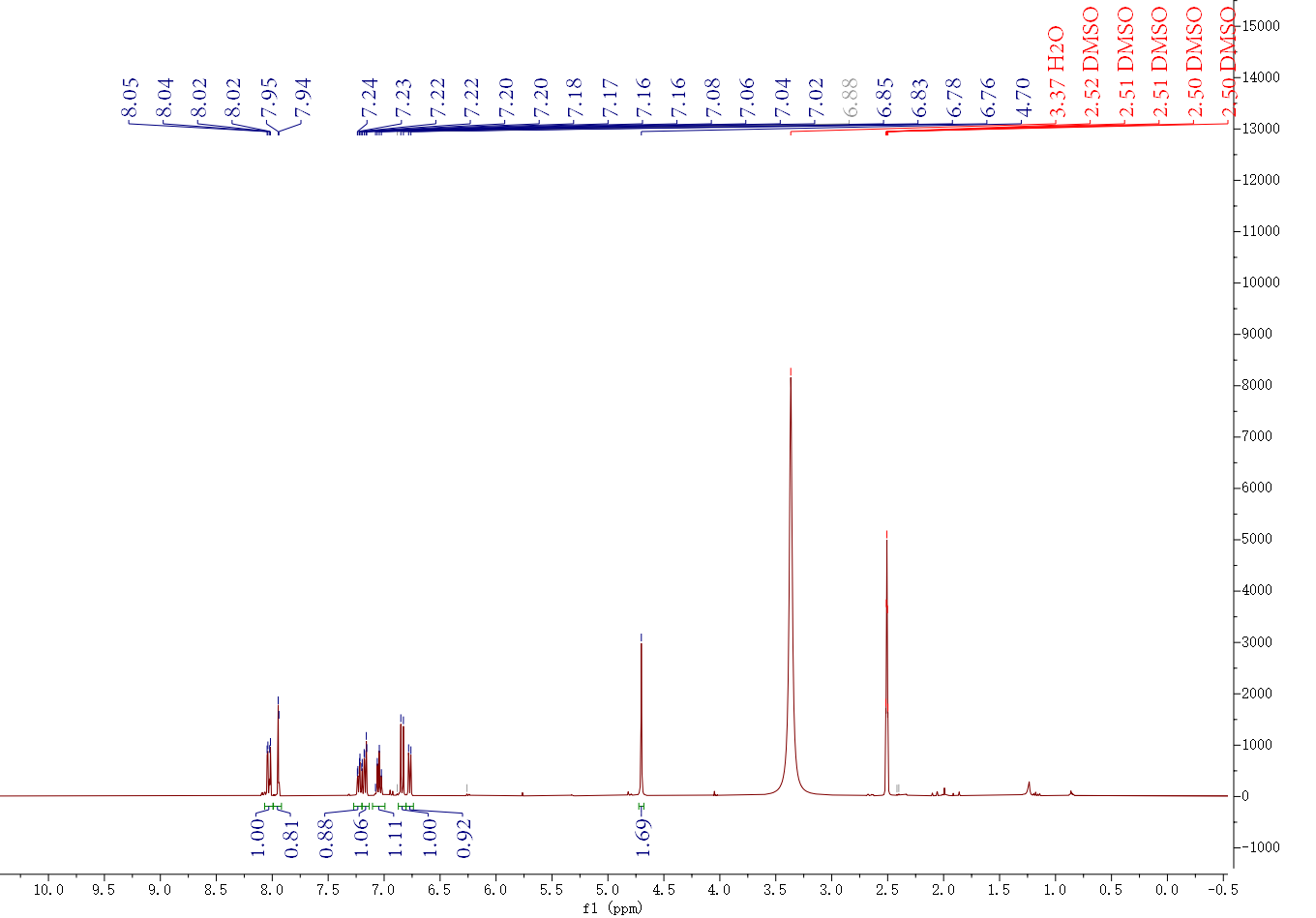


^1^H NMR spectrum of 4a in DMSO-d6


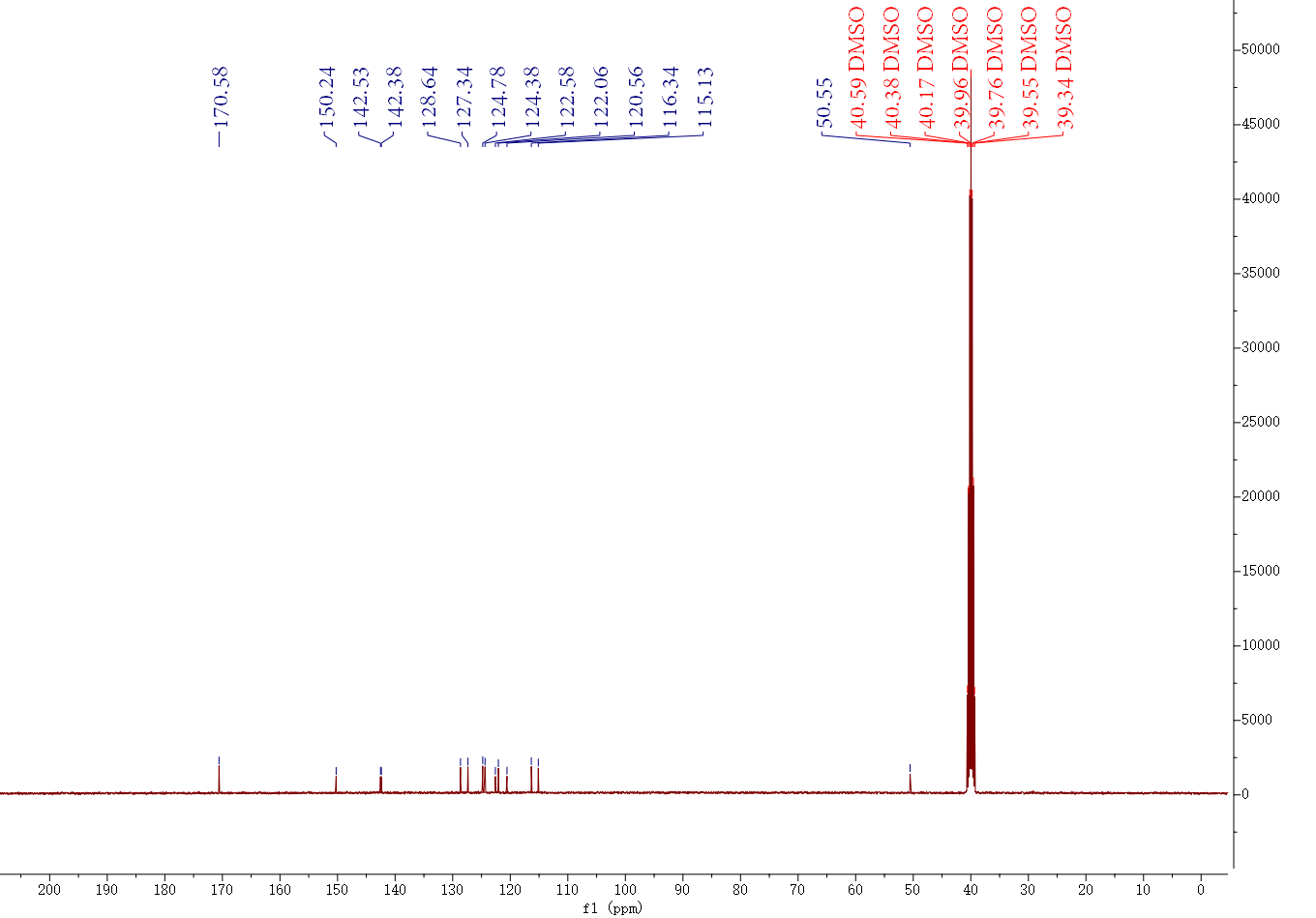


^13^C NMR spectrum of 4a in DMSO-d6


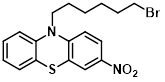

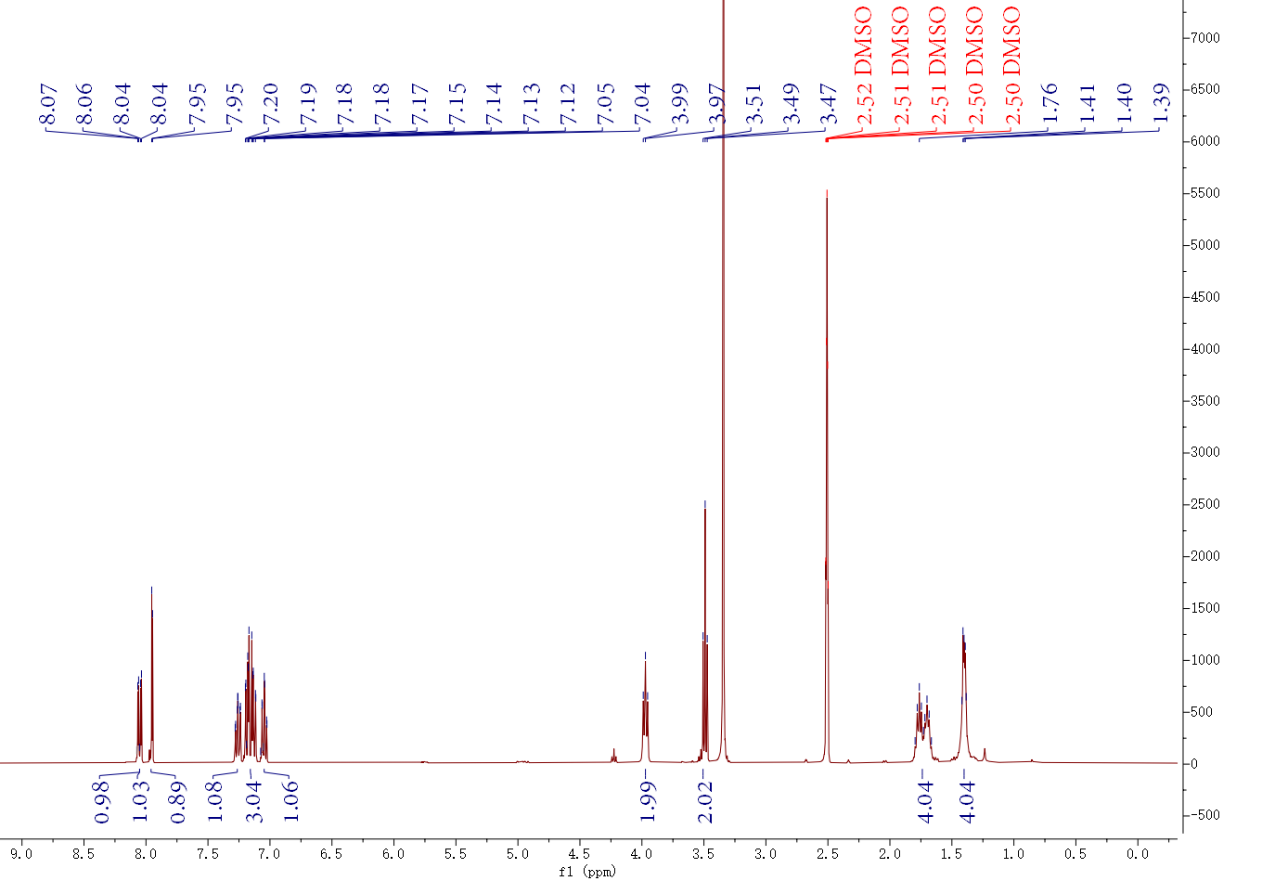


^1^H NMR spectrum of 2a in CDCl_3_


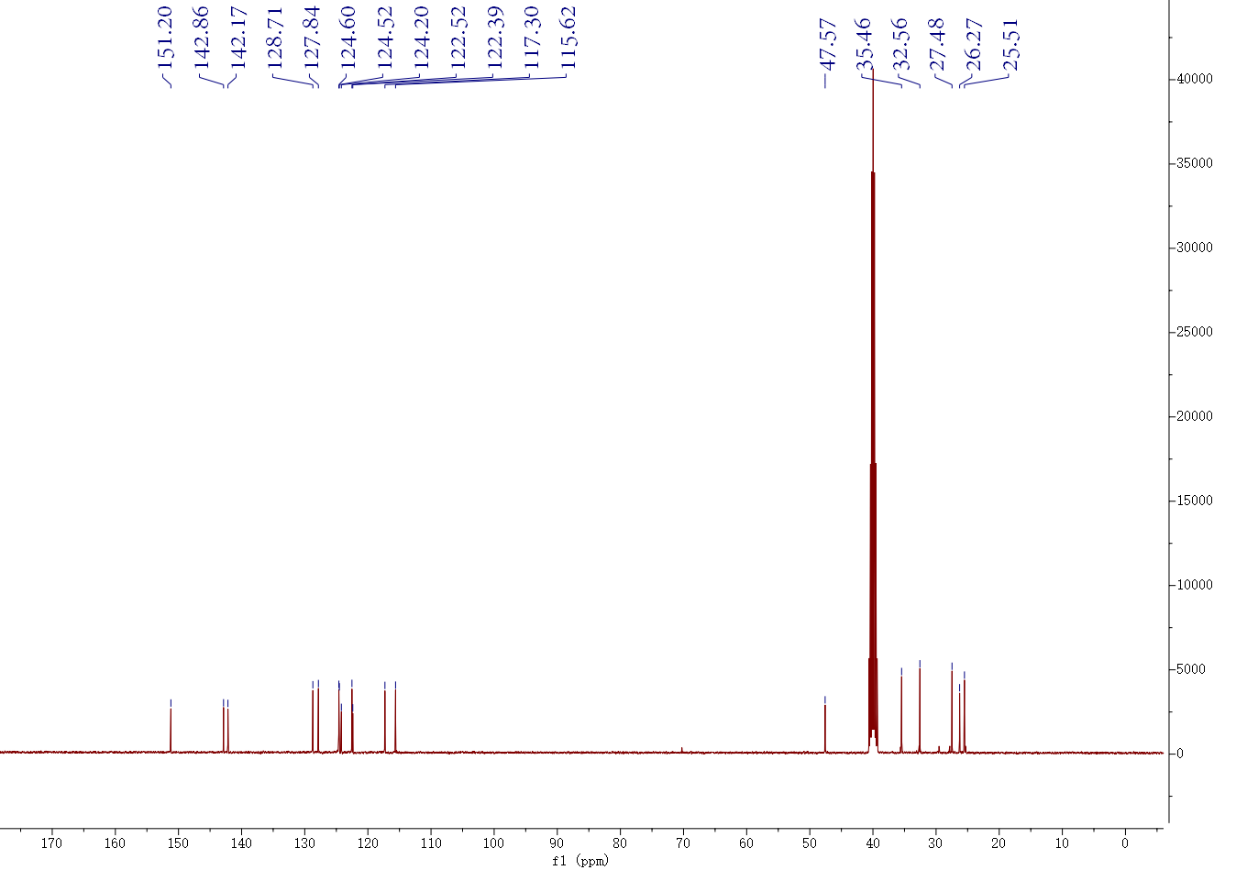
 ^13^C NMR spectrum of 2a in CDCl_3_


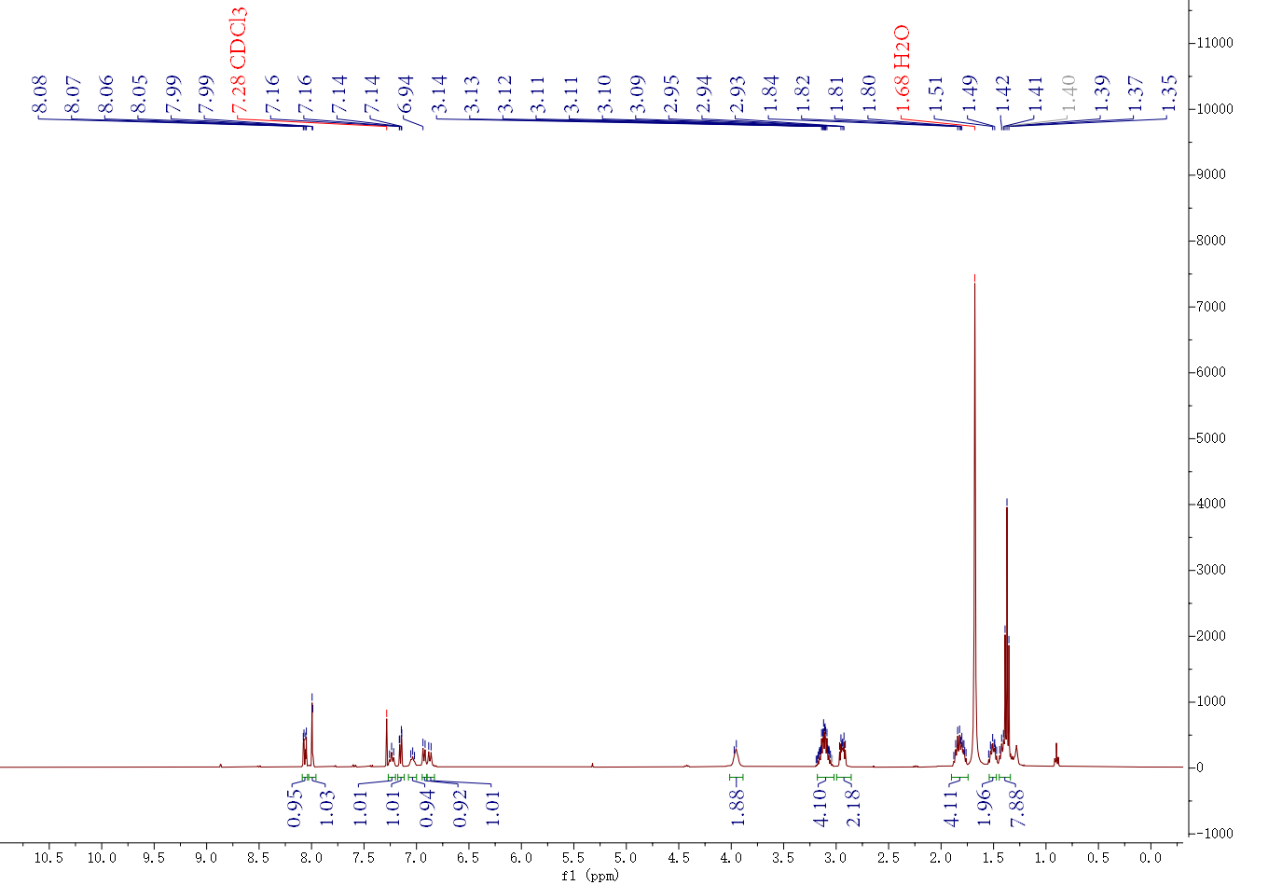


^1^H NMR spectrum of PTZ-Lyso in CDCl_3_
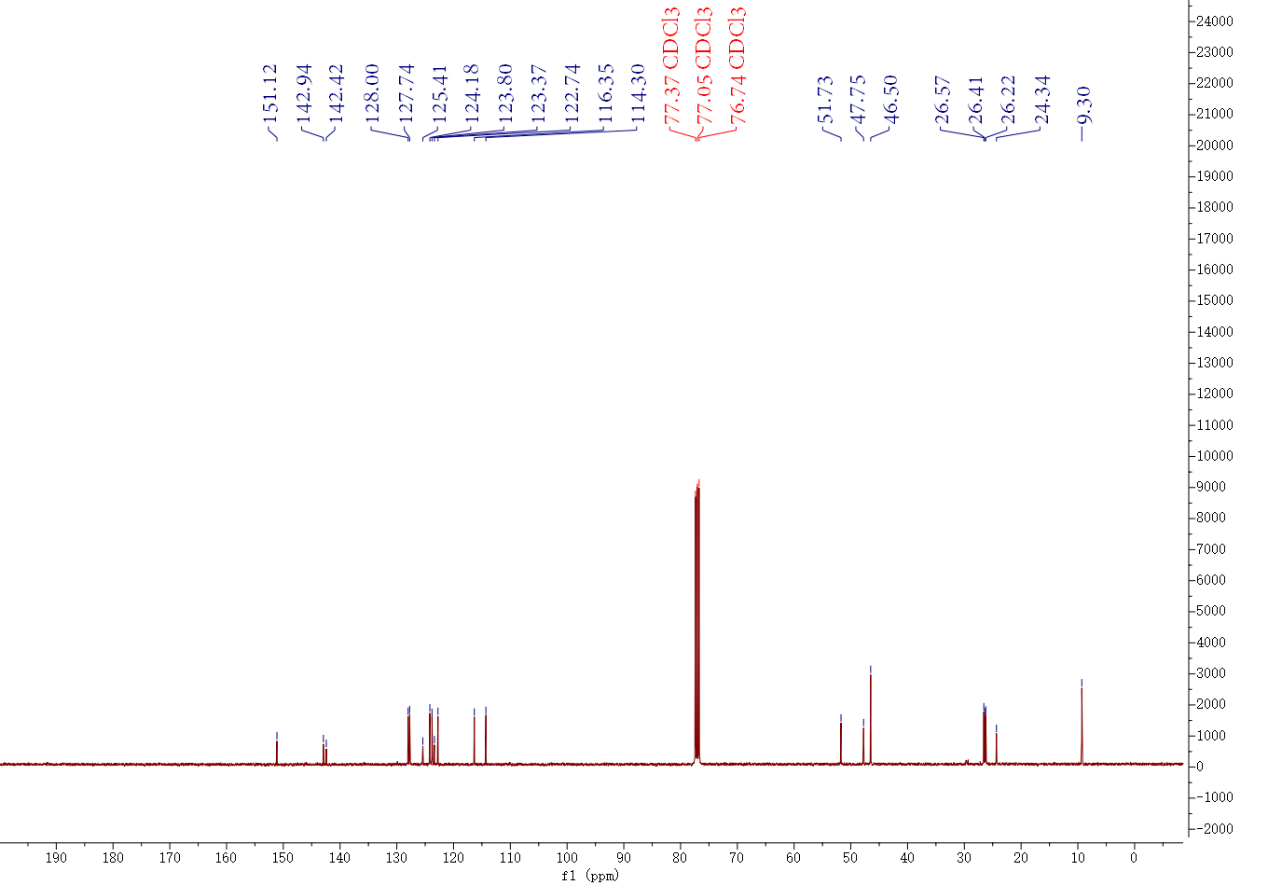


^13^C NMR spectrum of PTZ-Lyso in CDCl_3_


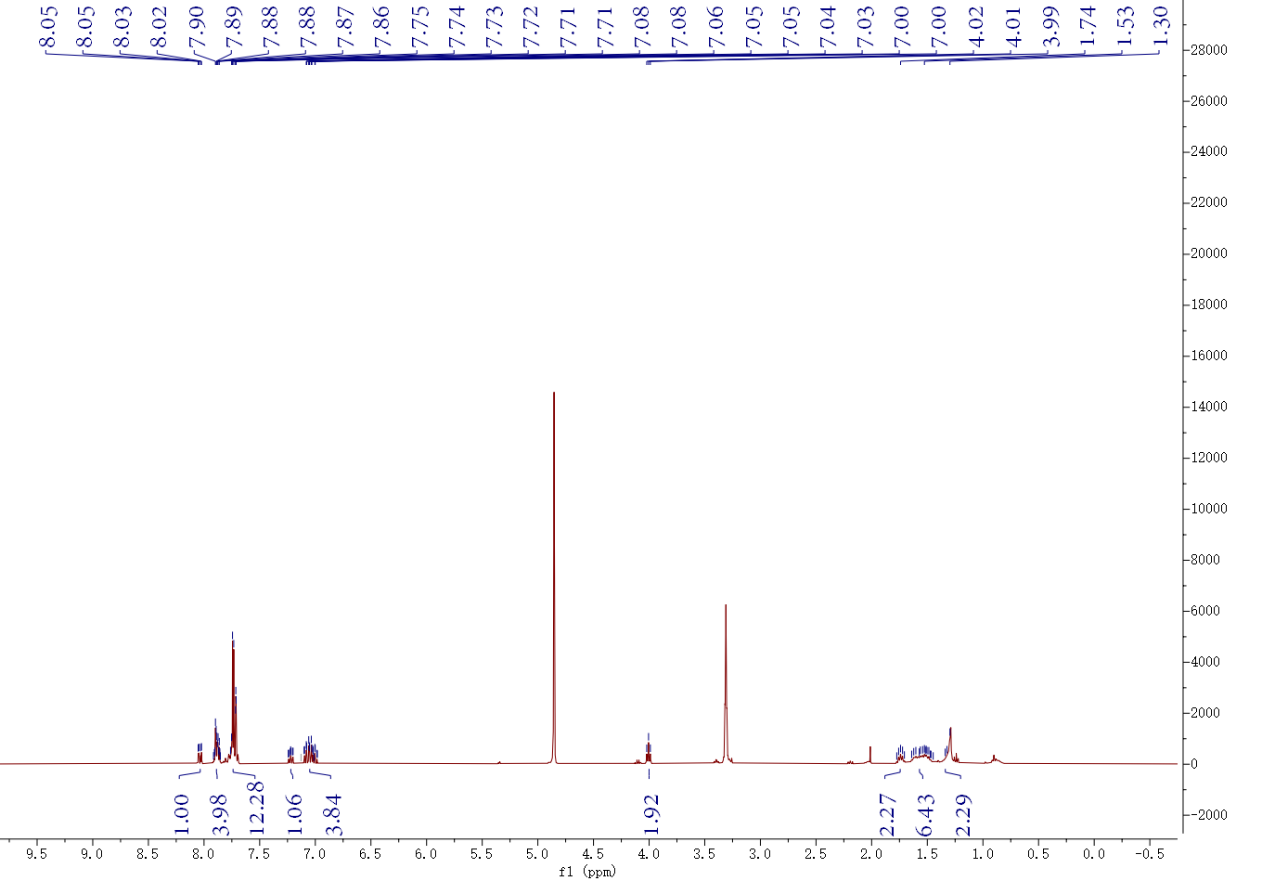


^1^H NMR spectrum of PTZ-Mito in CD_3_OD


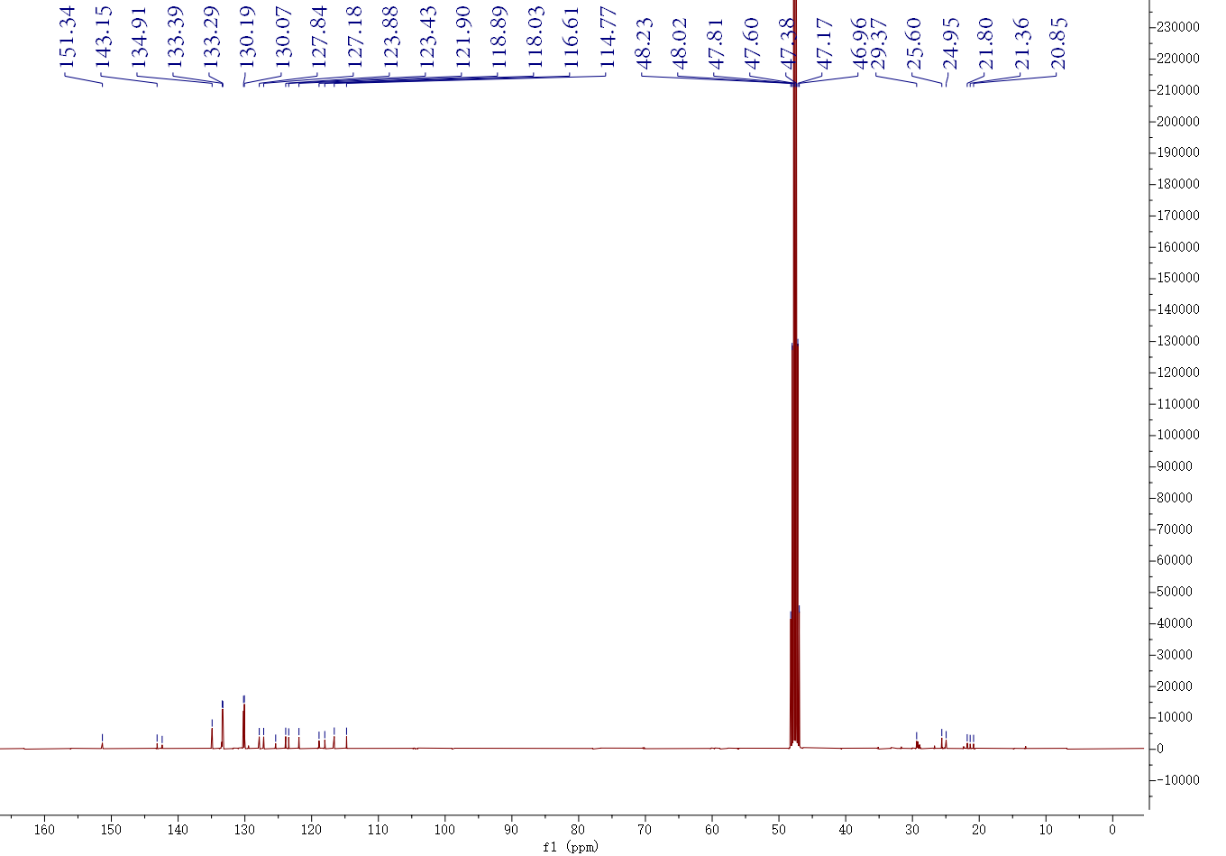
 ^13^C NMR spectrum of PTZ-Mito in CD_3_OD


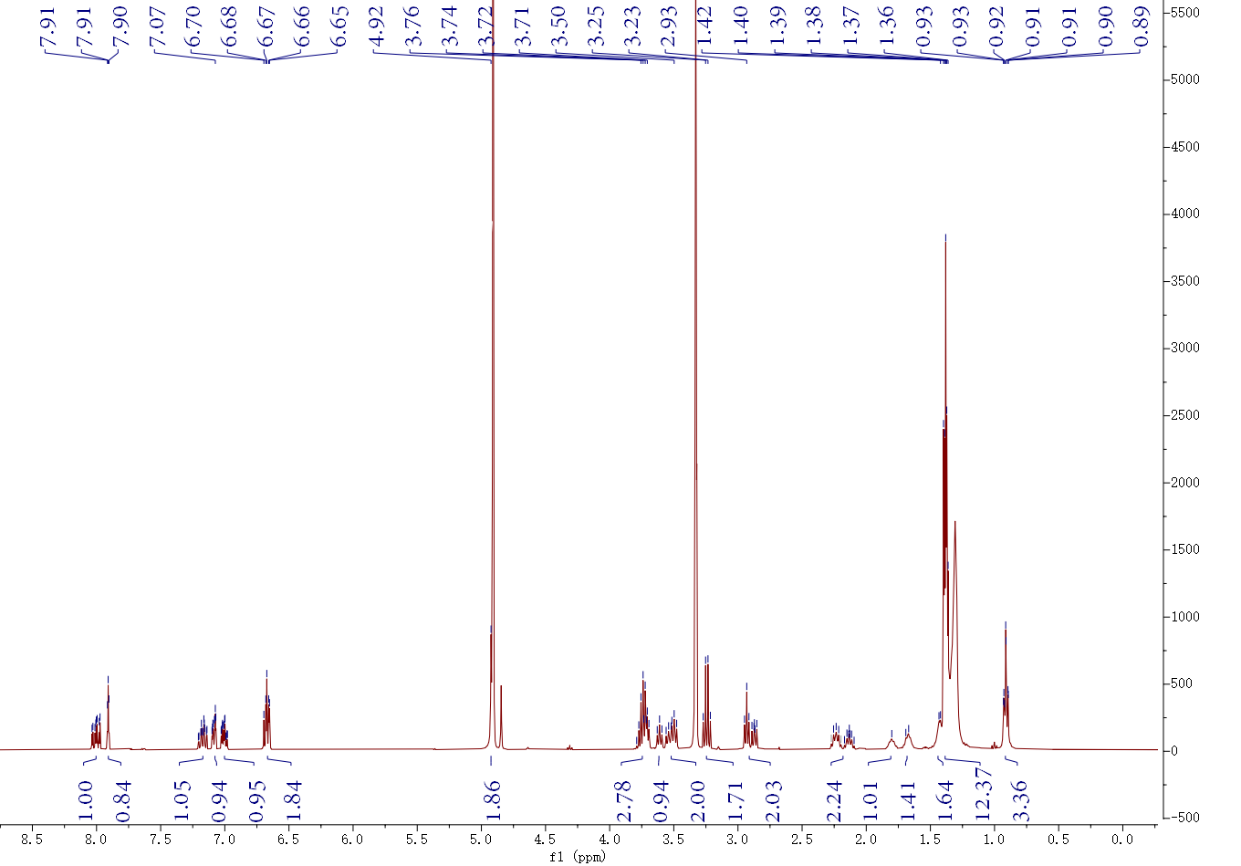


^1^H NMR spectrum of PTZ-Memb in DMSO-d6


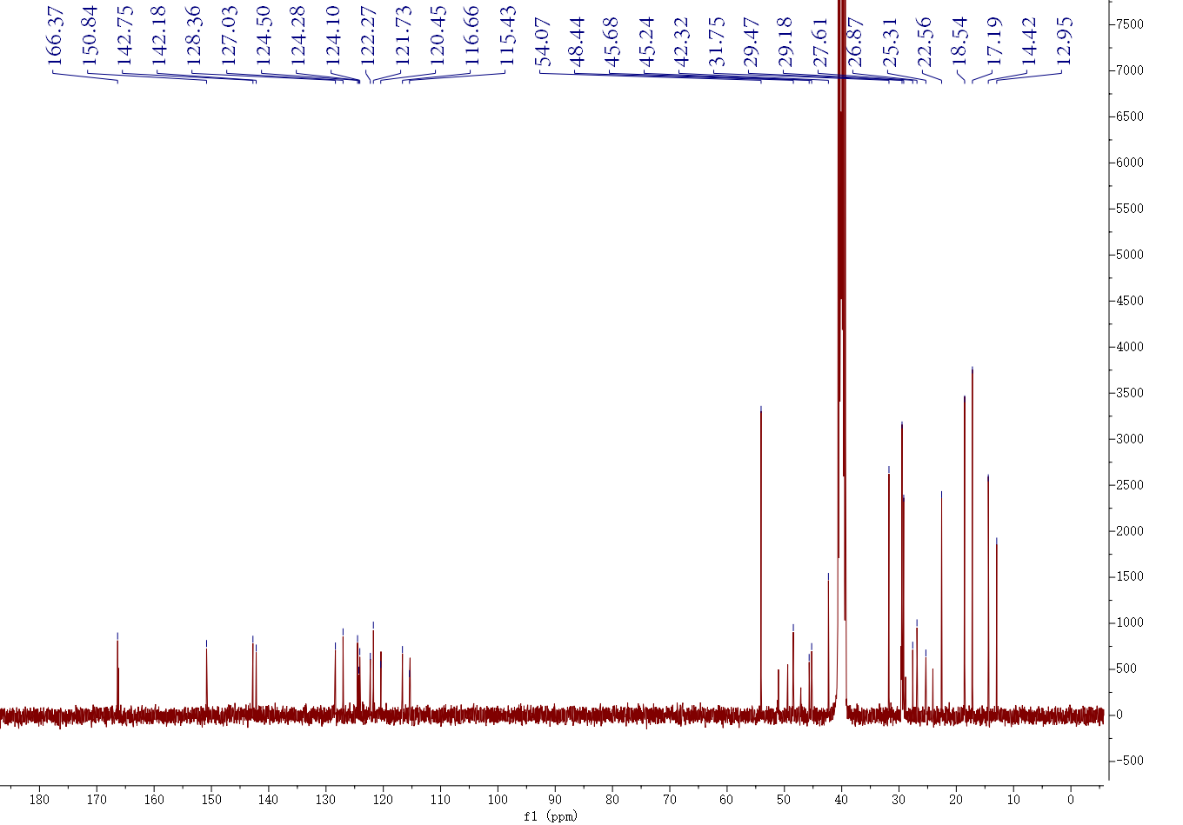


^13^C NMR spectrum of PTZ-Memb in DMSO-d6


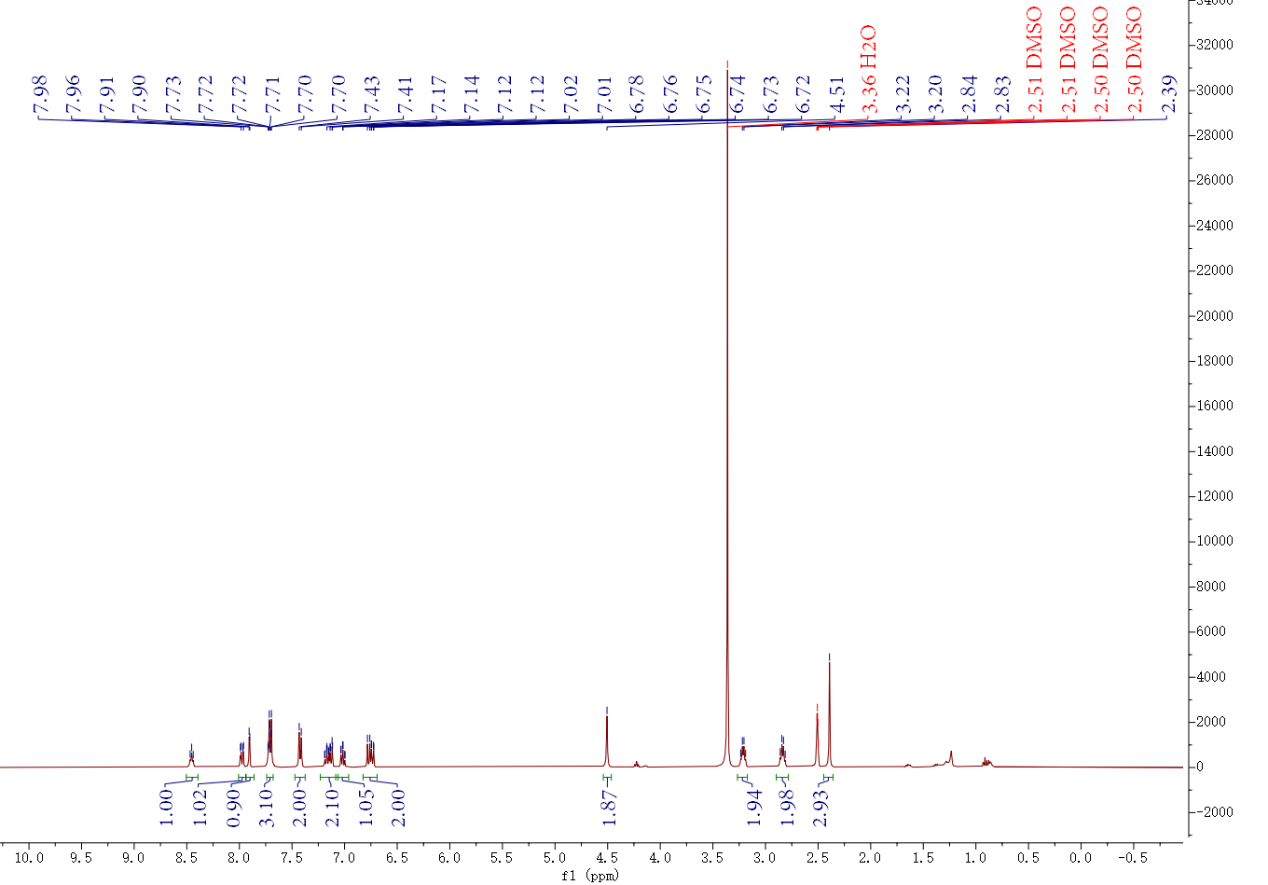


^1^H NMR spectrum of PTZ-ER in DMSO-d6


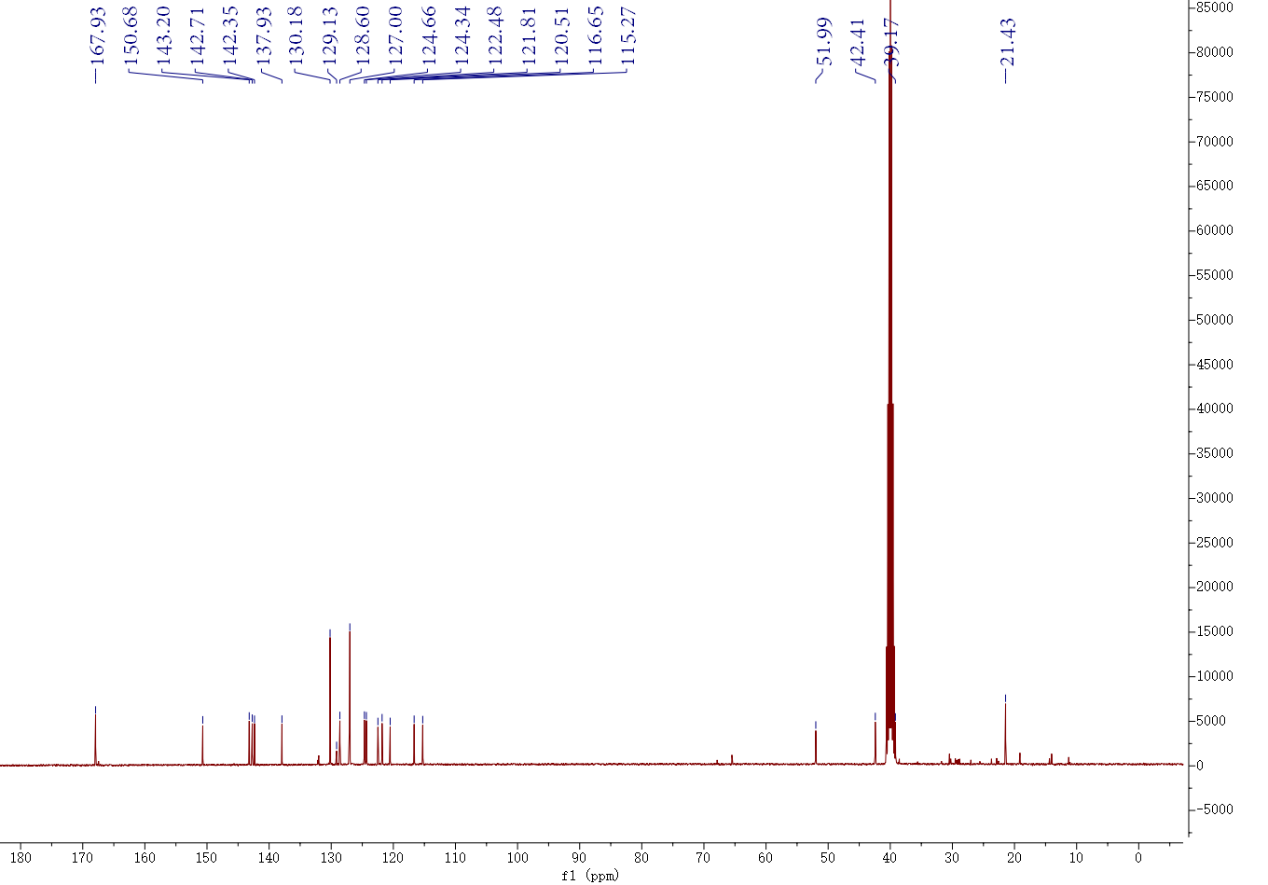
 ^13^C NMR spectrum of PTZ-ER in DMSO-d6


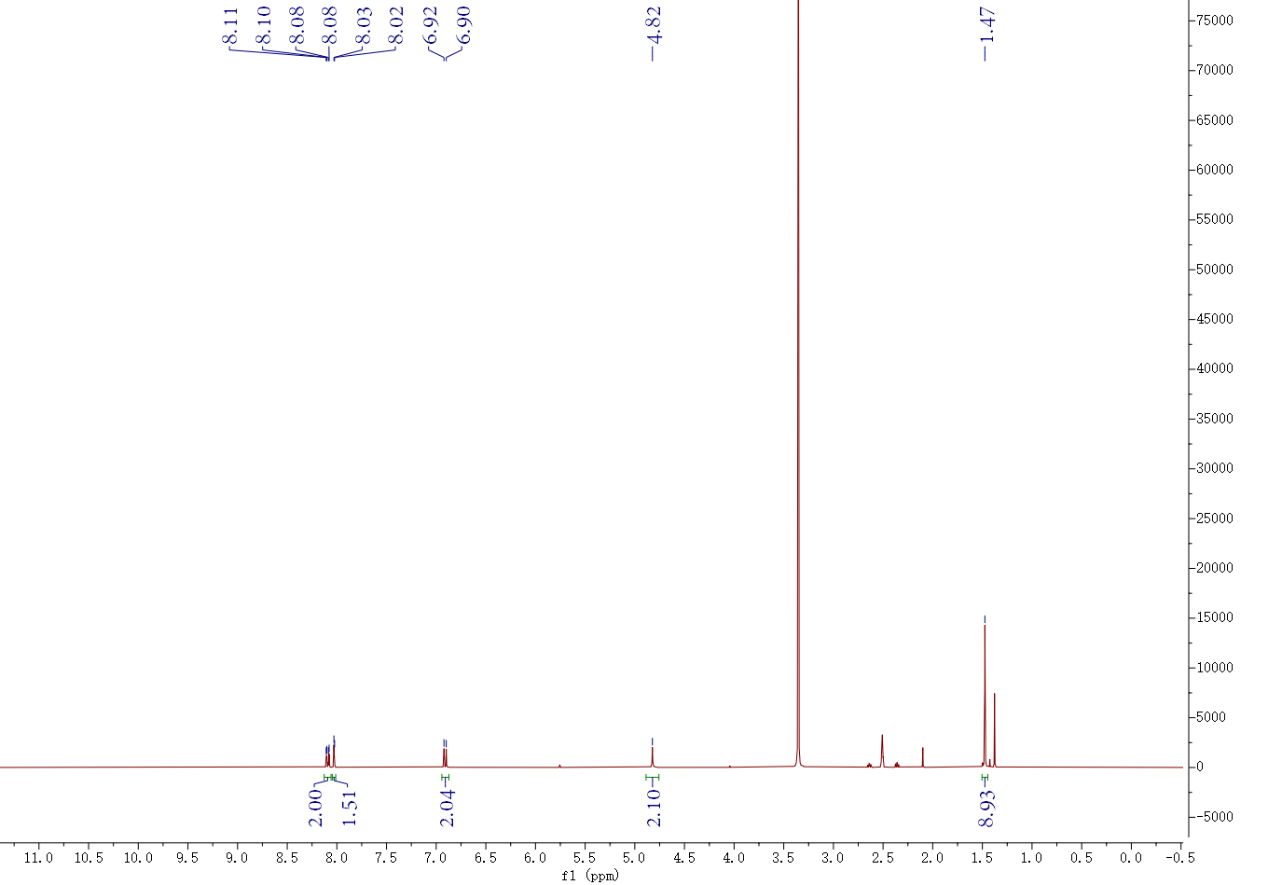


^1^H NMR spectrum of PTZ2N-Lipid (3a) in CDCl_3_


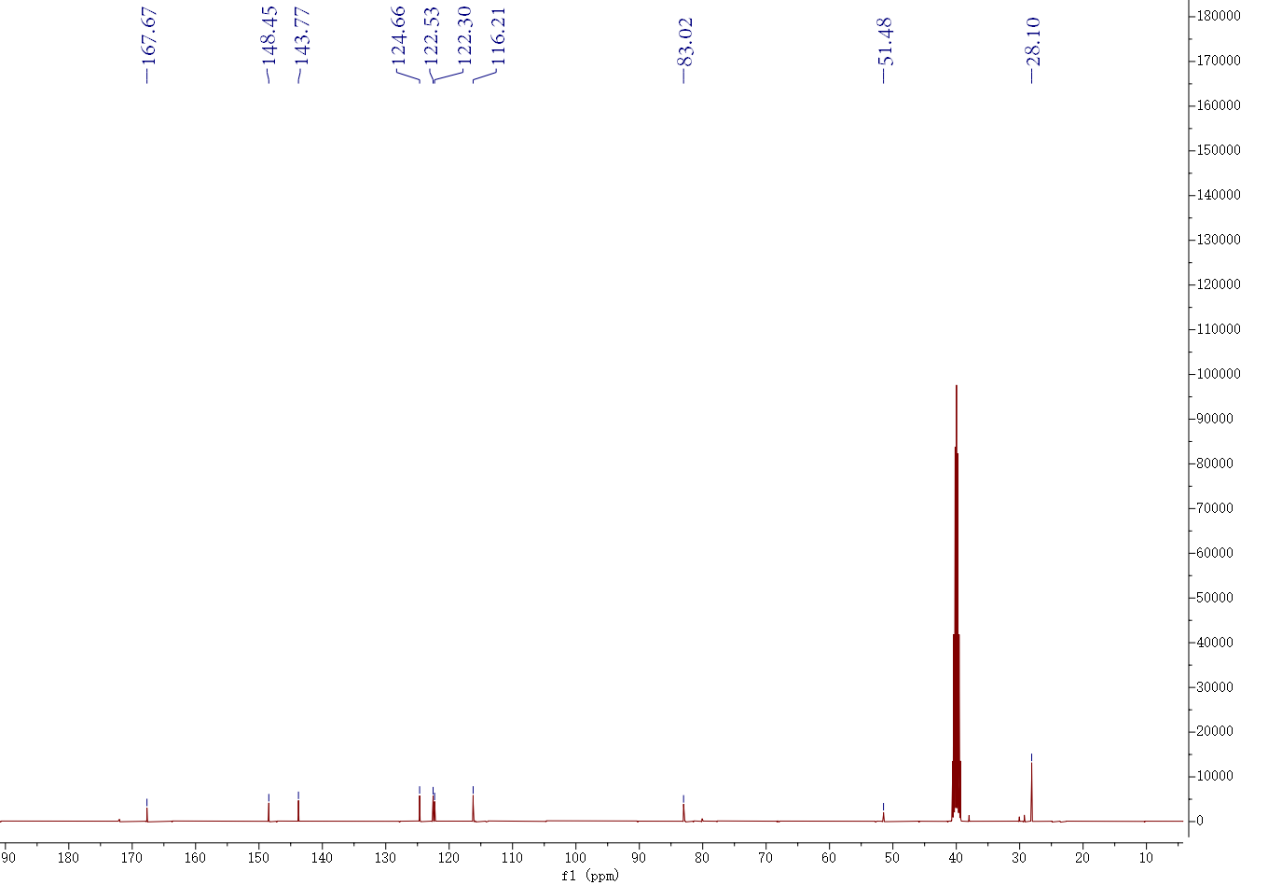
 ^13^C NMR spectrum of PTZ2N-Lipid (3a) in CDCl_3_


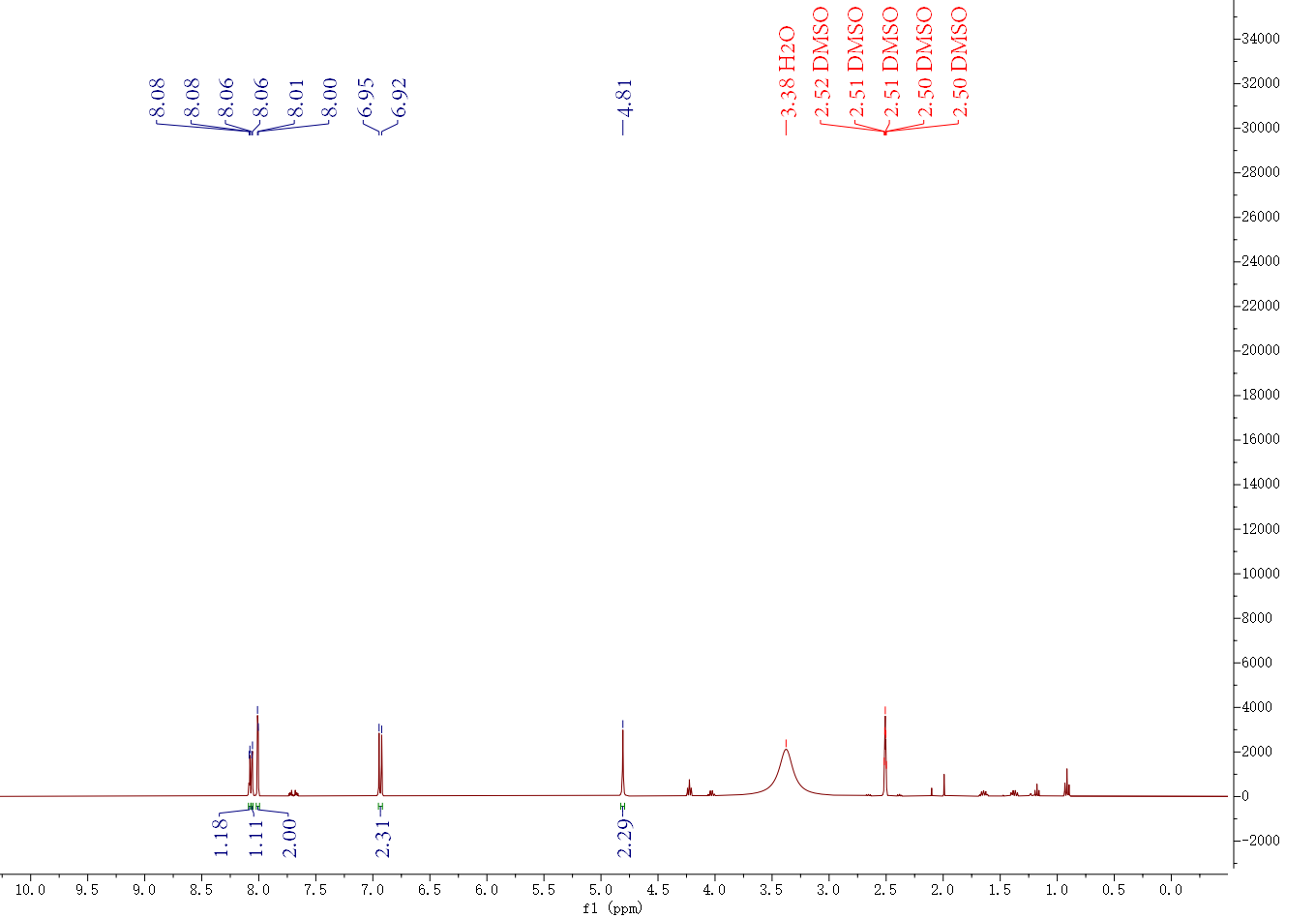


^1^H NMR spectrum of 4b in DMSO-d6


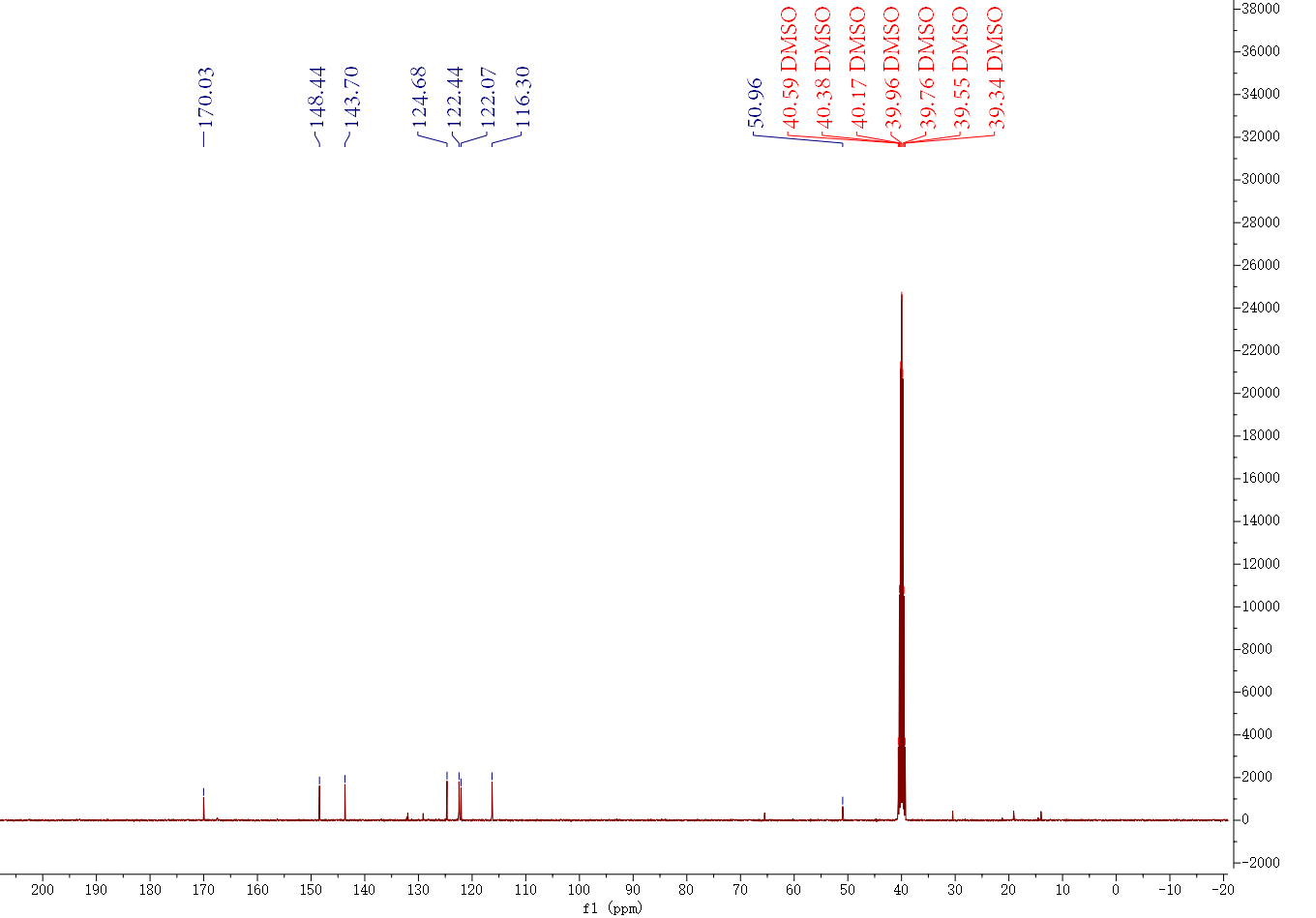


^13^C NMR spectrum of 4b in DMSO-d6


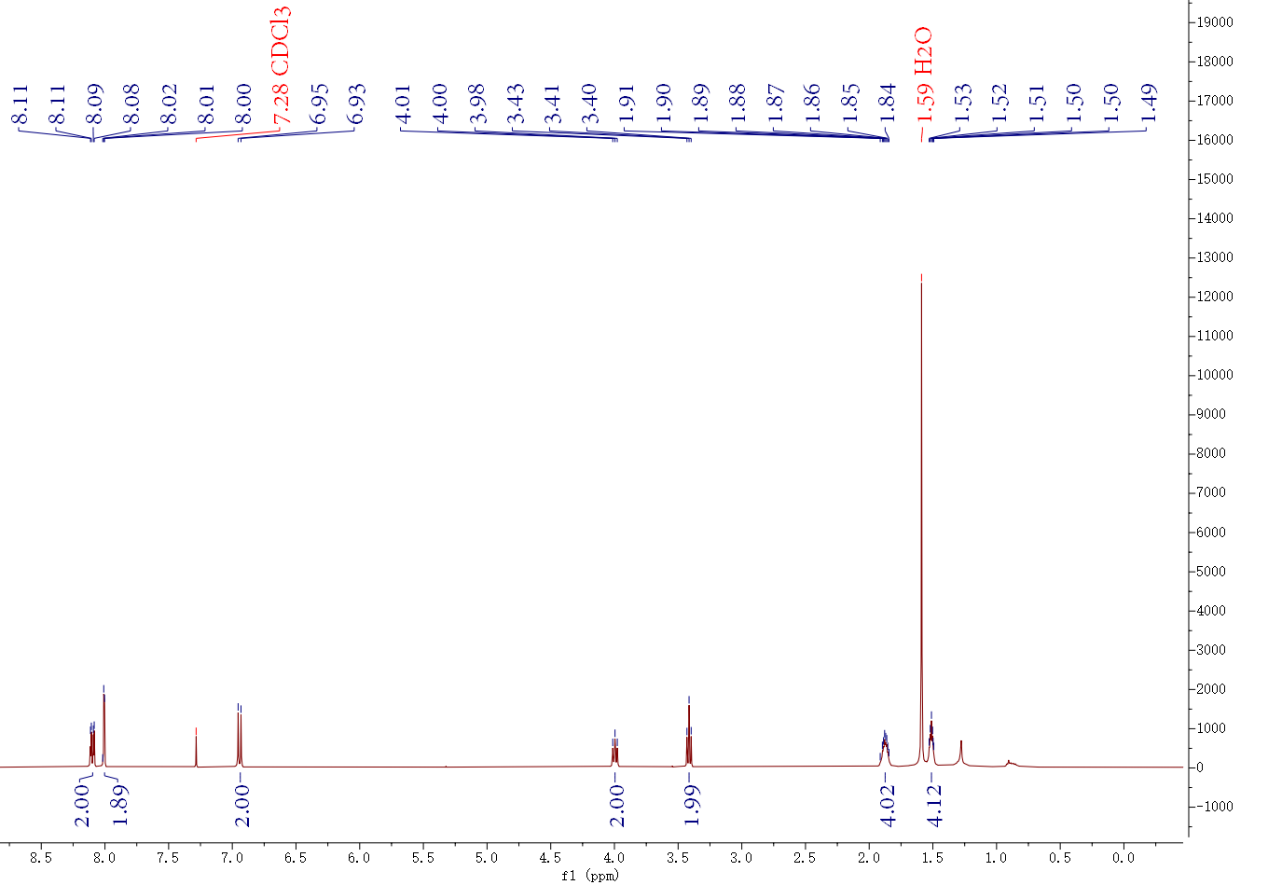


^1^H NMR spectrum of 2b in CDCl_3_


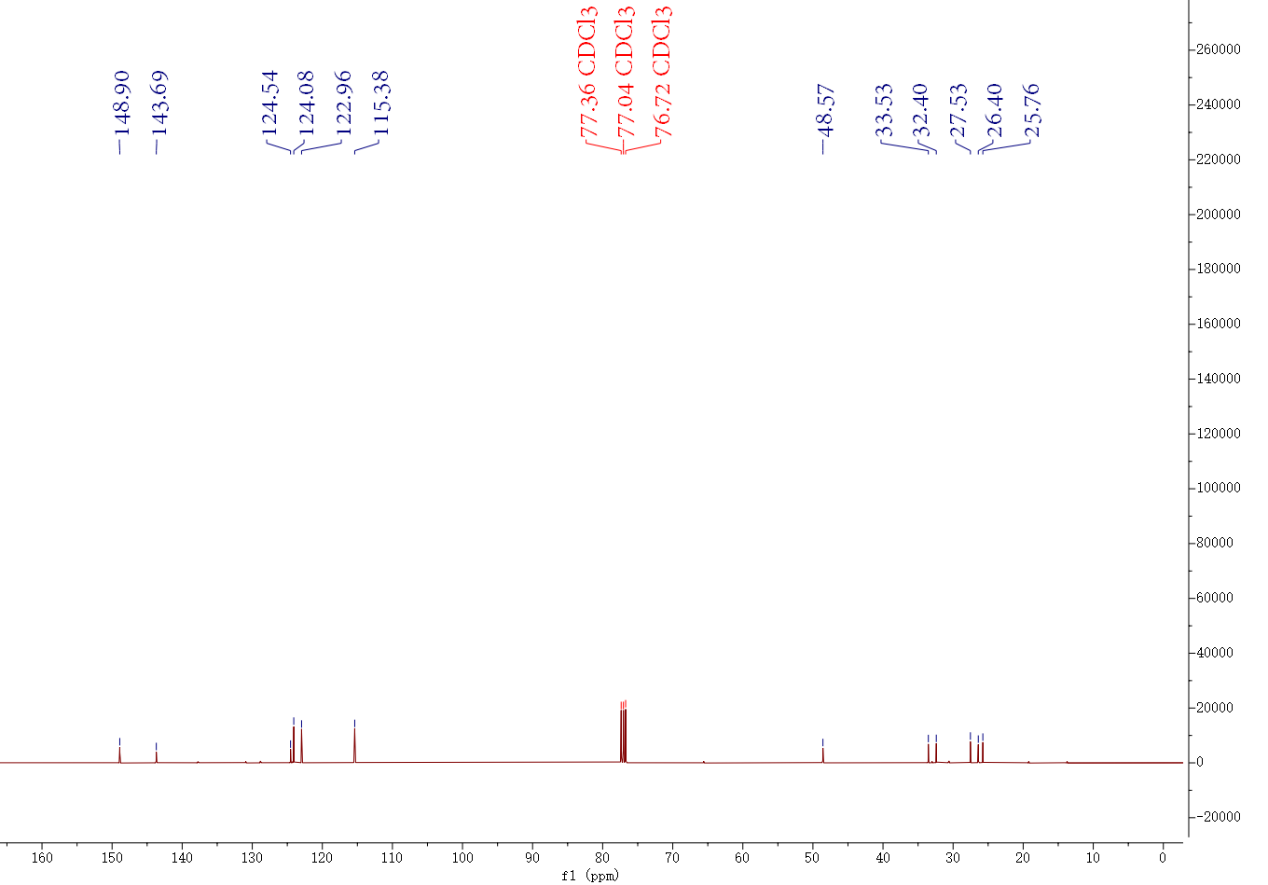
 ^13^C NMR spectrum of 2b in CDCl_3_


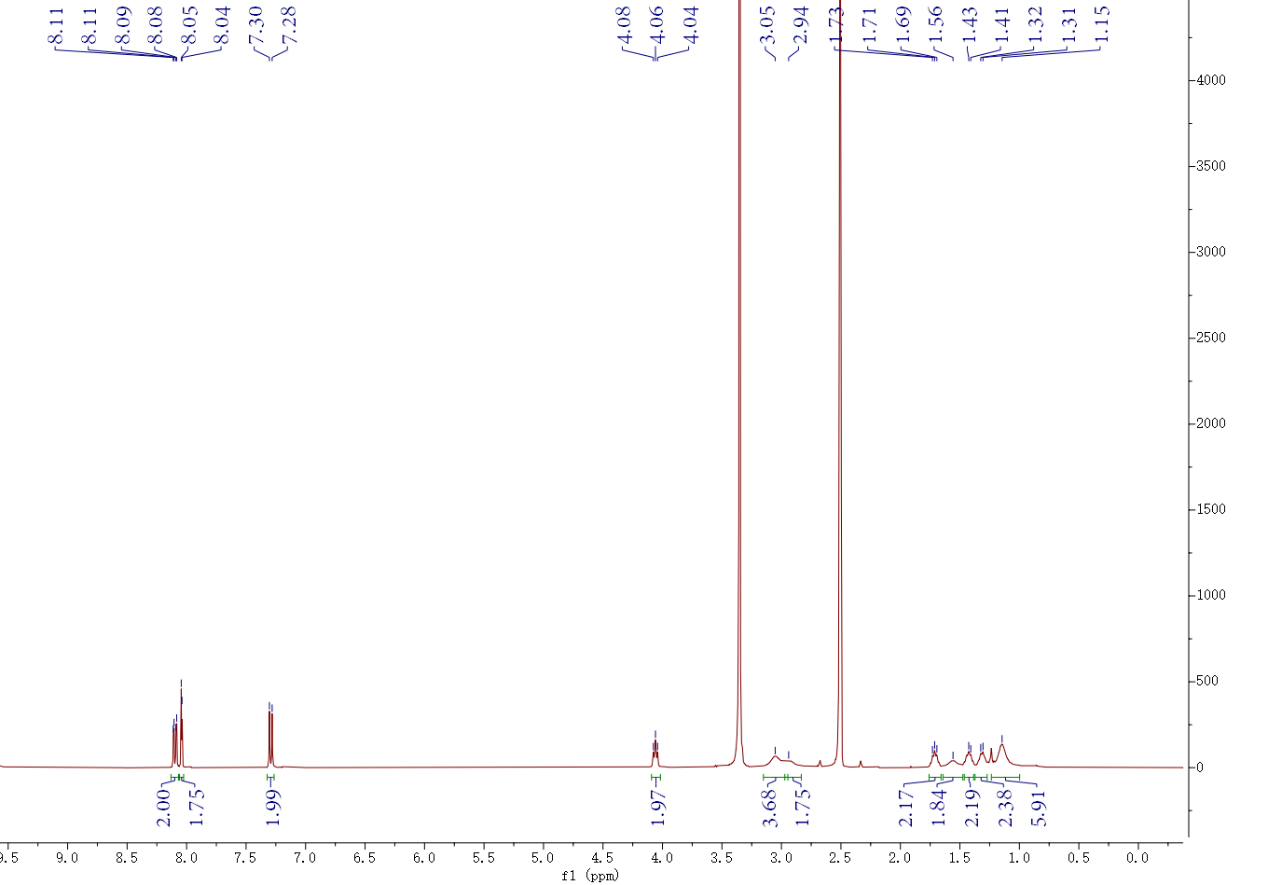
 ^1^H NMR spectrum of PTZ2N-Lyso in CDCl_3_


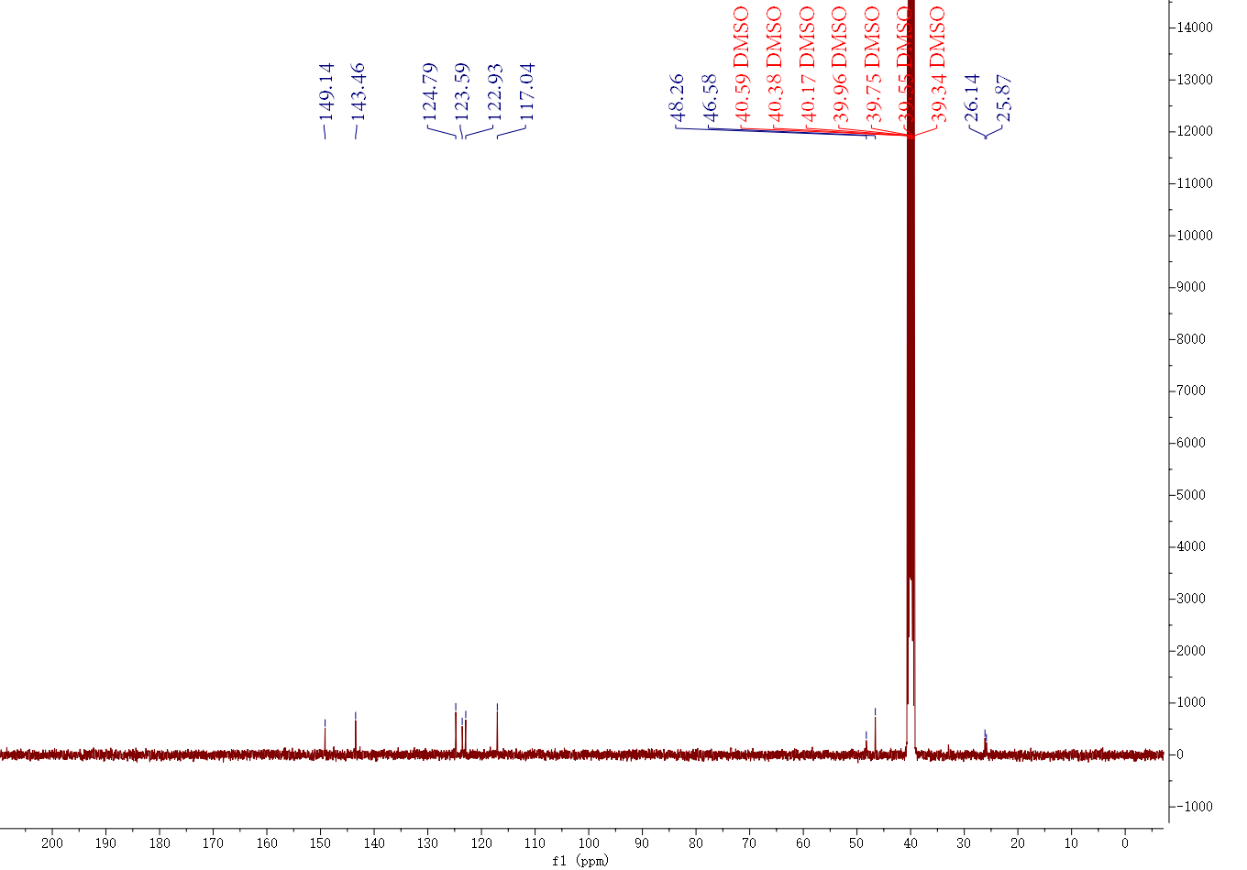


^13^C NMR spectrum of PTZ2N-Lyso in CDCl_3_


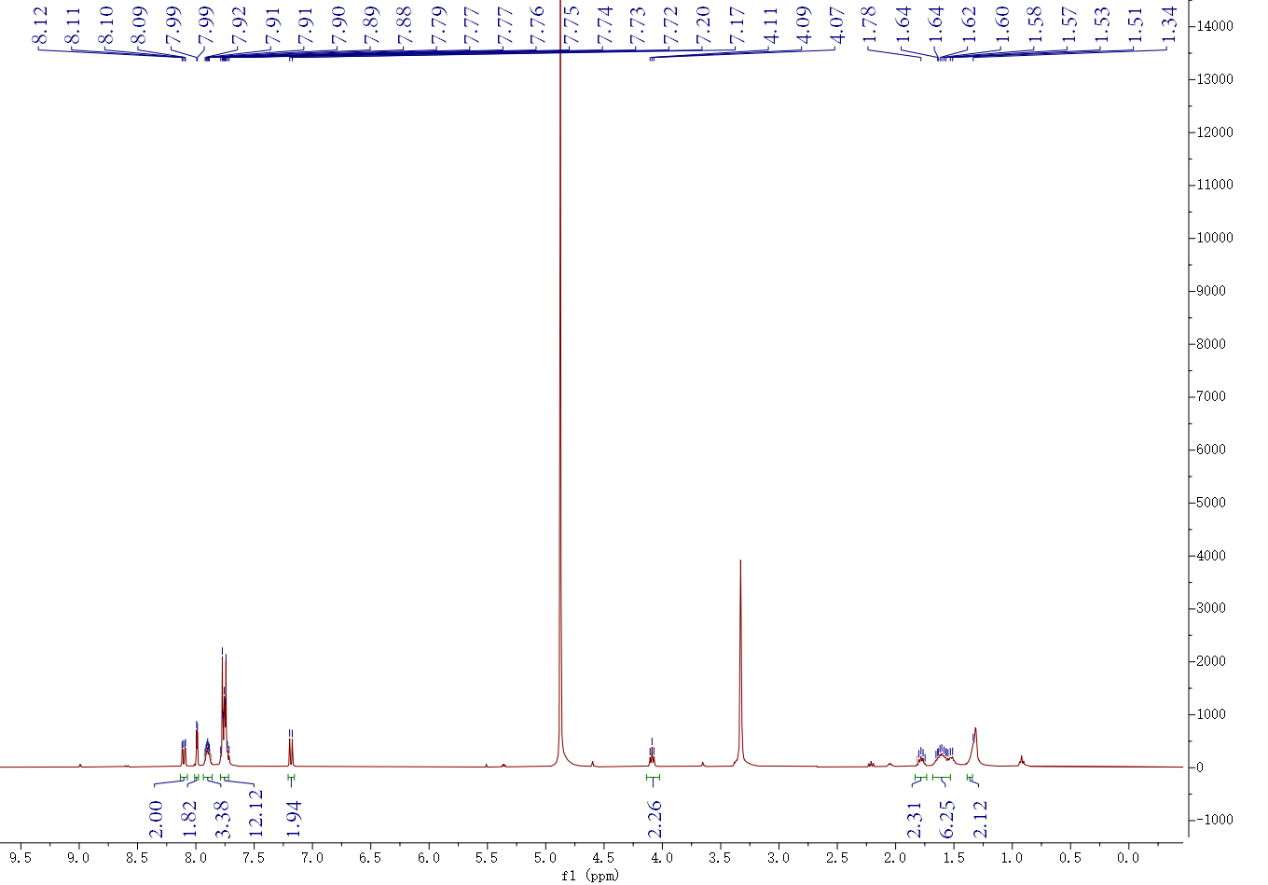


^1^H NMR spectrum of PTZ2N-Mito in CD_3_OD


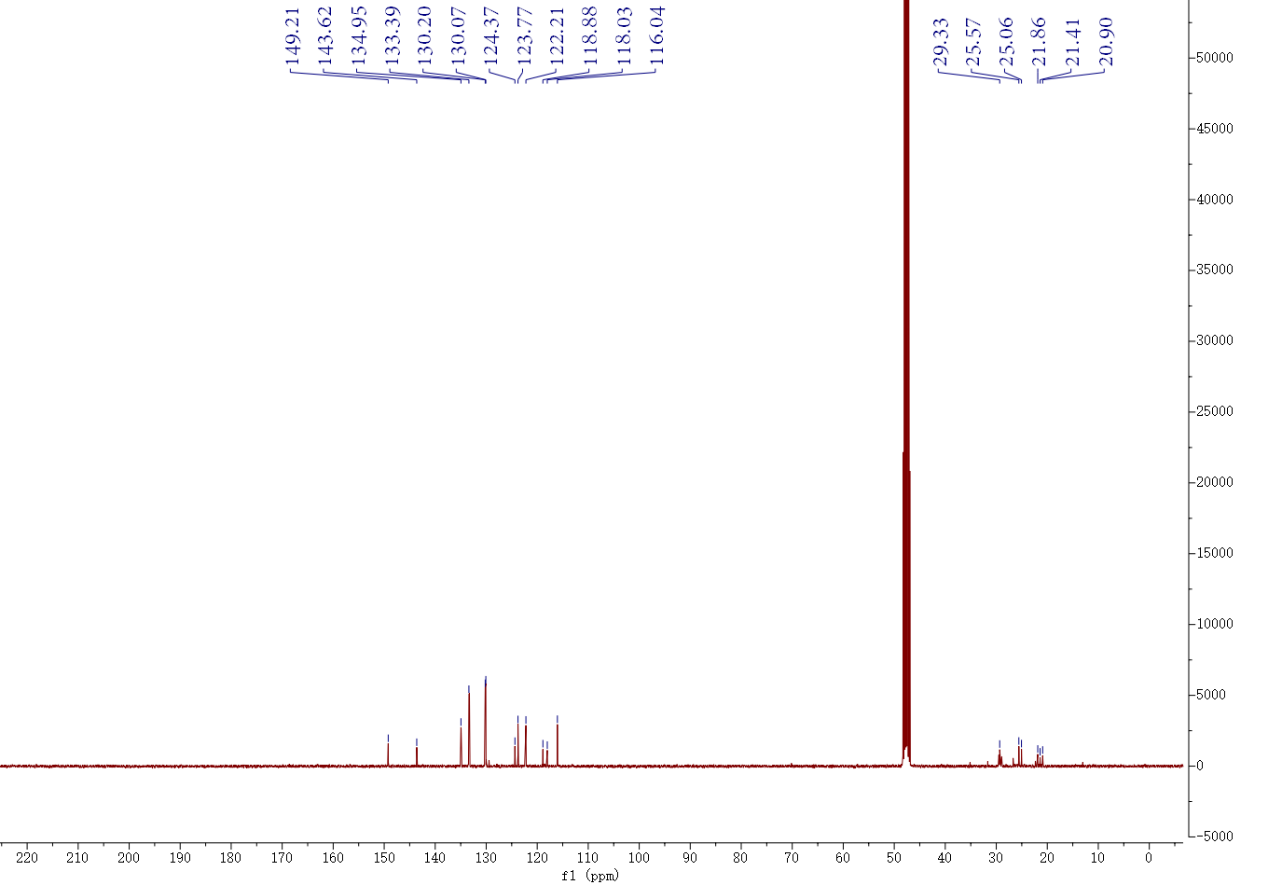
 ^13^C NMR spectrum of PTZ2N-Mito in CD_3_OD


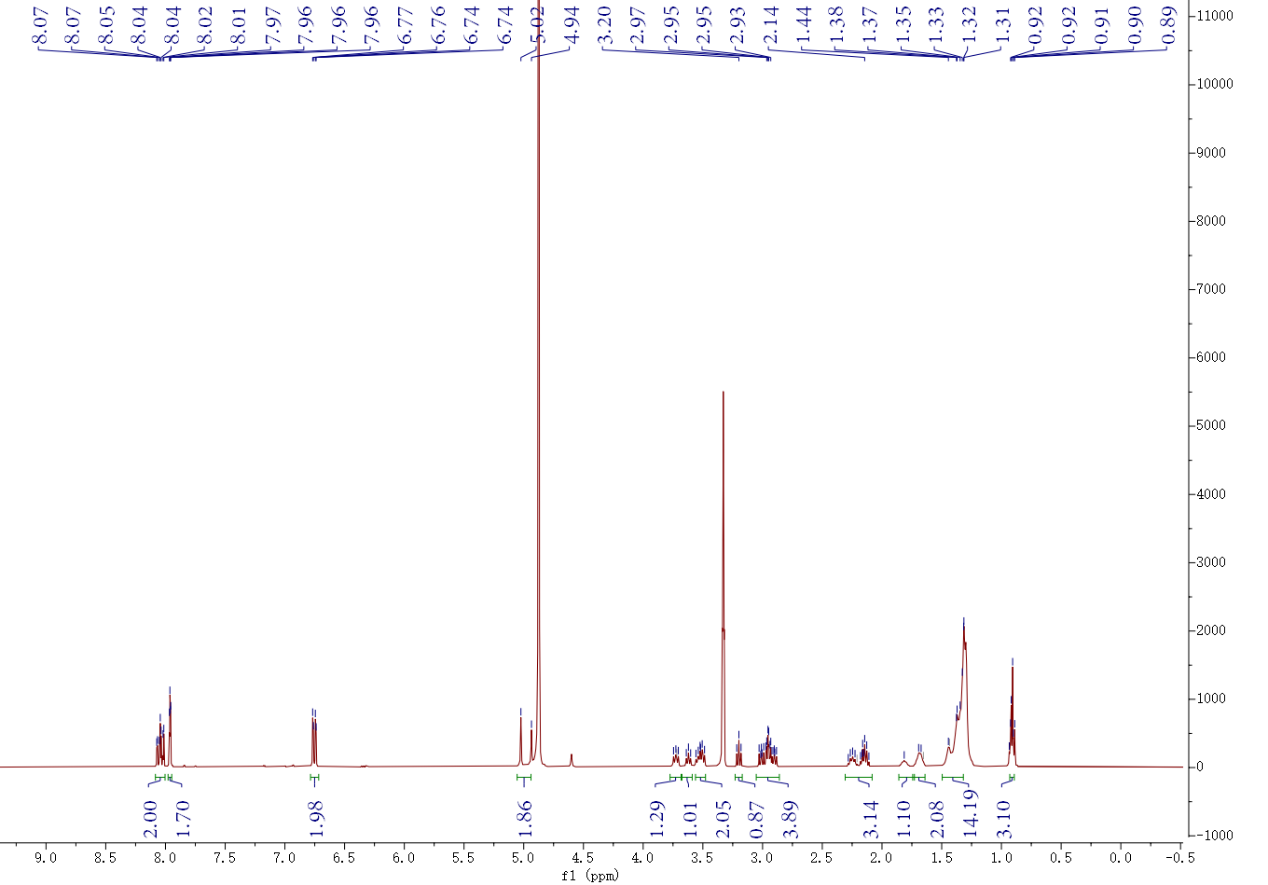


^1^H NMR spectrum of PTZ2N-Memb in DMSO-d6


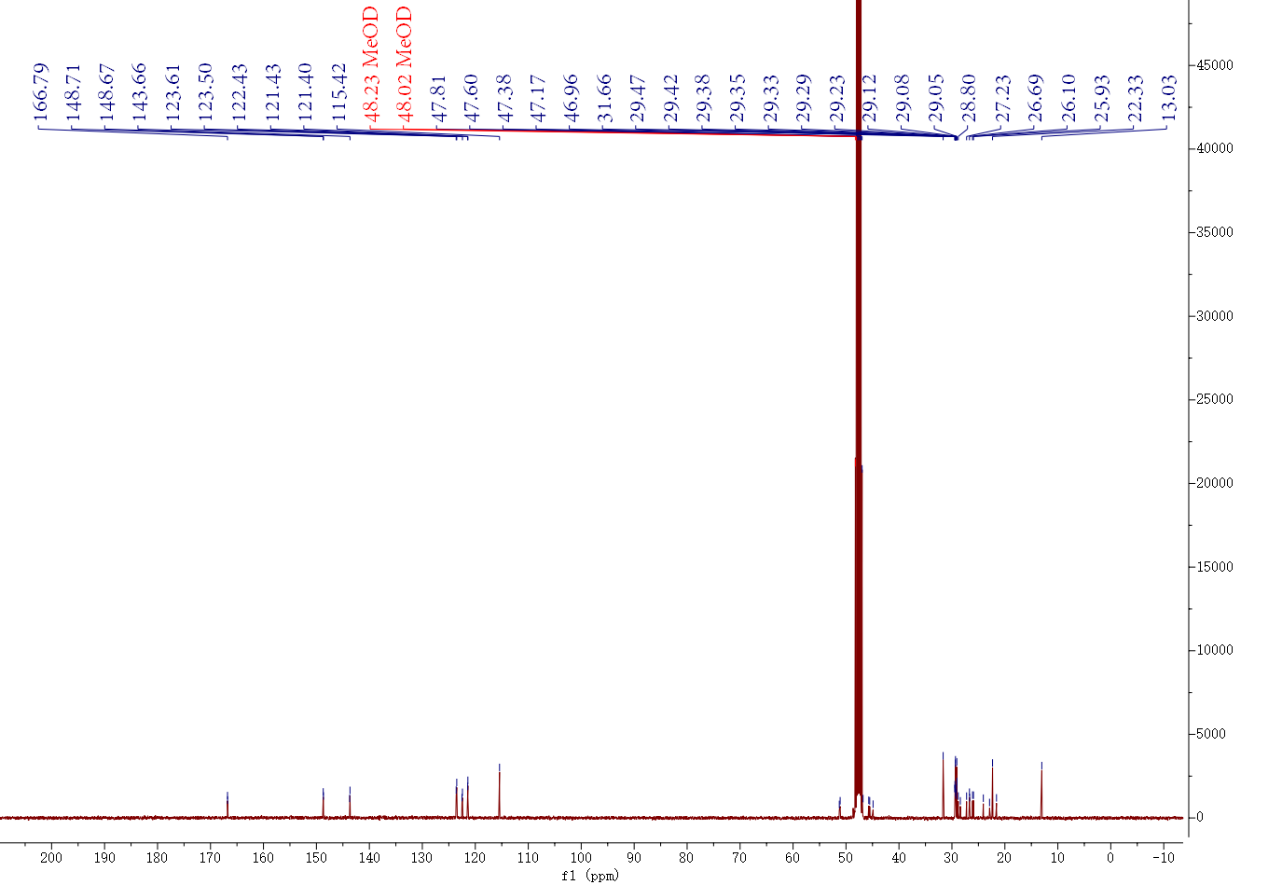
 ^13^C NMR spectrum of PTZ2N-Memb in DMSO-d6


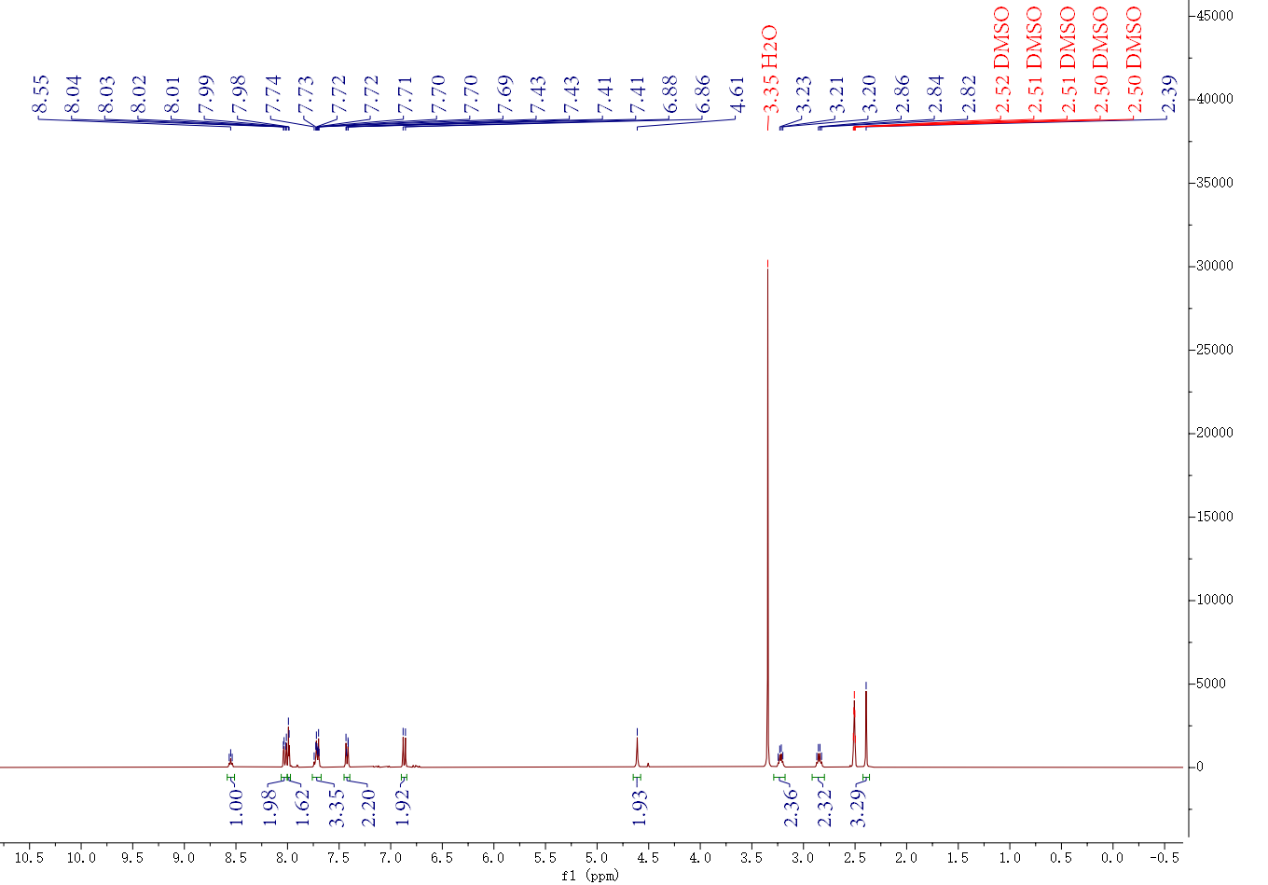
 ^1^H NMR spectrum of PTZ2N-ER in DMSO-d6


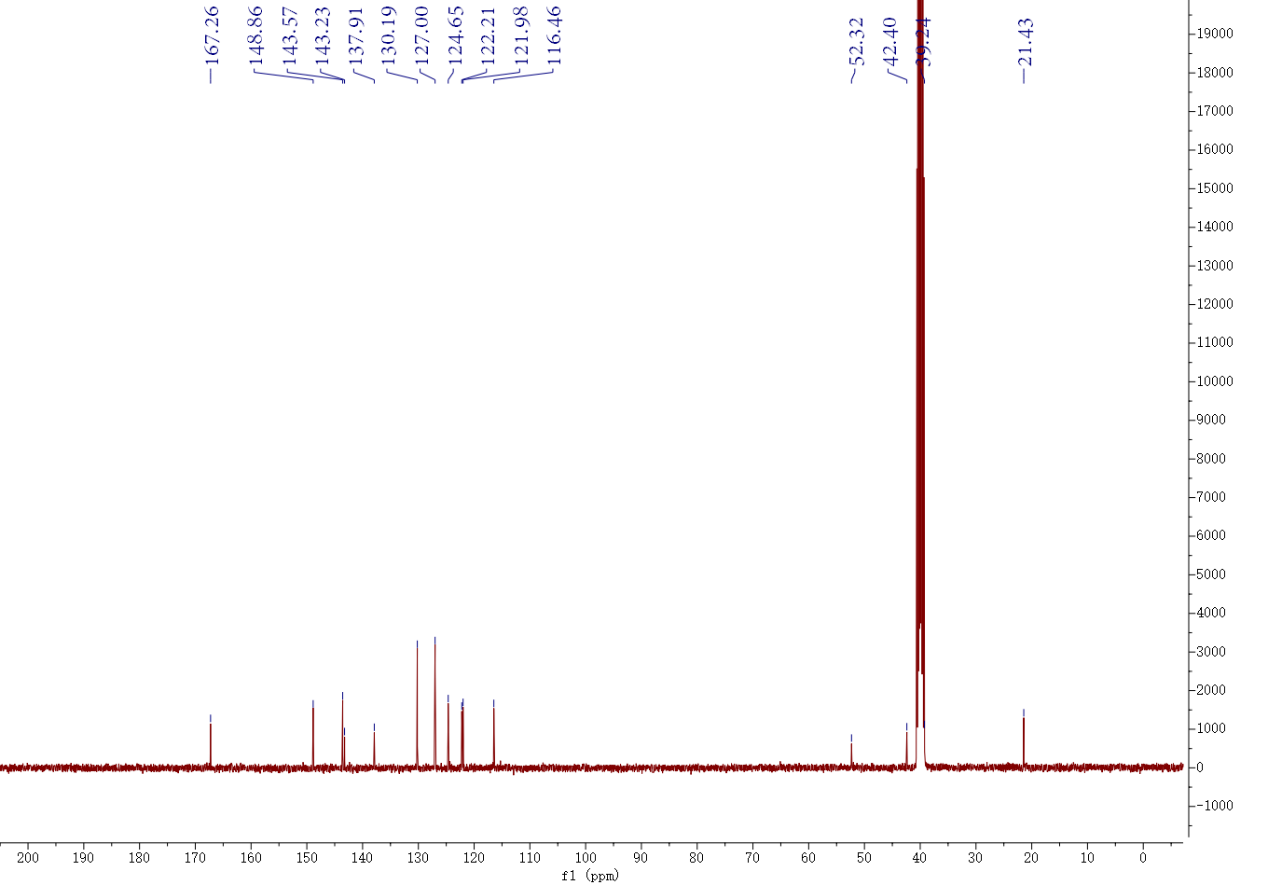


^13^C NMR spectrum of PTZ2N-ER in DMSO-d6


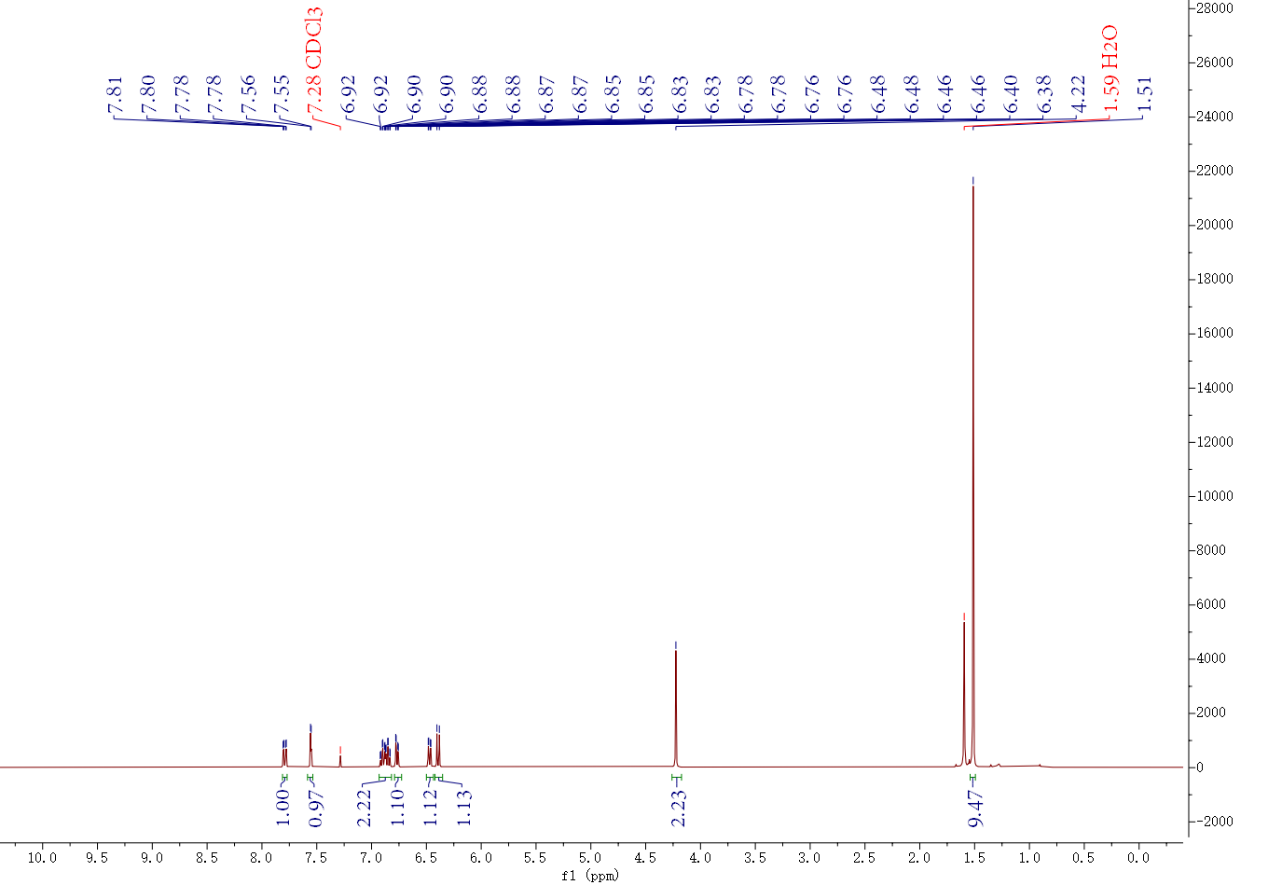


^1^H NMR spectrum of PXZ-Lipid (3c) in CDCl_3_


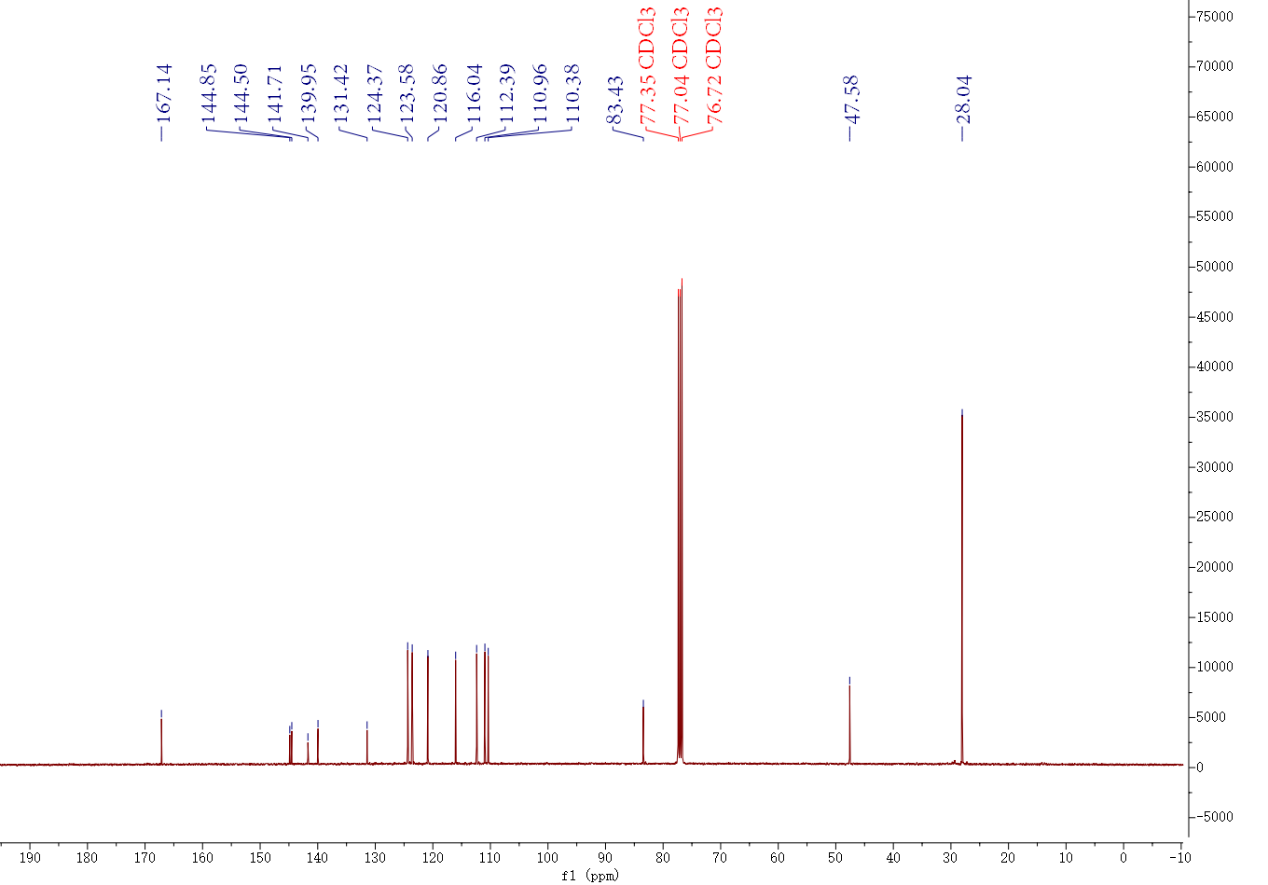
 ^13^C NMR spectrum of PXZ-Lipid (3c) in CDCl_3_


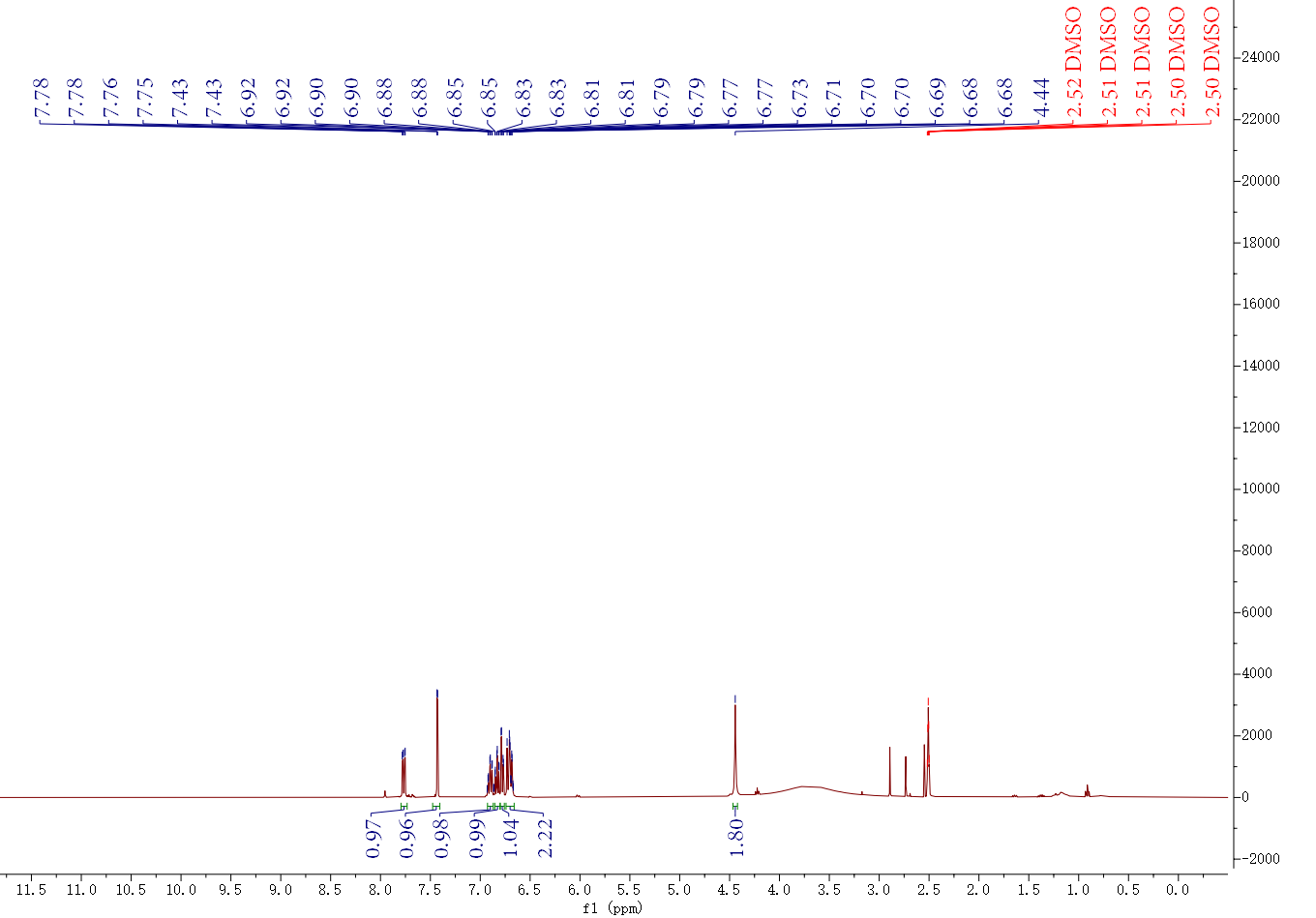


^1^H NMR spectrum of 4c in DMSO-d6


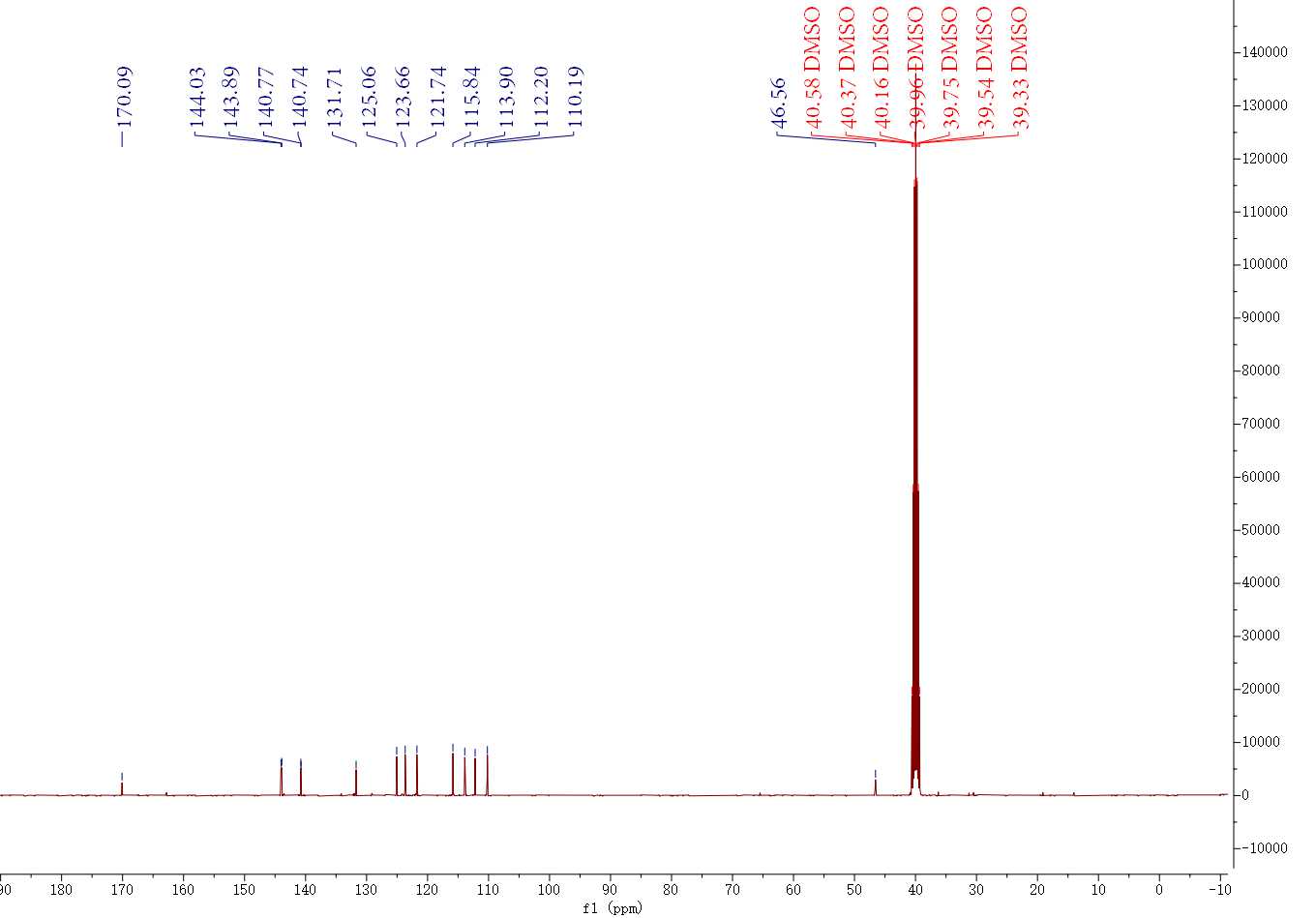


^13^C NMR spectrum of 4c in DMSO-d6


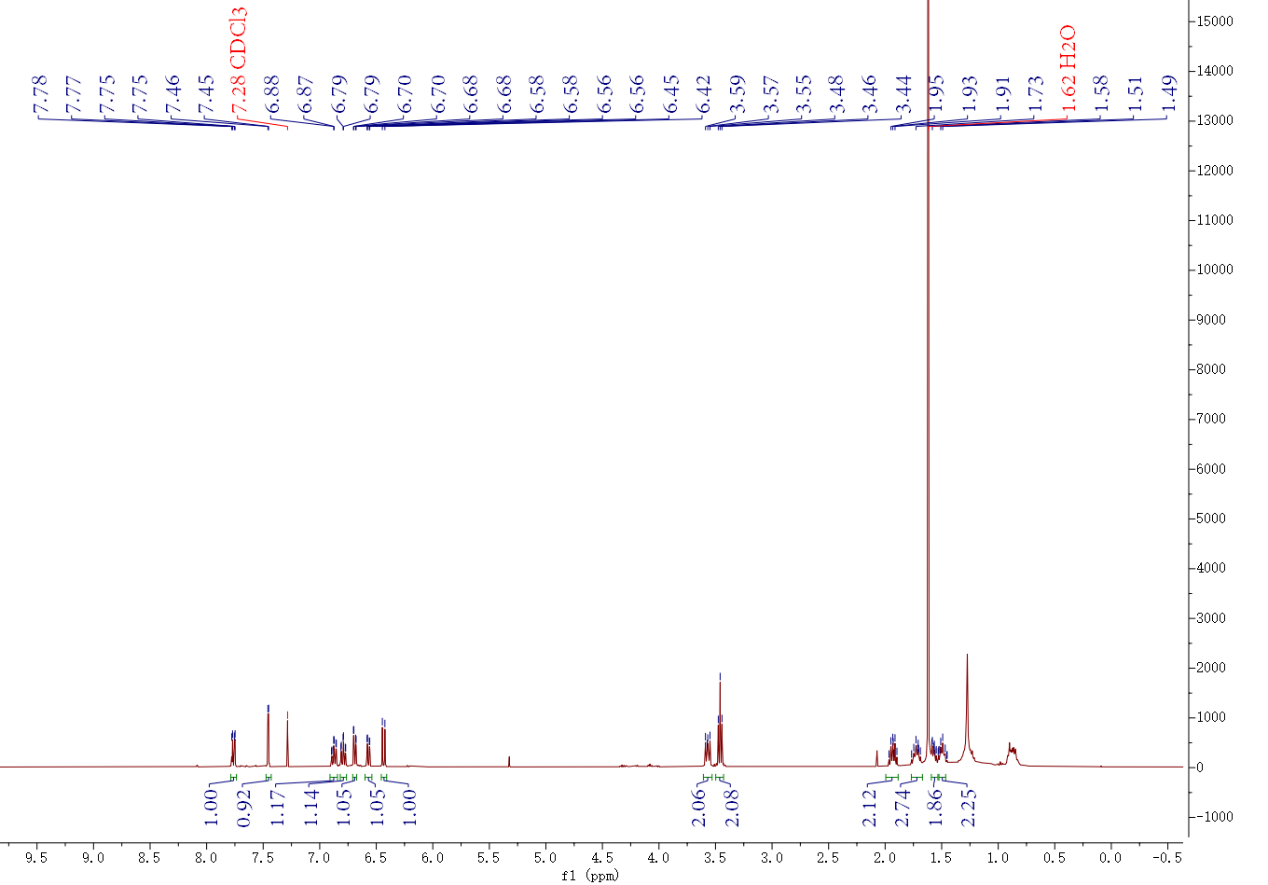


^1^H NMR spectrum of 2c in CDCl_3_


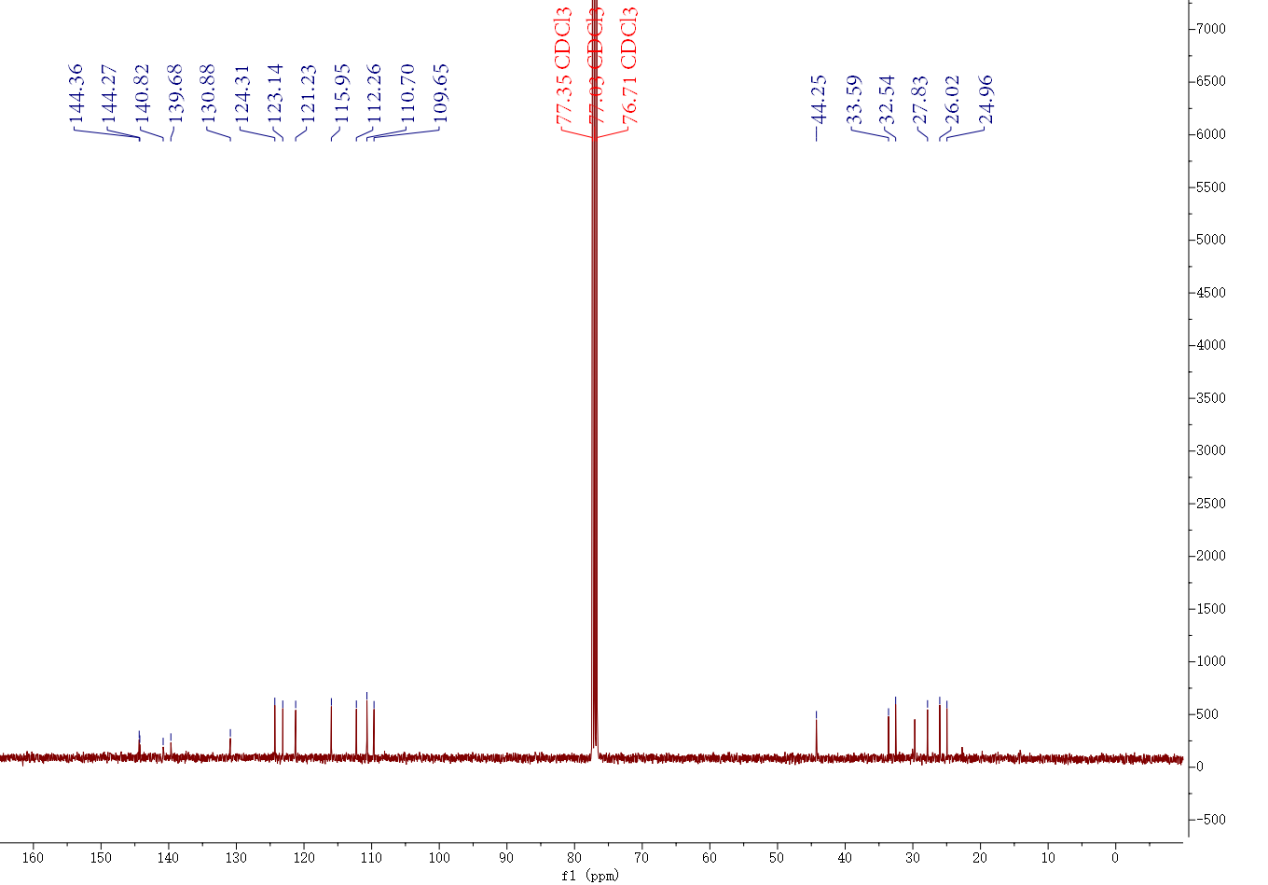
 ^13^C NMR spectrum of 2c in CDCl_3_


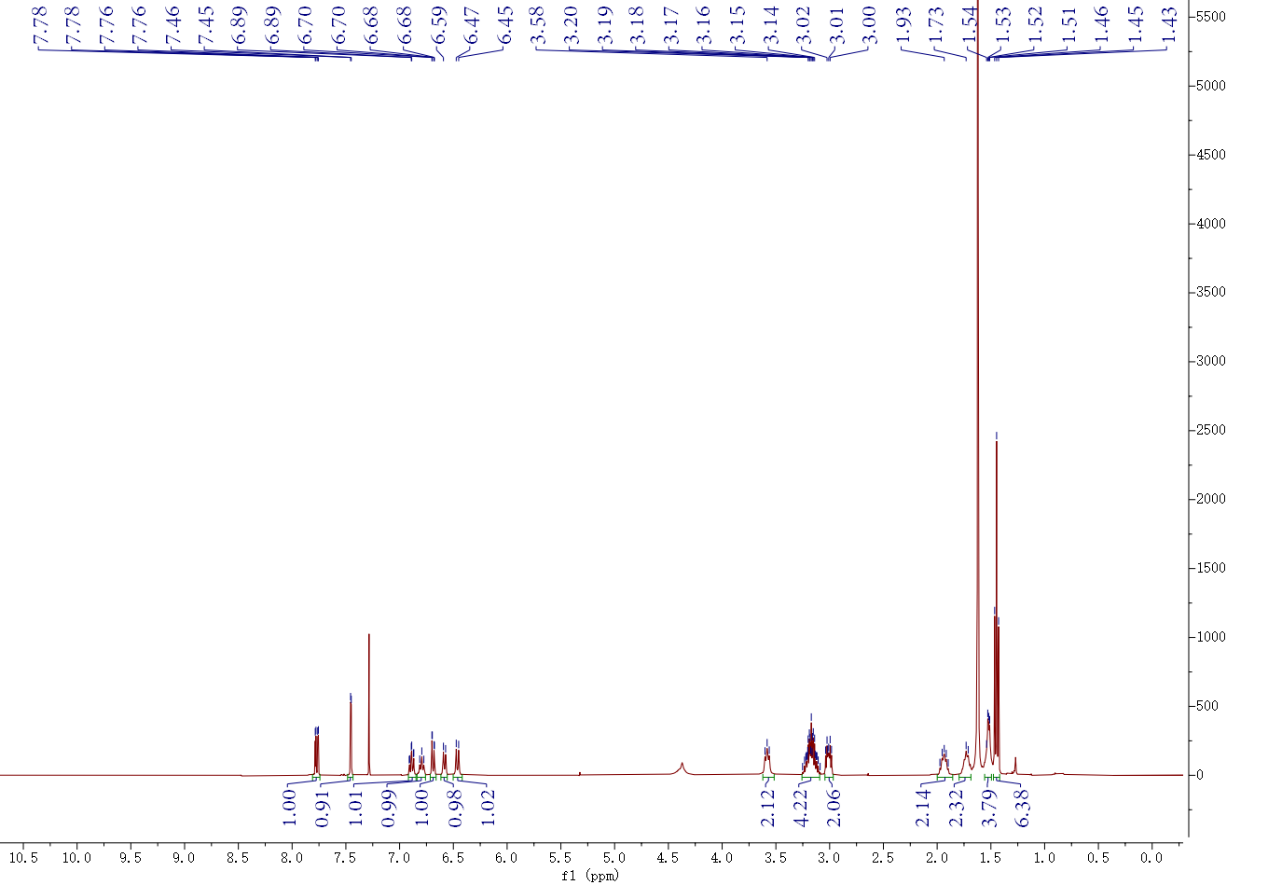
 ^1^H NMR spectrum of PXZ-Lyso in CDCl_3_

_
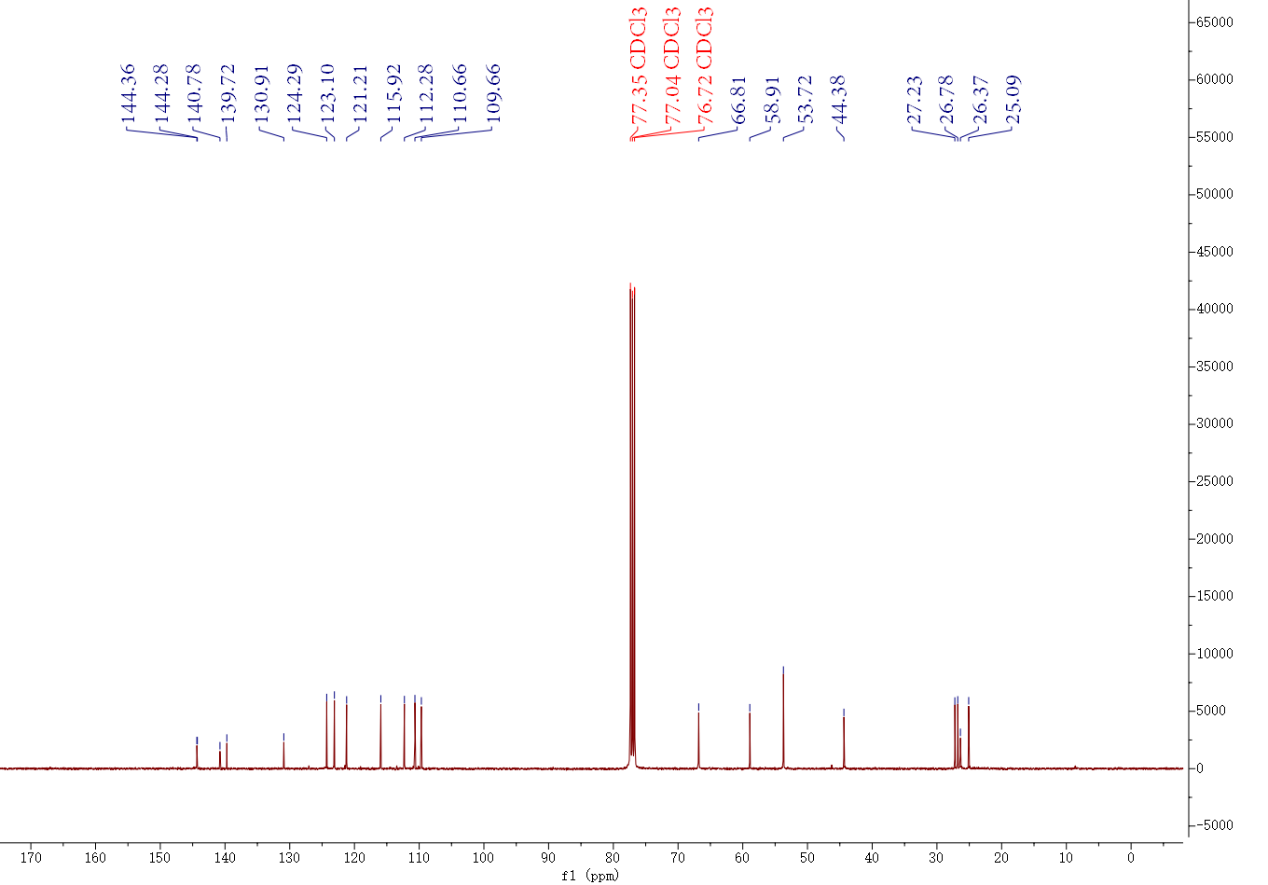
_

^13^C NMR spectrum of PXZ-Lyso in CDCl_3_


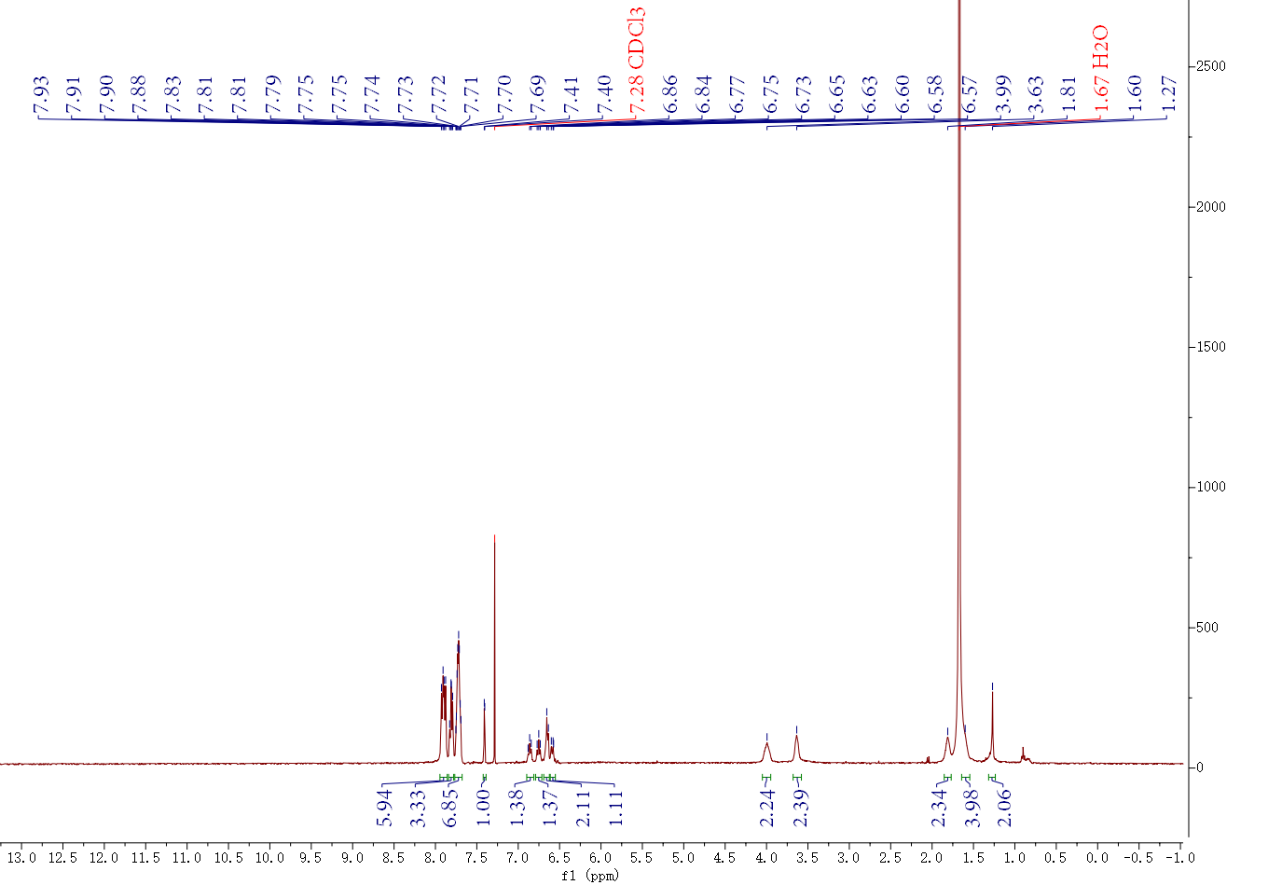
 ^1^H NMR spectrum of PXZ-Mito in CDCl_3_


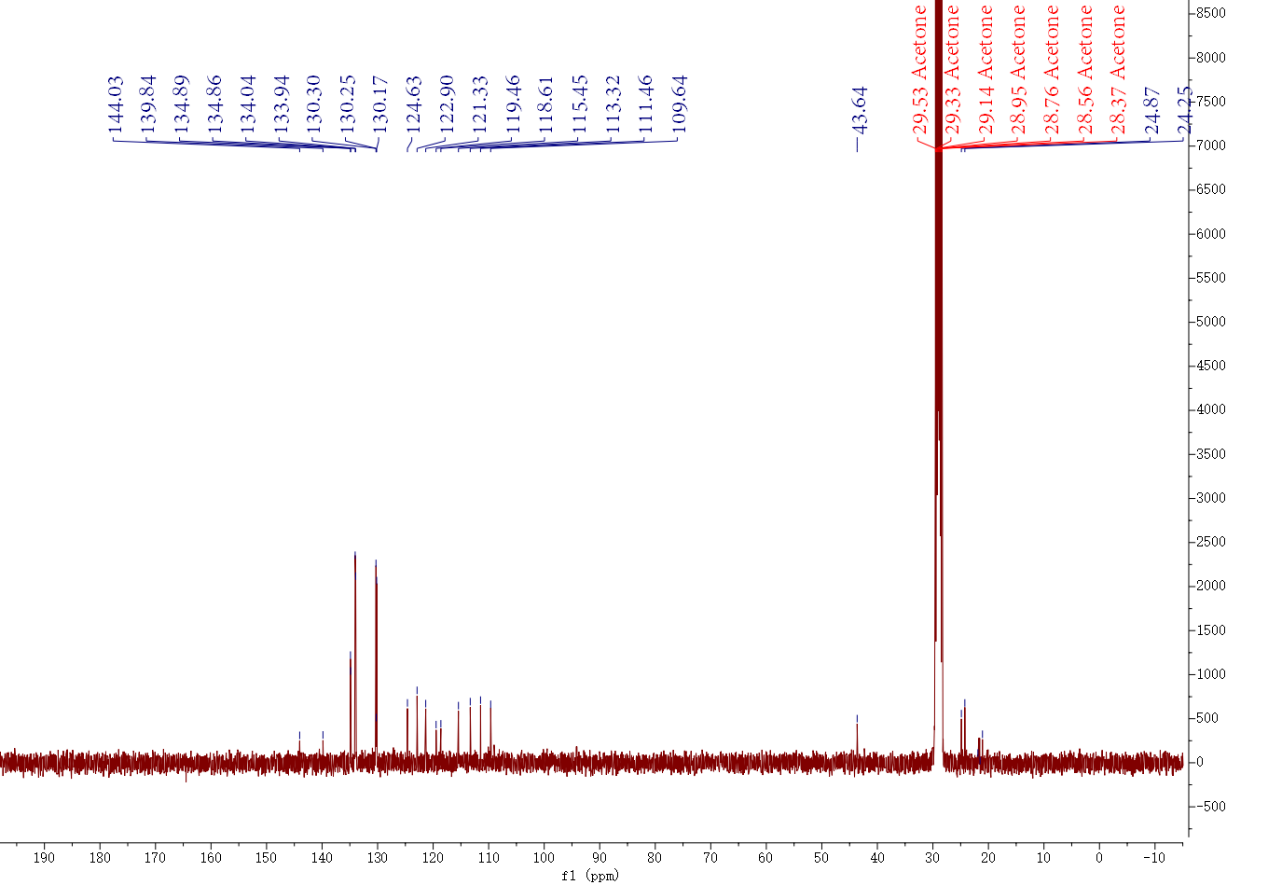


^13^C NMR spectrum of PXZ-Mito in Acetone-*d_6_*


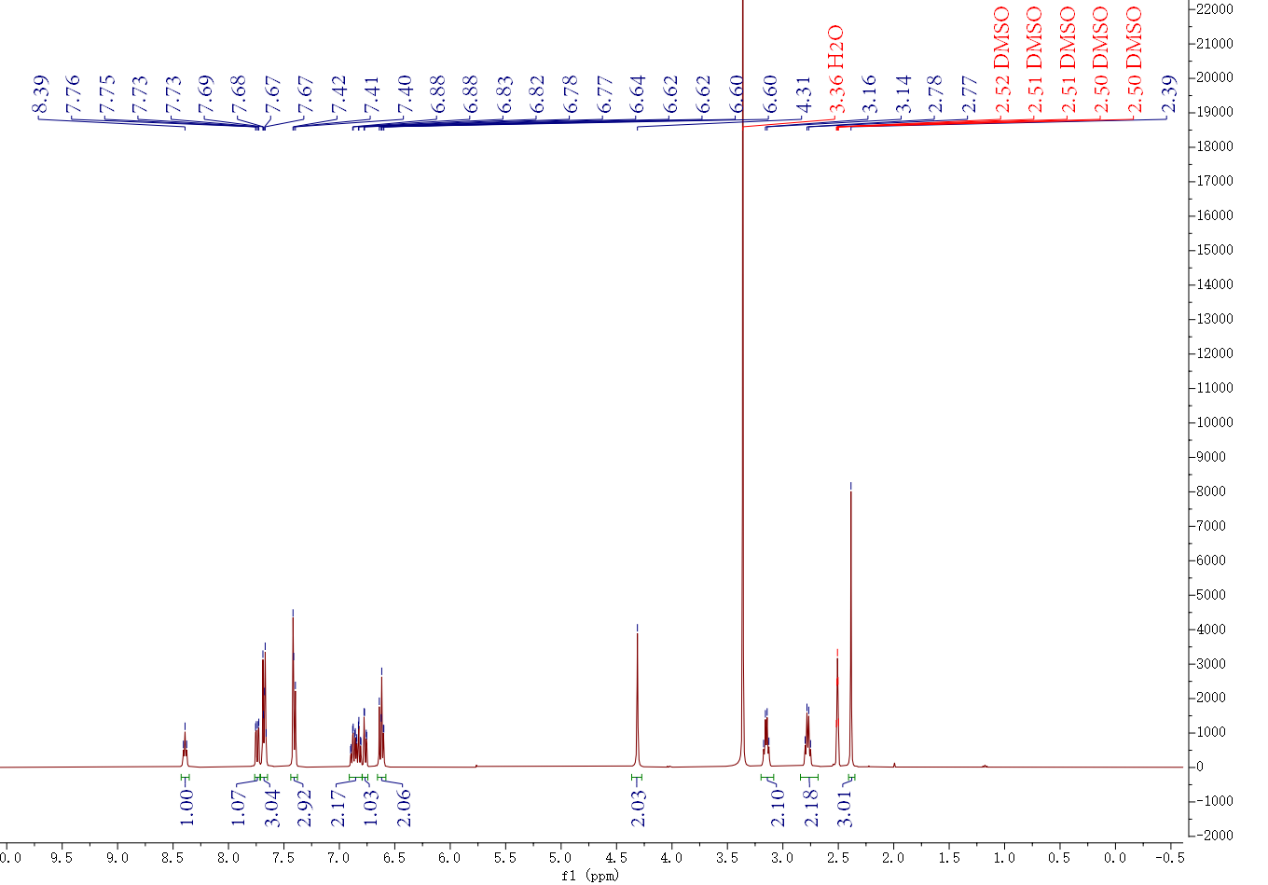


^1^H NMR spectrum of PXZ-ER in DMSO-d6


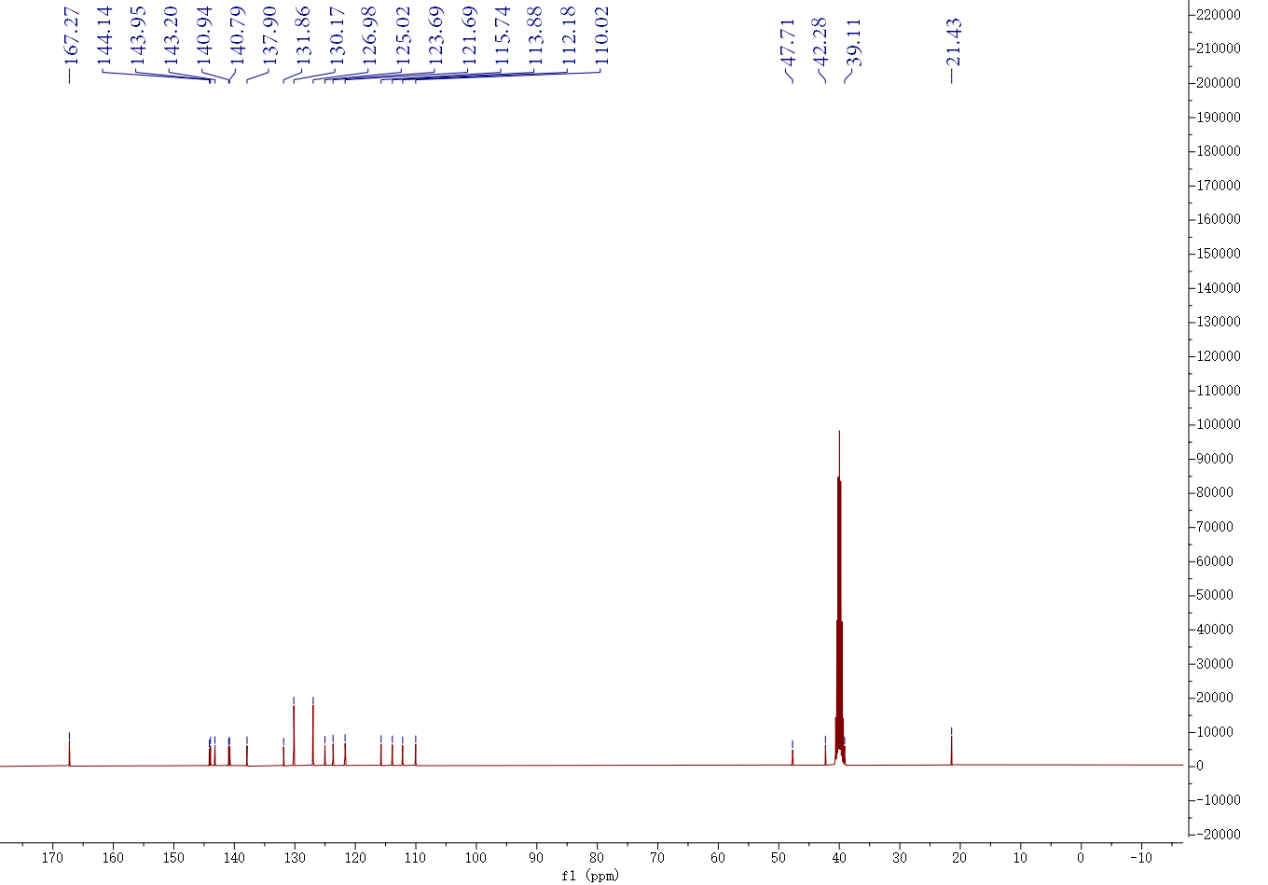
 ^13^C NMR spectrum of PXZ-ER in DMSO-d6


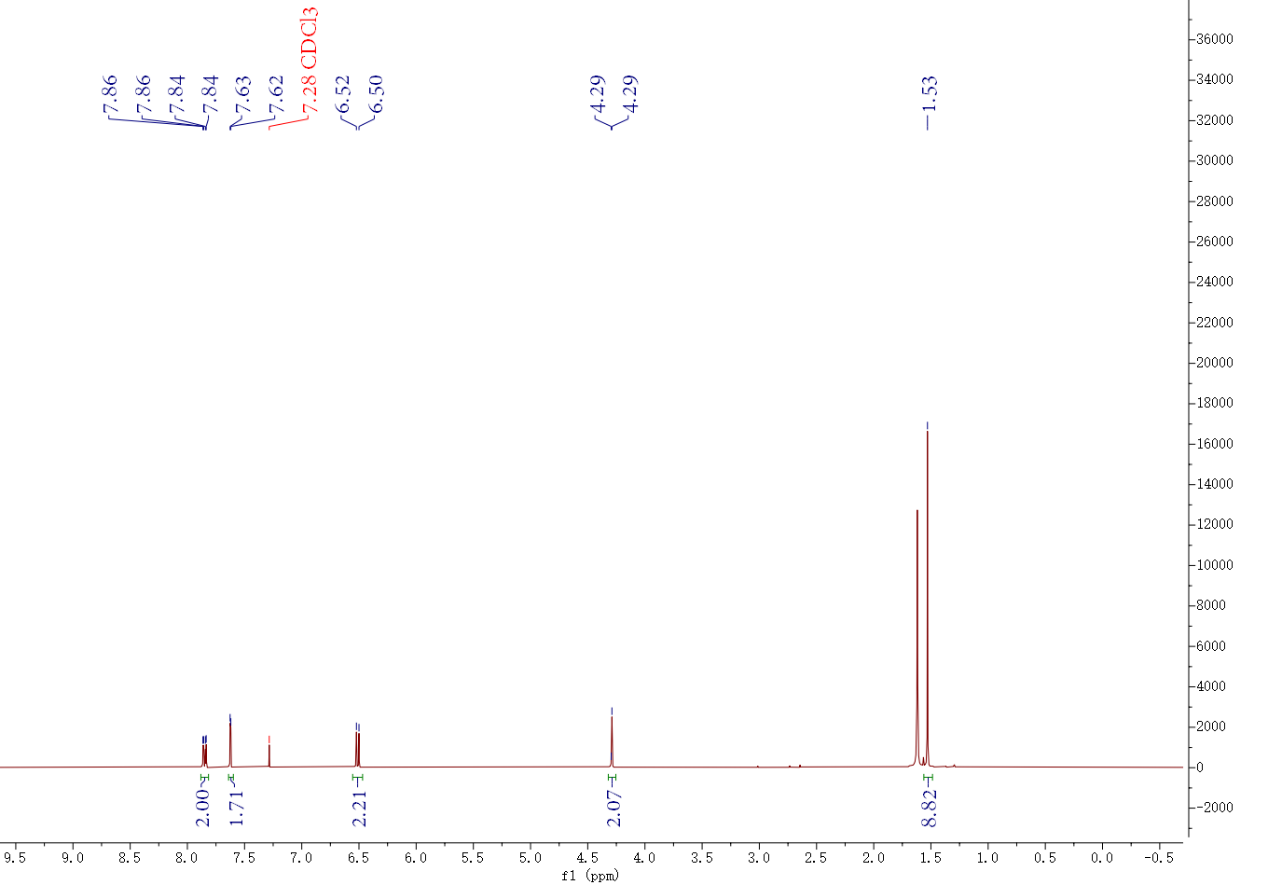


^1^H NMR spectrum of PXZ2N-Lipid (3d) in CDCl_3_


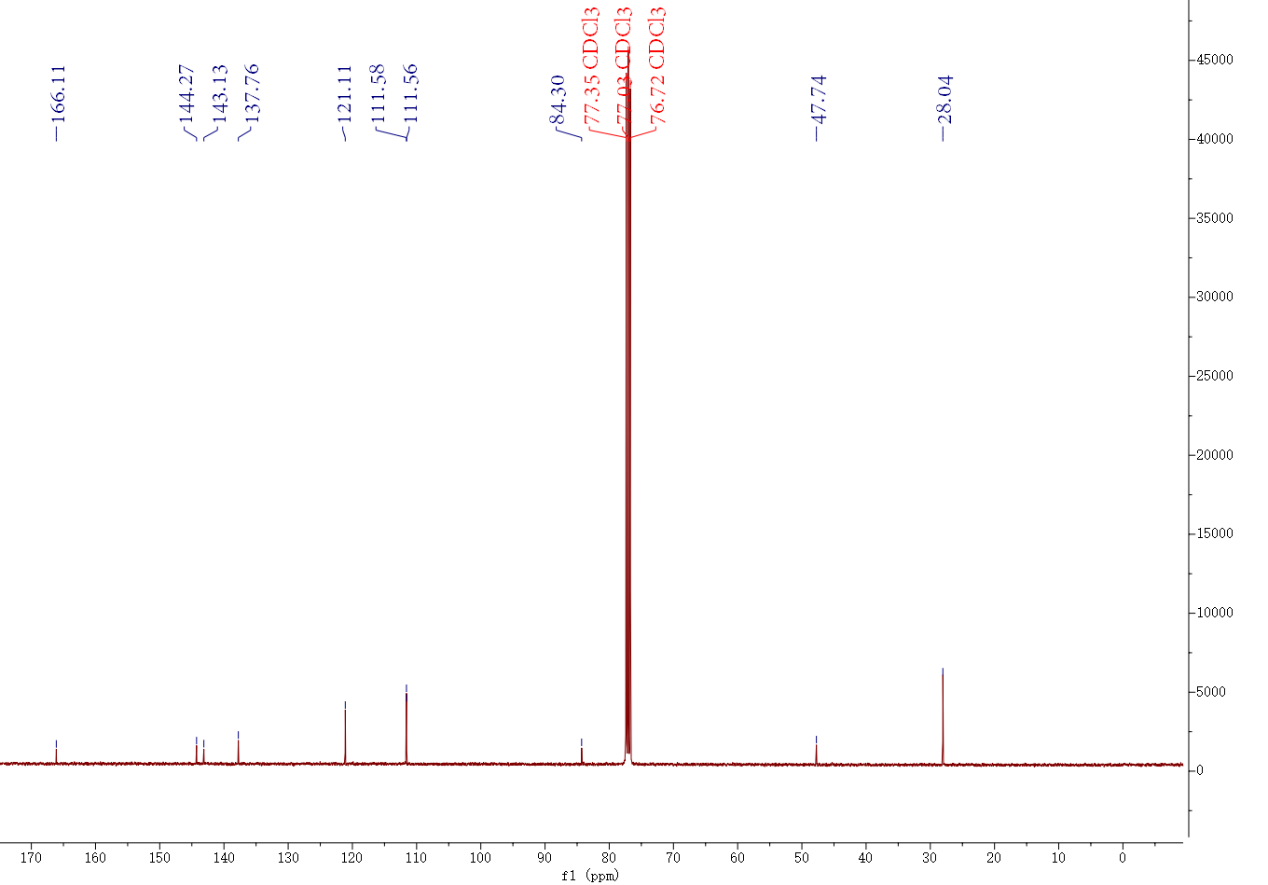


^13^C NMR spectrum of PXZ2N-Lipid (3d) in CDCl_3_


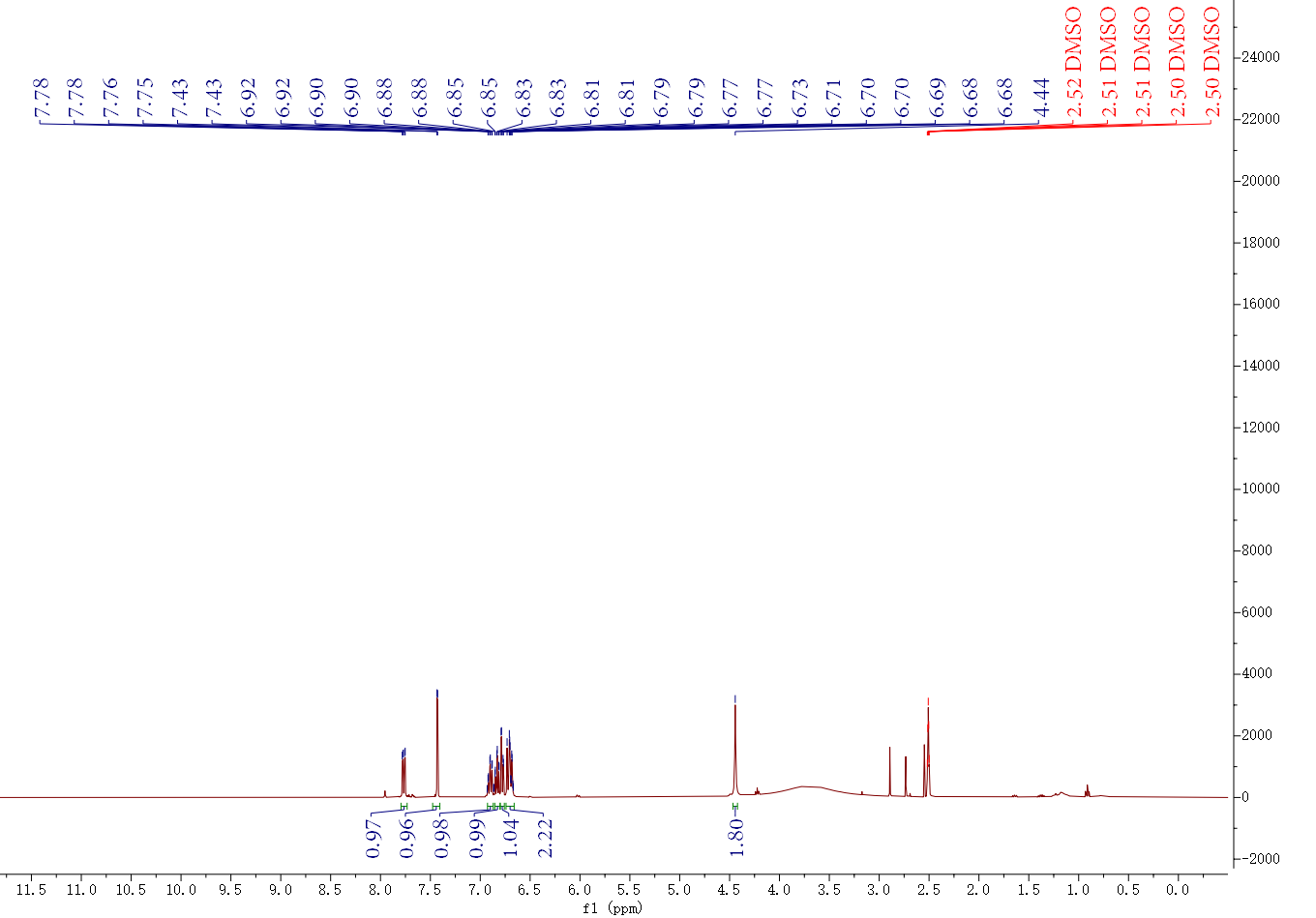


^1^H NMR spectrum of 4d in DMSO-d6


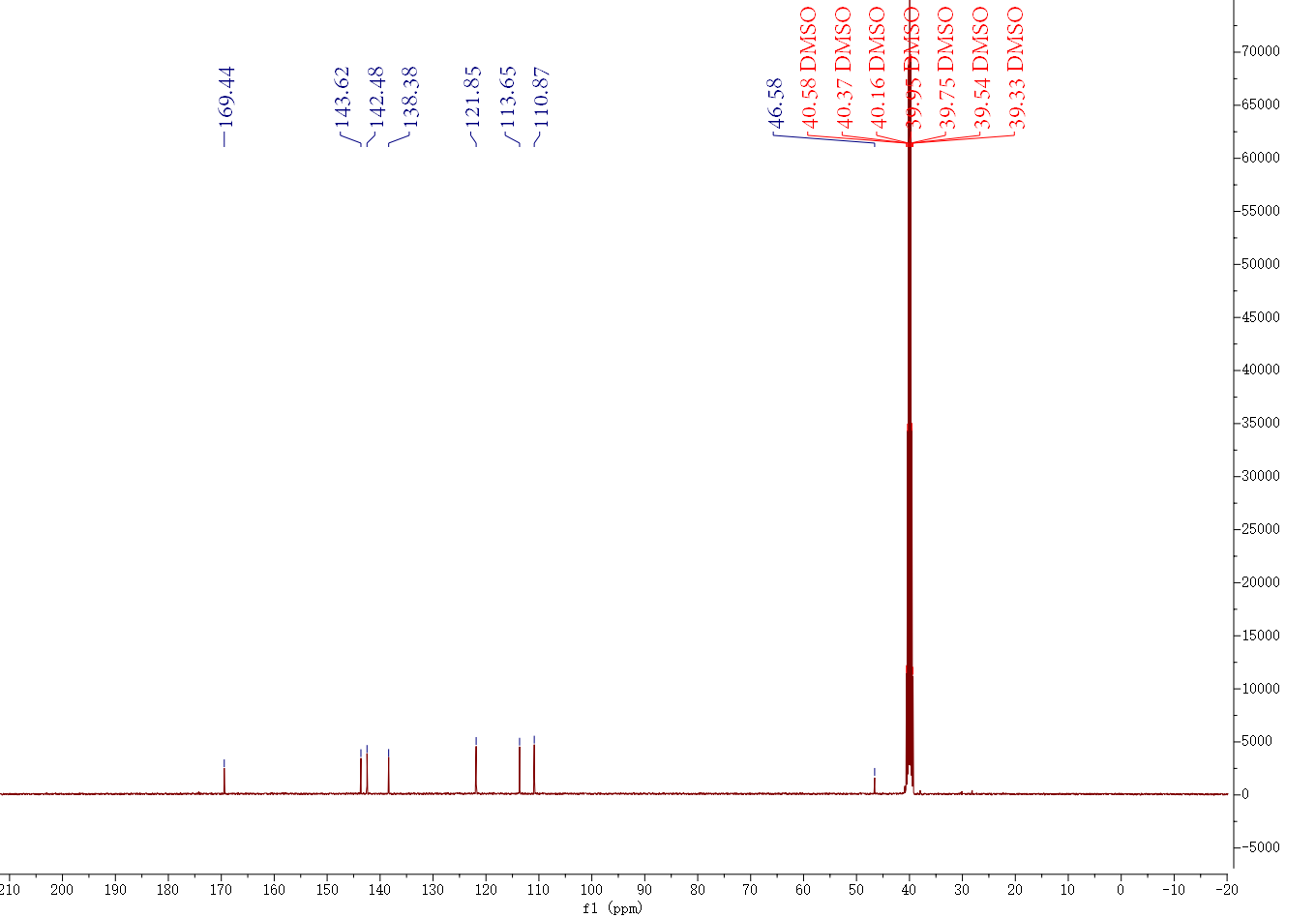


^13^C NMR spectrum of 4d in DMSO-d6


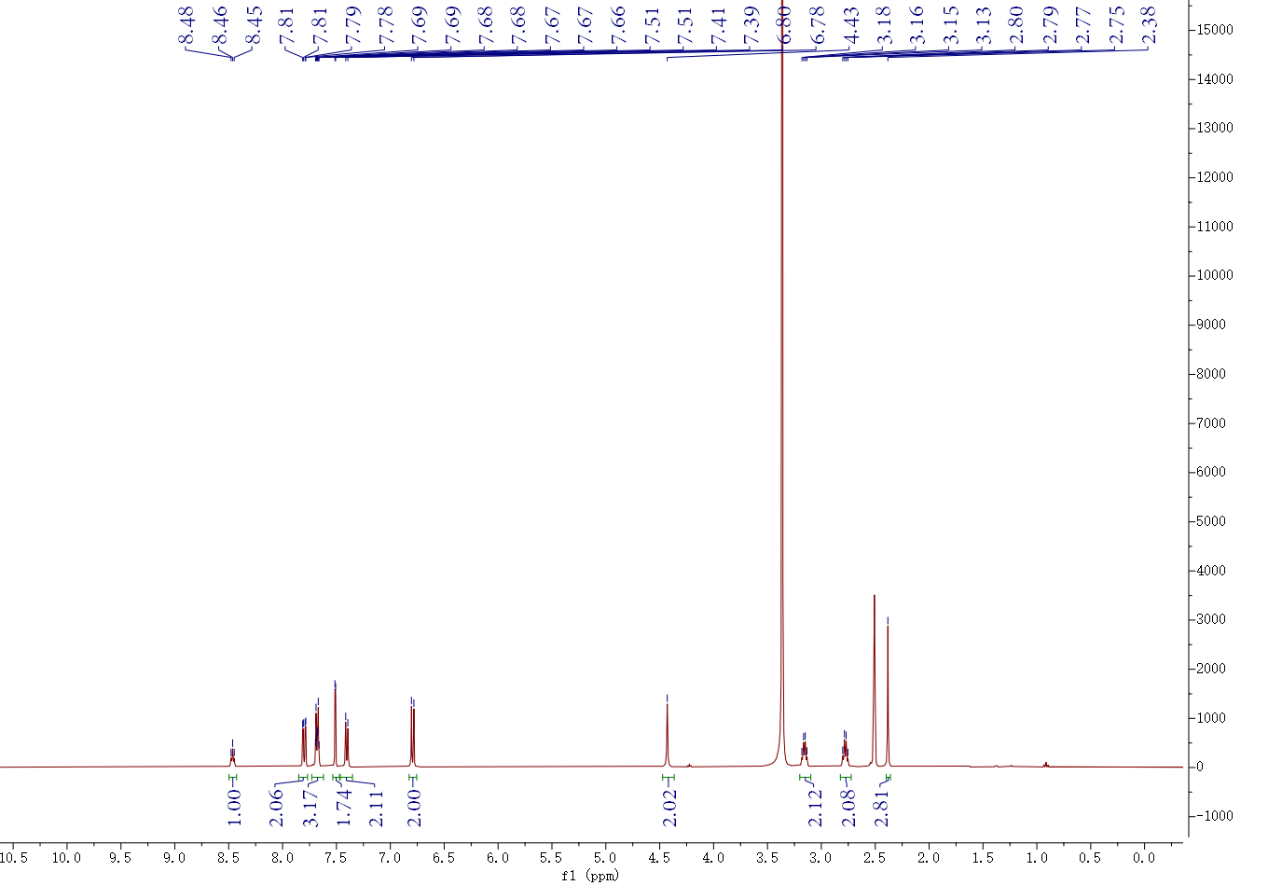


^1^H NMR spectrum of PXZ2N-ER in DMSO-d6


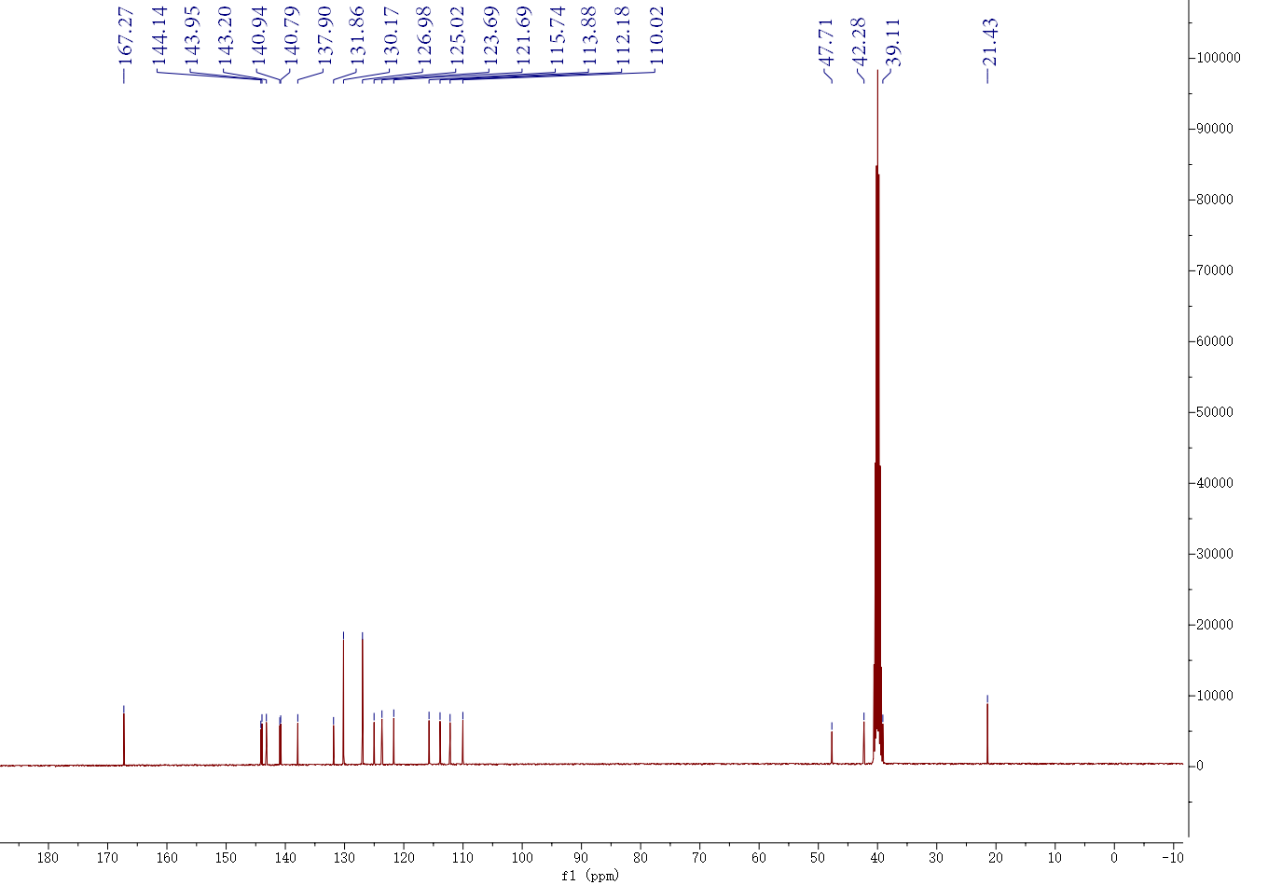
 ^13^C NMR spectrum of PXZ2N-ER in DMSO-d6

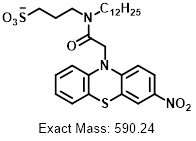

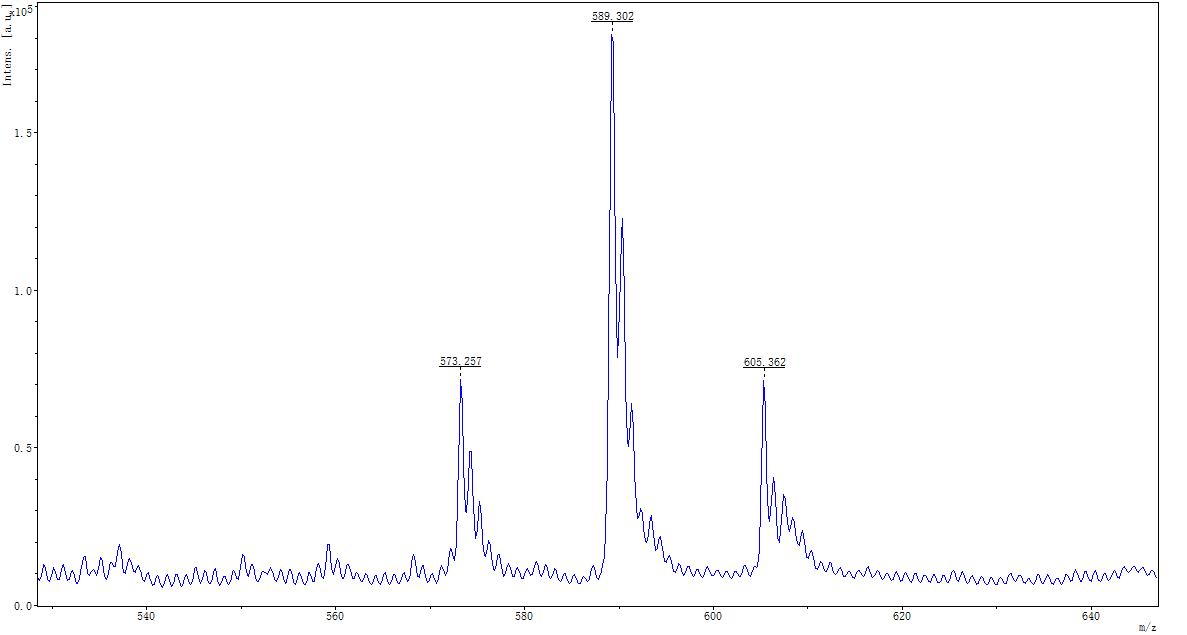

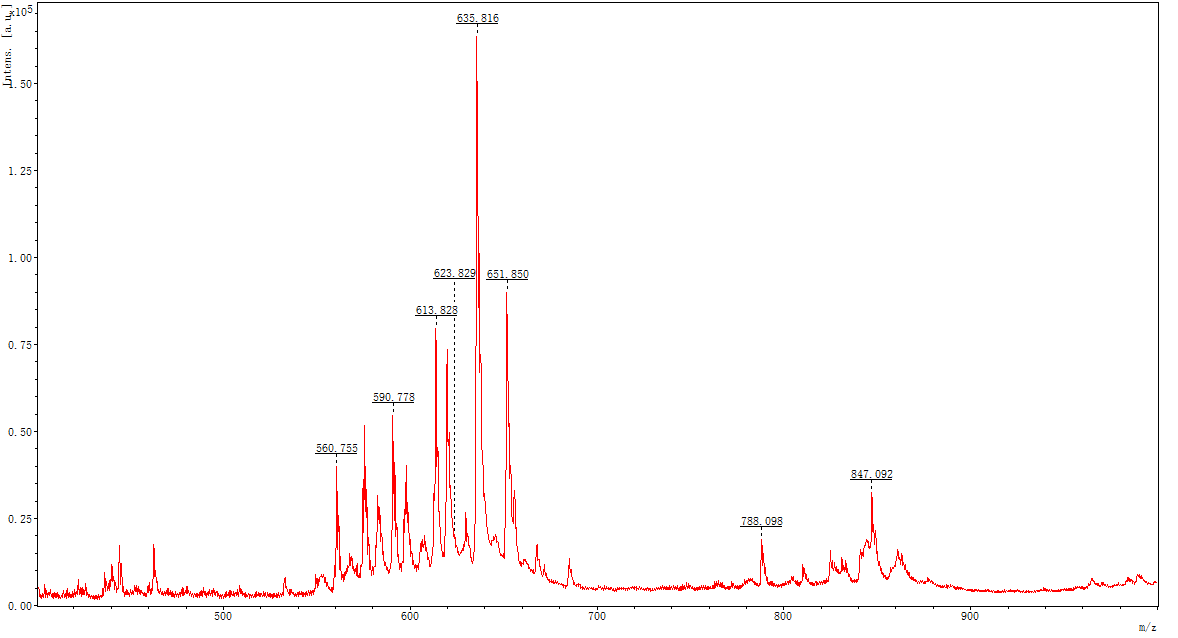

Supplement: Multimedia component 1 [file mmc1.docx]
